# Association of Cancer Incidence and Duration of Residence in Geothermal Heating Area in Iceland: An Extended Follow-Up

**DOI:** 10.1371/journal.pone.0155922

**Published:** 2016-05-20

**Authors:** Adalbjorg Kristbjornsdottir, Thor Aspelund, Vilhjalmur Rafnsson

**Affiliations:** 1 Centre of Public Health Sciences, University of Iceland, Reykjavik, Iceland; 2 Department of Preventive Medicine, Faculty of Medicine, University of Iceland, Reykjavik, Iceland; Hunter College, UNITED STATES

## Abstract

**Background:**

Residents of geothermal areas have higher incidence of non-Hodgkin’s lymphoma, breast cancer, prostate cancer, and kidney cancers than others. These populations are exposed to chronic low-level ground gas emissions and various pollutants from geothermal water. The aim was to assess whether habitation in geothermal areas and utilisation of geothermal water is associated with risk of cancer according to duration of residence.

**Methods:**

The cohort obtained from the census 1981 was followed to the end of 2013. Personal identifier was used in record linkage with nation-wide emigration, death, and cancer registries. The exposed population, defined by community codes, was located on young bedrock and had utilised geothermal water supply systems since 1972. Two reference populations were located by community codes on older bedrock or had not utilised geothermal water supply systems for as long a period as had the exposed population. Adjusted hazard ratio (HR), 95% confidence intervals (CI) non-stratified and stratified on cumulative years of residence were estimated in Cox-model.

**Results:**

The HR for all cancer was 1.21 (95% CI 1.12–1.30) as compared with the first reference area. The HR for pancreatic cancer was 1.93 (1.22–3.06), breast cancer, 1.48 (1.23–1.80), prostate cancer 1.47 (1.22–1.77), kidney cancer 1.46 (1.03–2.05), lymphoid and haematopoietic tissue 1.54 (1.21–1.97), non-Hodgkin´s lymphoma 2.08 (1.38–3.15) and basal cell carcinoma of the skin 1.62 (1.35–1.94). Positive dose-response relationship was observed between incidence of cancers and duration of residence, and between incidence of cancer and degree of geothermal/volcanic activity in the comparison areas.

**Conclusions:**

The higher cancer incidence in geothermal areas than in reference areas is consistent with previous findings. As the dose-response relationships were positive between incidence of cancers and duration of residence, it is now more urgent than before to investigate the chemical and physical content of the geothermal water and of the ambient air of the areas to detect recognized or new carcinogens.

## Introduction

Environmental pollution and its impact on human health have been considered a serious problem in active volcanic areas and millions of people globally live within those areas [1, 2].

During eruption and post-eruptive phases, volcanoes release numerous hazardous contaminants, including toxic gases and heavy metals [3]. People living in the close vicinity of the volcano are usually those who suffer most in cases of eruption [4]; and people living on volcanic ground may experience long-term exposure to various toxic ground gas emissions, carbon dioxide (CO_2_), hydrogen sulphide (H_2_S), radon (Rn), sulphur dioxide (SO_2_), sulphuric acid (H_2_SO_4_), hydrogen chloride (HCl), and hydrogen fluoride (HF), which are considered to pose chronic health hazards [5–7]. Several other low-dose exposures have been mentioned, among them arsenic (As), lead (Pb), and mercury (Hg) [3, 8]. The same chemical components are emitted from geothermal fields and fumaroles in Iceland, namely CO_2_, H_2_S, SO_2_ and hydrogen (H_2_), and trace elements such as As (1–2 ppb), Hg (0.05 ppb), and Rn (3–100 Bq/l) [9–11] have been identified in the geothermal water.

Long-term studies on populations living on geothermal fields or in volcanic areas are scant and cancer incidence among these populations has so far been the subject of only limited study with inconsistent results [8, 12–14], with the exception of the Icelandic studies and a study from Sicily that show similar results [15–17]. In the study from Rotorua, New Zealand, Bates et al. found an increased risk of nasal and lung cancer among residents in a geothermal field, who were exposed to H_2_S [13]. The study from the Azores, Portugal, found an association of female breast cancer and residence on an actively degassing geothermal field, and the authors suggested that trace elements and high Rn exposure might play a role [8]. In a study from Sicily, residents of the volcanic region of Catania province have higher incidence of thyroid cancer than other populations and it is mentioned that the environmental concentration of Rn is elevated in the area; however, it was not possible to conclude on the role of the Rn in this context [14]. In a new study from Sicily, Russo et al. [17] found an increased risk of thyroid cancer and lymphatic leukaemia in men and women; Hodgkin’s lymphoma, stomach and breast cancer were higher among women and prostate cancer among men. The authors conclude that increased risk of all cancers and for several tissue-specific types of cancer was a likely consequence of non-anthropogenic environmental pollution, and different cancer-specific carcinogens and mechanisms may be responsible for the higher incidence of certain tumour types among residents in the volcanic area [17]. In two Icelandic studies [15, 16] a higher risk of non-Hodgkin’s lymphoma (NHL), breast cancer, prostate cancer, and basal cell carcinoma (BCC) of the skin were found. The same pattern emerged in both these studies. The authors advocated the measurement and detection of possible carcinogens in the gas emissions in the geothermal areas and in the geothermal water used for space heating, washing and bathing [15, 16].

The aim of the study is to assess whether cumulative length of residence in a geothermal heating area, where the inhabitants are exposed through use of the geothermal water for space heating, washing, and bathing, and through the ground gas emissions of geothermal fields in their vicinity, is associated with the risk of cancer.

## Methods

Iceland is located in the middle of the North Atlantic Ocean on the Mid-Atlantic Ridge where the North American and Eurasian tectonic plates are moving apart. These movements can be observed in Iceland, and they are related to the volcanic activities in the country. Through the centuries, volcanic eruptions in Iceland have periodically emitted ash and gases, which have been carried downwind to mainland Europe. Historically, such events have been associated with climate change and increased mortality in England and elsewhere [18].

This is a population-based observational study and the source of data was the 1981 National Census in Iceland. The four-digit community codes in the census (Table A in S1 File) were used to define the exposed population as inhabitants of communities that have used geothermal heating supply systems since 1972 or earlier, according to description of all hot water supply systems in Iceland, and the National Registry [11, 19]. In these communities, geothermal water has been used for domestic and greenhouse heating, laundry, bathing, showering, and washing, in spas and swimming pools; however, geothermal water has not been used as drinking water. The geothermal water comes from drilled boreholes that can be up to several hundred meters deep. The water temperature at source ranges from 70°C to 120°C [11]. The geothermal supply distribution systems consist of a network of pipes conducting the water from the boreholes to serve each of the homes and other buildings in the respective community, with few exceptions; the main feeding pipe for the communities can be up to 20 km long. The communities in geothermal heating areas were all located in the central region of the country where the bedrock is less than 3.3 million years old, and some of these communities were on or near even younger bedrock, less than 0.8 million years old. The two reference populations were also identified by the community codes in the census [19]; they had not utilised geothermal heating systems as old as 1972 [11], and age of the bedrock was also taken into consideration [20]. The first of these two reference populations, called the cold reference area, included residents of communities located in the west and east parts of Iceland where the bedrock is more than 3.3 million years old and up to 15 million years old. These communities are well outside the volcanic zone in the central region of the country. The population of the cold reference area is considered the main comparison population in the study. The second reference population, in the area referred to as the warm reference area, included residents of communities located in the central region of the country, where the age of the bedrock is variable but ranging from very young (less than 0.8 million years to 15 million years old). The populations in the area of the capital, Reykjavik, and the adjacent Reykjanes area were not included in the study in order to avoid bias due to capital effect [21]. To summarise: with respect to the age of the bedrock there is a volcanic/geothermal gradient through the areas, lowest in the cold area, middling in the warm area, and highest in the geothermal heating area, with reference to geological studies [22,23], and in that area the communities had had geothermal water supply systems since 1972 or earlier [11]. With the passage of time, it is becoming increasingly difficult to obtain populations in Iceland unexposed to geothermal water, as approximately 90% of all houses and swimming pools are at present heated with geothermal water and 12% of the electricity is generated from geothermal power plants [11]. The exposed area and reference areas were the same as used in the previous mortality study [20].

The characteristics of the populations have been described in previous studies [15, 16, 20]. Briefly, eligible participants were people aged 5–64 years. The census included information on personal identification number, gender, age, residence, education, and type of housing, and these two last mentioned variable are the indicators of the socioeconomic status. Personal identification numbers were used in record linkage with the National Registry to obtain information on possible out-migration and with the National Cause-of-Death Registry to obtain information on vital status and, where applicable, the date of death according to death certificates. These registries are kept at Statistics Iceland. By out-migration, we mean those who have moved abroad, not to be confused with those who have moved domestically from one community to another (see later discussion on place, and length of residence in the study areas). After a person has moved abroad, an eventual cancer diagnosis is not necessarily registered in the Cancer Registry, and due to that uncertainty, that person’s follow-up has to be censored at the day of out-migration.

The cancer cases in the study populations were identified by record linkage of the personal identification number with the Cancer Registry (a nation-wide registry of all cancer cases with virtually complete coverage and over 95% of the diagnoses histologically verified) [24]. Thus from the Cancer Registry we obtained information on the cancer site, morphology, and year of diagnosis. BCC has been registered in a special file at the Cancer Registry; it is not counted with the overall cancers, and is analysed separately.

Information on smoking was not collected in the census and thus not available on an individual level. Since 1985, the Public Health Institute of Iceland has collected results from annual surveys on smoking habits among random samples of the population according to gender and postal codes [25]. The estimation of smoking habits on community and gender level was done according to the same procedure as has been described in previous studies [16, 20].

Information on reproductive factors was not available from the census. The reproductive factor, age at first birth, was estimated according to information from Statistics Iceland [19], and in the same manner as in previous studies [16, 20].

Statistics Iceland publishes annually the National Roster of the population with personal identification numbers, addresses and community codes. The information on the duration of residence was obtained from the available National Rosters from the years 1985, 1990, 1995, 2000 and 2004. If the location of an individual was in the same community in the census as in the National Rosters (1985, 1990 ….2004), it was assumed that the person had been resident in that community for the number of years between the census and the record of that person in the respective roster. In cases where the individual was not located in the same community in the roster 1985 as in the census 1981, the cumulative number of years of residence was estimated to be less than 5 years. In cases where the individual was located in the same community in the roster 1985 as in census 1981 but not in the same community in the roster 1990 (and so on through the rosters), the cumulative number of years of residence was estimated to be 5 years. The last residence category was estimated to have cumulative years of residence of 24 years or more. There were thus six categories of cumulative residence: less than 5 years, 5 years, 10 years, 15 years, 20 years, and 24 years or more, for the exposed and the reference populations.

Follow-up time started at the day of census, 31 January, 1981, and continued to the date of out-migration, or the date of death, or the date of first diagnosed cancer, or 31 December 2013 (the end of the follow-up period), whichever occurred first. Immortal person-time was taken into consideration, and excluded, which means that from follow-up time allocated to a specific exposure category, we excluded time during which the exposure-category definition was being met, according to Rothman and Greenland [26].

Regardless of exposure categories, survival for the event-free proportion was shown for the geothermal heating area and the cold reference area by Kaplan-Meier estimates for all cancers, breast cancer, prostate cancer, pancreatic cancer and NHL [27].

The plots of the log(-log(survival)) versus log(time) curves were created for exposed group/warm reference group and for exposed group/cold reference group to observe whether resulting in parallel curves or not. By the introduction of an interaction term of the covariate with time the proportional hazard assumption was checked by testing for significance.

The Cox proportional hazard model was used to estimate hazard ratio (HR) and 95% confidence intervals (95% CI) for all cancers and selected cancer site [28]. Covariates were age, gender, educational level, type of housing, smoking habits, and reproductive factors.

The exposed population living in the geothermal heating area was compared with the other populations (warm reference area and cold reference area) in separate analyses. Several calculations were done in the model: crude comparison without any adjustments, comparison with adjustment for age and gender only, and with adjustment for age, gender, educational level, housing, and smoking habits. These calculations had nearly identical results. Then we did analyses with stratification on categories of cumulative years of residence, and then in a separate analysis where five-year latency was applied. Only the results with all adjustment without and with stratification on cumulative years of residence and without and with five-year latency are presented here; however detailed results are shown in Appendix. Separate analyses were done after dividing the material by gender and by splitting the material by four strata on categories of cumulative years of residence and by different age strata, without and with five-year latency.

Due to concerns that variation in prevalence of mutation in the BRCA2 gene across the three study populations might account for our results, we used the method of Axelson and Steenland [29] to evaluate the possible confounding due to this unmeasured genetic factor. Axelson and Steenland introduced their method for evaluating potential confounding effect of smoking in occupational studies, however, the method has been widely used, for example to evaluate confounding due to reproductive factors for the risk of female breast cancer [30]. Mutation in the BRCA2 gene has been studied in families with high risk of breast cancer in both female and male in Iceland [31], and this mutation was detected in 0.6% of the population (based on a random sample), in 7.7% of female breast cancer cases, and in 40% of males with breast cancer. The total 38 cases of males with breast cancer in the census 1981 (N = 184 114, followed from 1981 to 2013), were used to calculated the prevalence of those with and without the mutation in the BRCA2 gene in the three study populations, based on the number of male breast cancer patients, three in the geothermal heating area (n = 7 511), nine in the warm (n = 44 864), and five in the cold (n = 22 431) reference areas. For the mutation in the BRCA2 gene, we assumed that the mutation was the only reason the population in the geothermal heating area had an elevated risk of breast cancer among females. We estimated the geothermal population expected female breast cancer incidence rate [29] using the prevalence in the groups with and without the mutation, and known female breast cancer relative risk comparing those with and without the mutation. These risks were obtained from Thorlacius et al. [32] (modified to take age into account): the risk of breast cancer in a population of BRCA2 carriers as 7, relative to that of a population free from BRCA2 mutation set as 1. This rate was compared with the expected female breast cancer incidence rate in the populations in the warm and cold reference areas, which had been estimated using analogous fractions, and corresponding female breast cancer risk of these populations.

The statistical analyses were performed using the PASW (SPSS) software version 22.

The National Bioethics Committee (VSNb2010060005/03.1) and the Data Protection Commission (2010060524ÞPJ/—) approved the study.

## Results

The national census in 1981 included 184,114 individuals or 99.2% of the population aged between 5 and 64 years according to the National Registry [19]. At the end of the follow up on 31 January 2013, 58.4% of individuals in the 1981 census were still alive, had not out-migrated, and were without cancer. Over the studied period, 10.9% of the populations had died, 17.6% had out-migrated, and 13.1% had been diagnosed with first cancer. A total of 24,136 persons were diagnosed with first cancer during the 33 years of the follow up.

The baseline characteristics in the three study populations: geothermal heating area, the warm, and the cold reference areas, are shown in Table 1, and Table B in S1 File.

**Table 1:** Baseline characteristics in the geothermal heating area and the two reference areas, categories of cumulative years of residence in the respective areas, number of individual, and age at census.

	Geothermal heating area	Warm reference area	Cold reference area
	N (%)	N (%)	N (%)
Number of people	7511 (100)	44 864 (100)	22 431 (100)
**Age in years**			
Mean ± SD	28.81 ± 16.35	28.65 ± 16.30	28.56 ± 16.20
Median, IQR (0.25; 0.75)	26 (15; 41)	26 (15; 40)	26 (15; 40)
**Numbers in categories of cumulative years of residence**			
< 5 years	1479 (19.7)	6935 (15.5)	4029 (18.0)
≥ 5 to <10 years	1061 (14.1)	5808 (12.9)	3213 (14.3)
≥ 10 to < 15 years	843 (11.2)	5688 (12.7)	2853 (12.7)
≥ 15 to < 20 years	671 (8.9)	3829 (8.5)	1880 (8.4)
≥ 20 to < 24 years	492 (6.6)	3235 (7.2)	1861 (8.3)
≥ 24 years	2965 (39.5)	19 369 (43.2)	8595 (38.3)
**Age (years) at census in categories of cumulative years of residence**			
< 5 years			
Mean ± SD	26.83 ± 14.78	26.71 ± 14.93	26.16 ± 14.54
Median, IQR (0.25; 0.75)	23 (17; 34)	24 (17; 34)	24 (16; 33)
≥ 5 to <10 years			
Mean ± SD	24.67 ± 15.86	25.09 ± 15.66	25.39 ± 15.77
Median, IQR (0.25; 0.75)	19 (14; 33)	20 (14; 34)	20 (14; 33)
≥ 10 to < 15 years			
Mean ± SD	25.48 ± 17.09	26.92 ± 17.25	27.02 ± 17.56
Median, IQR (0.25; 0.75)	18 (12; 36)	20 (13; 39)	20 (13; 40)
≥ 15 to < 20 years			
Mean ± SD	27.93 ± 20.24	27.88 ± 19.86	26.96 ± 19.20
Median, IQR (0.25; 0.75)	19 (9; 48)	22 (9; 47)	21 (10; 44)
≥ 20 to < 24 years			
Mean ± SD	31.75 ± 18.79	31.07 ± 19.14	30.24 ± 18.28
Median, IQR (0.25; 0.75)	32 (13; 49)	30 (12; 49)	28 (13; 46)
≥ 24 years			
Mean ± SD	31.95 ± 14.88	30.66 ± 14.97	31.38 ± 14.86
Median, IQR (0.25; 0.75)	31 (20; 43)	30 (19; 42)	30 (20; 42)

Abbreviations: SD standard deviation, IQR inter-quartile range.

**Fig 1:**
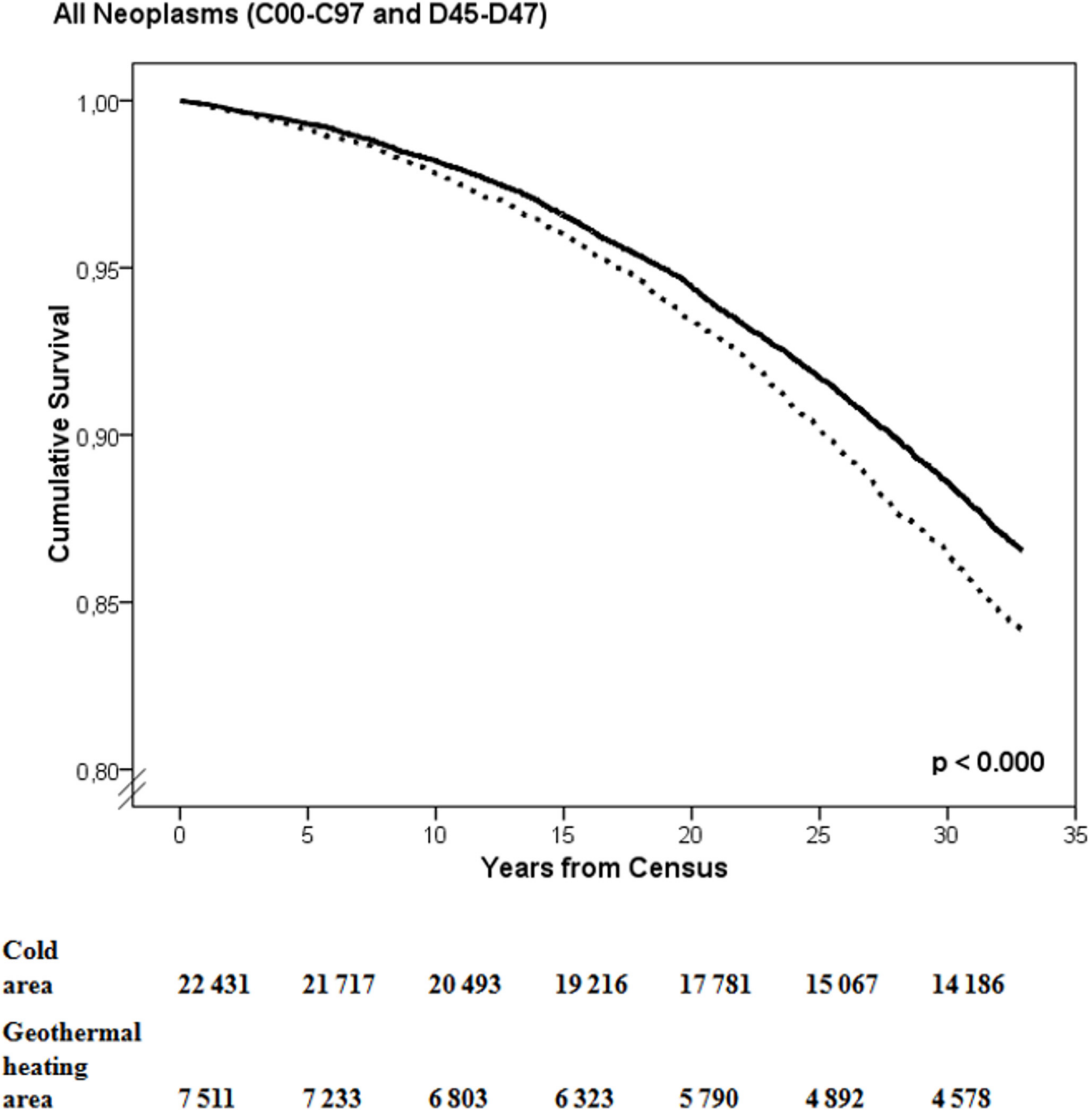
Kaplan-Meier estimates of event free proportion for all cancers since the census 1981, dashed line indicate population in geothermal heating area, and black line population in the cold reference area.

Fig 1 shows the result of the Kaplan-Meier estimates illustrating the time until any first cancer reported to the Cancer Registry in the geothermal heating area and the cold reference area. A greater proportion of the inhabitants of geothermal heating area were diagnosed with cancer than among the inhabitants of the cold reference area at each time point, and the curves never crossed during the study period.

**Fig 2:**
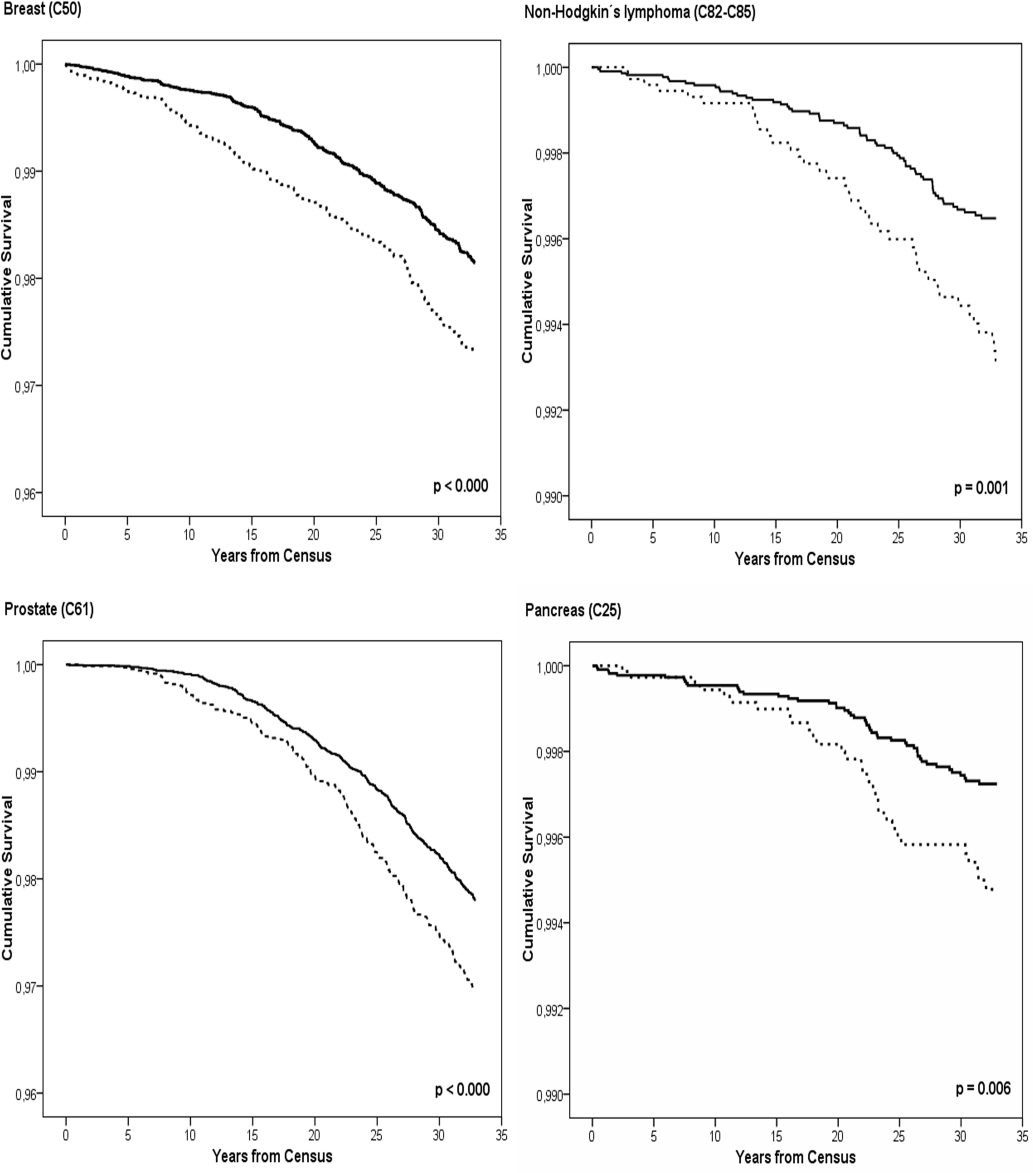
Kaplan-Meier estimates of event free proportion for breast cancer, prostate cancer, pancreas cancer and non-Hodgkin’s lymphoma (NHL) since the census 1981. Dashed line indicates population in geothermal heating area, and black line population in the cold reference area.

Fig 2 shows results of the Kaplan-Meier estimates illustrating the time until first occurrence of breast cancer, prostate cancer, NHL, and pancreatic cancer in the geothermal heating area and in the cold reference area.

The plots of the log(-log(survival)) versus log(time) curves did not cross and were reasonably parallel, shown in S1 and S2 Figs. The proportional hazard assumption for the Cox model (exposed group/warm reference group) was held (p = 0.76), and same for the other model (exposed group/cold reference group) (p = 0.61), so the Cox regression model were considered appropriate.

Table 2 shows the number of all cancers, and selected cancer sites among the combined genders in the study populations, and the HR and 95% CI adjusted for age, gender, education, type of housing, and smoking habits, both without and with stratification into categories of cumulative residence.

**Table 2:** Number of all cancers and selected cancer sites among men and women combined, hazard ratio (HR), 95% confidence intervals (CI) compared with the populations in warm reference area and cold reference area, adjusted for age, gender, education, type of housing, and smoking habits, without and with stratification into categories of cumulative years of residence in the respective areas.

	Geothermal heating area	Warm reference area					Cold reference area				
	No of cancers	No of cancers	Not stratified		Stratified		No of cancers	Not stratified		Stratified	
Cancers (ICD-10)			HR	95%CI	HR	95%CI		HR	95%CI	HR	95%CI
All (C00-C97, D45-D47)	988	5331	1.06	0.99–1.14	1.10^**^	**1.02**–**1.18**	2524	1.17^***^	**1.09**–**1.26**	1.21^***^	**1.12**–**1.30**
Pancreas (C25)	30	126	1.50	0.99–2.27	1.53^*^	**1.00**–**2.32**	49	1.87^**^	**1.18**–**2.95**	1.93^**^	**1.22**–**3.06**
Lung and bronchus (C33-C34)	92	578	0.91	0.72–1.14	0.94	0.75–1.18	300	0.92	0.73–1.17	0.96	0.76–1.22
Breast (C50)	161	740	1.23^*^	**1.03**–**1.46**	1.27^**^	**1.07**–**1.52**	326	1.42^***^	**1.18**–**1.72**	1.48^***^	**1.23**–**1.80**
Prostate (C61)	172	803	1.27^**^	**1.07**–**1.51**	1.32^***^	**1.11**–**1.57**	377	1.43^***^	**1.19**–**1.72**	1.47^***^	**1.22**–**1.77**
Kidney (C64-C66)	49	241	1.21	0.88–1.67	1.27	0.92–1.75	103	**1.40**	**1.00**–**1.97**	1.46^*^	**1.03**–**2.05**
Lymphoid and haematopoietic tissue (LH) (C81-C96, D45-D47)	97	419	1.30^*^	**1.03**–**1.64**	1.36^**^	**1.08**–**1.72**	199	1.49^***^	**1.17**–**1.91**	1.54^***^	**1.21**–**1.97**
Non-Hodgkin´s lymphoma (NHL) (C82-C85)	39	137	1.78^***^	**1.22**–**2.59**	1.90^***^	**1.30**–**2.77**	62	2.00^***^	**1.33**–**3.03**	2.08^***^	**1.38**–**3.15**
NHL, peripheral T-cells (C84)	6	14	2.85^*^	**1.02**–**7.99** ^b^	2.91^*^	**1.04**–**8.19** ^b^	5	3.84^*^	**1.11**–**13.31** ^b^	3.93^*^	**1.12**–**13.74** ^a^
NHL, unspecified (C85)	9	11	5.13^***^	**1.99**–**13.21** ^a^	5.23^***^	**2.02**–**13.54** ^a^	4	6.81^***^	**2.03**–**22.80** ^a^	6.75^***^	**2.01**–**22.64** ^a^
Leukaemia (C91-C95, D45-D47)	30	145	1.10	0.73–1.65	1.13	0.75–1.70	71	1.30	0.85–2.00	1.37	0.89–2.11
Chronic lymphocytic leukaemia (CLL)(C91.1)	10	45	1.16	0.57–2.35	1.22	0.59–2.49	24	1.30	0.62–2.73	1.45	0.68–3.08
Non-CLL (C91-C95, D45-D47, except C91.1)	20	100	1.07	0.65–1.76	1.09	0.67–1.80	47	1.31	0.77–2.22	1.35	0.80–2.29
Myelodysplastic syndromes (MDS) (D46)	8	18	2.31	0.95–5.58	2.44^*^	**1.01**–**5.90** ^b^	6	4.02^*^	**1.38**–**11.71** ^a^	4.07^*^	**1.39**–**11.93** ^a^
MDS, unspecified (D46.9)	8	12	3.41^*^	**1.32**–**8.83** ^a^	3.70^**^	**1.42**–**9.64** ^a^	3	8.20^***^	**2.10**–**32.10** ^a^	8.09^***^	**2.06**–**31.79** ^a^
Other LH, uncertain (D47)	3	14	1.24	0.34–4.54	1.41	0.38–5.21	7	1.21	0.31–4.70	1.14	0.29–4.44
Other LH thrombocythemia (D47.3)	3	2	11.43^*^	**1.64**–**79.80** ^b^	12.72^*^	**1.80**–**89.74** ^b^	1	8.61	0.85–86.98	7.53	0.74–76.48
Not included in all cancers											
Basal cell carcinoma of skin (C44)	177	781	1.22^*^	**1.03**–**1.45**	1.28^***^	**1.08**–**1.52**	335	1.54^***^	**1.28**–**1.85**	1.62^***^	**1.35**–**1.94**

Statistically significant HRs are bolded.

^a^ 95%CI computed with bootstrap method did not include unity.

^b^ 95%CI computed with bootstrap method included unity.

^*^ p < 0.05

^**^ p < 0.01

^***^ p < 0.005.

For completeness, all cancer sites with any cancer case in the geothermal heating area are shown in Table C in S1 File, and sites with no case are not shown. The HRs were generally higher in comparison with the cold reference area than with the warm reference area, and were higher when stratified on categories of cumulative years of residence than without such stratification; this was valid in comparison with the cold and warm reference areas. The HRs were higher for all cancers and several of the selected cancer sites, including cancers of pancreas, breast, prostate, and kidney, and the combined cancers of the lymphoid and haematopoietic tissue, counting NHL, and myelodysplastic syndromes (MDS) (Table 2). In the analyses, the HRs for lung cancer were, 0.91 to 0.96, in comparison with the warm and cold reference areas respectively, and the 95% CI included unity. The HRs for BCC were also higher, and showed a similar pattern as for other selected cancer sites shown in Table 2, namely the HRs were higher in comparison with the cold than with the warm reference areas, and the HRs were higher when stratified on categories of cumulative years of residence than without such stratification; this was valid in comparison with the two reference areas.

Table 3 shows the number of all cancers, and selected cancer sites in the study populations when applying five-year latency time. The HR and 95% CI were analysed without and with stratification on cumulative residence, and adjusted for age, gender, education, type of housing, and smoking habits.

**Table 3:** Number of all cancers and selected cancer sites among men and women combined, hazard ratio (HR), 95% confidence intervals (CI) compared with the populations in warm reference area and cold reference area applying five years latency time, adjusted for age, gender, education, type of housing, and smoking habits, without and with stratification into categories of cumulative years of residence in the respective areas.

	Geothermal heating area	Warm reference area					Cold reference area				
	No of cancers	No of cancers	Not stratified		Stratified		No of cancers	Not stratified		Stratified	
Cancers (ICD-10)			HR	95%CI	HR	95%CI		HR	95%CI	HR	95%CI
All (C00-C97, D45-D47)	372	1845	1.11	0.99–1.25	1.16^*^	**1.03**–**1.30**	959	1.19^***^	**1.06**–**1.35**	1.22^***^	**1.08**–**1.37**
Pancreas (C25)	11	36	1.92	0.94–3.91	2.11^*^	**1.03**–**4.34**	19	1.90	0.90–4.00	2.01	0.95–4.25
Lung and bronchus (C33-C34)	40	209	1.01	0.71–4.42	1.03	0.72–1.46	126	0.99	0.69–1.41	1.01	0.71–1.45
Breast (C50)	56	277	1.11	0.82–1.49	1.14	0.85–1.54	133	1.28	0.94–1.75	1.29	0.94–1.76
Prostate (C61)	57	288	1.15	0.85–1.54	1.21	0.90–1.63	138	1.31	0.96–1.79	1.35	0.98–1.85
Kidney (C64-C66)	16	90	1.03	0.59–1.78	1.06	0.61–1.84	41	1.17	0.66–2.09	1.18	0.66–2.10
Lymphoid and haematopoietic tissue (LH) (C81-C96, D45-D47)	37	143	1.49^*^	**1.02**–**2.17**	1.61^*^	**1.10**–**2.36**	72	1.64^*^	**1.10**–**2.44**	1.70^*^	**1.14**–**2.55**
Non-Hodgkin´s lymphoma (NHL) (C82-C85)	17	49	2.12^*^	**1.18**–**3.80**	2.30^**^	**1.27**–**4.14**	20	2.98^***^	**1.50**–**5.89**	3.02^***^	**1.52**–**6.00**
NHL, peripheral T-cells (C84)	4	5	4.22^*^	**1.02**–**17.44** ^b^	4.24^*^	**1.02**–**17.64** ^b^	2	8.77^*^	**1.27**–**60.56** ^a^	8.93^*^	**1.25**–**63.69** ^a^
NHL, unspecified (C85)	3	0					1	12.38^*^	**1.12**–**137.23** ^b^	10.23	0.96–108.93
Leukaemia (C91-C95, D45-D47)	10	50	1.08	0.53–2.18	1.22	0.60–2.49	28	1.11	0.54–2.28	1.25	0.60–2.59
Chronic lymphocytic leukaemia (CLL)(C91.1)	1	15	0.35	0.05–2.71	0.41	0.05–3.21	8	0.40	0.06–2.87	0.59	0.07–4.93
Non-CLL (C91-C95, D45-D47, except C91.1)	9	35	1.40	0.65–3.01	1.58	0.73–3.42	20	1.40	0.64–3.09	1.48	0.67–3.26
Myelodysplastic syndromes (MDS) (D46)	7	10	3.97^*^	**1.40**–**11.29** ^a^	4.17^**^	**1.46**–**11.94** ^a^	2	11.30^***^	**2.25**–**56.67** ^a^	11.46^***^	**2.27**–**57.80** ^a^
MDS, unspecified (D46.9)	7	6	6.48^***^	**2.01**–**20.93** ^a^	7.18^***^	**2.19**–**23.52** ^a^	1	20.59^**^	**2.51**–**169.15** ^a^	20.74^**^	**2.50**–**171.80** ^a^
Other LH, uncertain (D47)	1	1	6.34	0.34–118.41	8.68	0.44–170.13	0				
Other LH thrombocythemia (D47.3)	1	1	6.34	0.34–118.41	8.68	0.44–170.13	0				
Not included in all cancers											
Basal cell carcinoma of skin (C44)	74	357	1.06	0.82–1.37	1.11	0.86–1.44	154	1.44^*^	**1.09**–**1.91**	1.48^**^	**1.12**–**1.96**

Statistically significant HRs are bolded.

^a^ 95%CI computed with bootstrap method did not include unity.

^b^ 95%CI computed with bootstrap method included unity.

^*^ p < 0.05

^**^ p < 0.01

^***^ p < 0.005.

All cancer sites with any cancer case in the geothermal heating area are shown in Tables D, E, and F in S1 File, for completeness, and sites with no case are not shown. As in the analyses without latency there was a similar pattern in Table 3 as in previous Table 2, i.e. the HRs were generally higher in comparison with the cold reference area than with the warm reference area, and were higher when stratified on cumulative residence than without such stratification; this was valid in comparison with the cold and warm reference areas. The HRs for all cancer, pancreatic cancer, and the combined cancers of the lymphoid and haematopoietic tissue, including NHL, and MDS, were higher in these analyses with five-year latency time than without applying the latency. On the contrary, the HRs for breast, prostate, and kidney cancers were high, albeit not as high as in the analyses without latency time, and the accompanying 95% CI included unity. The HRs for BCC were increased when applying five-year latency time; however they were lower than in the analyses without latency time, and only in comparison with the cold reference area did the 95% CI not include unity.

When analysing men separately, 517 cancers had occurred in the geothermal heating area, and the HR for all cancers was 1.07 (95% CI 0.97–1.18) in the comparison with the warm reference area, and 1.21 (95%CI 1.09–1.34) in comparison with the cold reference area, with stratification on cumulative years of residence, and adjusted for age, education, housing, and smoking habits (Table G in S1 File). The HRs for the different cancer sites showed a similar pattern as in the analyses of the genders combined. In Table G in S1 File, all cancer sites with any case among men in the geothermal heating area are shown for completeness.

In the analysis of women separately, 471 cancers had occurred in the geothermal heating area, and the HR for all cancers was 1.14 (95% CI 1.03–1.26) in the comparison with the warm reference area, and 1.21 (95% CI 1.09–1.35) in comparison with the cold reference area, with stratification on cumulative years of residence, and adjusted for age, education, housing, and smoking habits (Table H in S1 File). The HRs for the different cancer sites showed a similar pattern as in the analyses of the genders combined. In Table H in S1 File, all cancer sites with any case among women in the geothermal heating area are shown for completeness.

In a comparison between the study areas, when we split the material according to four categories of cumulative years of residence in the respective areas analysing the cancer risk in these strata separately, and adjusting for age, gender, education, housing, and smoking habits, the HRs were higher in the four strata, and these results are shown in detail in Tables I, and J in S1 File.

Restricting the analyses into different groups according to age in the census, we divided the population into those under 20 years of age, under 25 years, and so on in incremental 5-year age groups, up to under 40 years of age, and into 40 years of age or older. The comparison with the cold and warm reference areas without and with stratification on categories of cumulative years of residence yield a similar pattern as in Tables 2 and 3 (detailed results are shown in Tables K, L, and M in S1 File); and the results were similar when applying five-year latency time, shown in Table N in S1 File.

Analysing breast cancer risk with additional adjustment for age at first birth, the HRs in the geothermal heating area in comparison with the warm reference area were 1.17 (95% CI 0.94–1.46), and 1.19 (95% CI 0.96–1.48), without and with stratification on cumulative residence, respectively. The HRs in comparison with cold reference area were 1.37 (1.08–1.74), and 1.43 (1.13–1.82), without and with stratification, respectively. When applying five-year latency time, the HRs in comparison with the warm reference area were 1.07 (0.74–1.54), and 1.12 (0.78–1.61), without and with stratification on cumulative residence respectively; and in comparison with the cold reference area, the HRs were 1.17 (0.79–1.74), and 1.18 (0.79–1.76), without and with stratification, respectively.

Using the relative risk from the study of Thorlacius et al. [32], and the estimated prevalence’s of those with and without the mutation of the BRCA2 gene the risks of female breast cancer were calculated, and are shown in Table O in S1 File. In the population of the geothermal heating area we obtained the value of (7*1.16 + 1*98.84) = 106.96, and for the population of the warm reference area the value was (7*0.58 + 1*99.42) = 103.48, and for the population of the cold reference area the value was (7*0.65 + 1*99.35) = 103.90. The predictive value [29] for the comparison of geothermal heating area versus warm reference area was (106.96/103.48) = 1.03, or 3% increase. The corresponding predictive value for comparison of geothermal heating area versus cold reference area was (106.96/103.90) = 1.03, also 3% increase.

## Discussion

This population-based cohort study with 33 years follow-up with nearly a thousand cancer cases in the geothermal heating area, where geothermal water was used for heating, bathing and washing for decades, showed statistically significant higher risk for all cancers, pancreatic cancer, breast cancer, prostate cancer, kidney cancer, combined cancers of the lymphoid and haematopoietic tissue, NHL, MDS, and BCC of the skin than in the reference areas. The risk for these cancer sites was higher in comparison with the cold reference area than with the warm reference area, through the degree of volcanic/geothermal activity, indicating a dose-response association. When taking cumulative years of residence in the areas into consideration, the risk for these cancer sites were generally higher compared with the risk when length of residence was not accounted for, again in a dose-response manner. In the present study, it was possible to adjust for age, gender, social variables such as education and type of housing, on an individual basis, and for estimates of age at first birth and smoking habits on the community level.

The result of this study with extended follow-up confirms the results from previous similarly designed incidence studies on higher risk for all cancers, pancreatic cancer, breast cancer, prostate cancer, kidney cancer, lymphoid and haematopoietic tissue cancers, NHL, and BCC of the skin [15, 16]. The higher incidence for all cancers and breast cancer in the present study is consistent with the higher incidence of all cancers and breast cancer among the population in the geothermal area than in the non-geothermal area of Furnas, Azores [8], and is also consistent with results of a recently published study indicating higher incidence for all cancers, breast cancer and prostate cancer among the population in Catania, the area with highest volcanic activity compared with the areas with less volcanic activity in Sicily, Italy [17].

A study from Furnas, Azores showed evidence of DNA damage in residents of volcanic active areas in comparison with an area without manifestations of volcanic activity [33].

Recently it has been discussed [34, 35] that disproportional distribution of the mutation of the BRCA2 gene in the geothermal population as compared to the reference populations in previous studies [15, 16] may be a confounding factor, in particular for the association with breast cancer among women [34, 35]. However, this mutation only occurs in 0.6% of the Icelandic population [31] and therefore is not likely to account for our results. Nevertheless, we used the Axelson and Steenland method [29] to estimate the influence of this factor on our findings revealing that although the estimated mutation prevalence is higher in geothermal areas, this increase only account for 3% of the total female breast cancer incidence. Thus, our findings contrasting breast cancer in geothermal areas to warm or cold reference areas yielding HR of 1.27 and 1.48 respectively, are unlikely to be explained solely by confounding due to this mutation. Also, BRCA2 mutation carriers have well documented increased risk of ovarian cancer [36] and the decreased risk of ovarian cancer in geothermal areas in our study further argues against confounding effects of mutation in the BRCA2 gene.

The long follow-up and the number of cancer cases found in the geothermal heating area enabled us to break these into rare subcategories of cancer sites, and it is of interest to observe the details of the 97 cases of combined cancer of the lymphoid and haematopoietic tissue. Several categories of lymphomas and leukaemias had higher HRs, and the 95% CI for all NHL, peripheral T-cell NHL (ICD-10 code C84), and unspecified NHL (ICD-10 code C85) did not include unity. NHL comprises heterogeneous malignancies with regard to clinical, etiological and histological entity [37]. Viral infections, immune deficiencies, and high dose ionizing radiation have been associated with NHL [37], and recent studies support the indications that farming, hairdressing, and textile occupations, red meat and processed meat consumption, and autoimmune conditions may be related to NHL risk [38–40]. The HRs for MDS, based on eight cases, were higher in all analyses, and the 95% CI did not include unity. The cases of MDS were not secondary to cancer treatment, as the study is confined to first cancers only, and these cases were not classified as therapy-related, but had the location code D46.9, MDS, unspecified. All cancers in the ICD-10 category D had morphology behavioural code /3 indicating malignant neoplasm. In another study, familial aggregation was not found in patients with MDS [41], and MDS may arise secondarily after chemotherapy and radiotherapy [42], or exposure to ionizing radiation and benzene [43,44], thus well-known environmental carcinogens.

The causes are unknown for the higher HRs of many cancer sites in the present study, which are related to length of cumulative residence in the study areas. In reflection on this, it is difficult to explain the risk for the different cancer sites by a single component of the ground gas emission in the geothermal area, or traces of chemicals in the geothermal water. When considering the classification of human carcinogens according to the International Agency for Research on Cancer [45], two carcinogens in particular i.e. As and Rn come to mind, as these have been mentioned in previous studies on cancer risk among populations in geothermal areas. A recent mortality study in an old volcanic area provided evidence of association of low dose (below 10 ppb) As in drinking water and cancer risk [46], and in a case-control study a positive association between BCC and a low dose exposure to As was found [47]. The concentrations of As in geothermal well water used for bathing in the geothermal heating area range from 11 to 116 ppb [48], and should be contrasted to < 0.3 ppb in water used for bathing in the cold reference area [49, 50]. According to a recent nation-wide survey of indoor Rn concentration in Iceland, a mean of 13 Bq/m^3^ is among the lowest in the world [51]. Nevertheless the amount of Rn in the geothermal water in the geothermal heating area (9 Bq/l) (used for bathing) [10] is approximately four times the amount of Rn in water used for bathing in the cold reference area (approximately 1.5 Bq/l) [49, 50]. The role of these differences in concentrations is unknown; bearing in mind that dermal exposure may in this situation be of greater importance than exposure through inhalation or ingestion.

Recently, naturally occurring radioactive material (NORM) has been discovered above exemption limit for the first time in Iceland, located in scale precipitated in pipes close to wells (boreholes) in one of the geothermal power plants [52]. The NORM is from the U-238 decay chain and exceeded the exemption limit 10 Bq/g ten to twenty times [52]. At present, more measurements are planned to be undertaken in different power plants. Whether these findings have significance for utilization of the geothermal water in settings other than the power plants, i.e. in dwellings, is still uncertain, as is the possibly far-fetched association with the increased cancer incidence found among the population of the geothermal heating areas in the present study.

### Strength

We count to the strength of the study the long follow-up time of the cohort. Furthermore, the use of the comprehensive population registries and the personal identification number, which enabled easy and accurate record linkage, strengthen the study. Thus, duration of residence, vital, and out-migration status were ascertained through the National Rosters, the National Registry, and the National Cause-of-Death Registry for all individuals in the exposed cohort and the two reference populations, i.e. in the same way for everybody in the study. Information on the outcome, the cancer incidence, was obtained by similarly performed record linkage of every individual of the exposed and the non-exposed populations with the Cancer Registry. 95% of the cancer cases reported to the Cancer Registry are histologically verified, and in the case of BCC, all diagnoses are histologically confirmed.

Screening for breast cancer with mammography has been offered nation-wide to all women 40–69 years of age since 1987, and there are no indications of regional differences in the participation rate [24]. Systematic screening for prostate cancer or skin cancer have not been implemented or recommended in Iceland.

To our best knowledge, the present study is the first to report on cumulative years of residence for every individual in the geothermal heating area (the exposed population) and in the two reference populations, and thus it enables us to take the length of residence, as a surrogate of the exposure to volcanic/geothermal environment, and the use of geothermal water, into consideration in the risk assessments.

### Limitation

Numerous calculations of HRs for all cancer and selected cancer sites were performed in the present study. The HRs for the rare sites of cancers are shown for descriptive purposes. The many calculations performed in the study may give rise to concern regarding multiple comparisons; however, it has been argued that no adjustment is needed for these [53].

The study is limited by the lack of individual exposure information with regard to mode and magnitude of ground gas emission in the geothermal area and the reference areas, and the detailed information on the components of the drinking water, and the geothermal water. However we were able to take length of residence in the areas into consideration as a surrogate of exposure.

The possibility of unknown confounding cannot be excluded, however in the multivariate analysis we were able to adjust for socioeconomic status (level of education, and type of housing), age, and gender on individual level, and smoking habits and age at first birth on community level.

Access to the health care system was found to be easier and the use of the health care system was found to be more frequent, concerning cardiovascular diseases, outside than inside the capital area in Iceland in a recent doctoral thesis [54], however we are not aware of differences in these aspects between the study populations.

In the course of time, geothermal water has become more widely used in Iceland hindering identification of population without the exposure in question. One way to address this problem in future study is to select appropriate reference population from counties without the exposure, in order to increase further the comparability.

In future studies, the data on cumulative years of residence in the respective areas should be used to estimate the long-term exposure to different physical and chemical components occurring in dermal contact with water, in the environment, and in the indoor air, based on historical data, or currently measured concentrations with regard to known carcinogenic factors or mechanisms.

### Conclusion

The significant high cancer risk is consistent with previous findings in the geothermal area and users of geothermal water. Positive dose-response manner of relationship between incidence of cancers and cumulative years of residence, and gradient of geothermal/volcanic activity were shown and need further consideration. Adjustment was made for individual social-related variables, as well as for reproductive factors and smoking habits on the community level. Further studies are needed on the chemical and physical content of the environmental emissions in geothermal areas, and the exposure and the dermal contamination resulting from the use of geothermal water.

## Supporting Information

S1 Fig The log(-log(survival)) versus log(time) curves for exposed group (dashed line) and warm reference group (black line). (TIF)

S2 Fig The log(-log(survival)) versus log(time) curves for exposed group (dashed line) and cold reference group (black line). (TIF)

S1 File Table A. Codes and names of communities in the study populations according to National Registry in 1981. Table B. Baseline characteristics of the study populations, data source were Census 1981^**a**^, Public Health Institute of Iceland^**b**^, Statistics Iceland^**c**^ and National Roasters^**d**^. Table C. Number of all cancers and cancer sites with any case among men and women combined in the geothermal heating areas, hazard ratio (HR), 95% confidence intervals (CI) compared with the populations in warm reference area and cold reference area, adjusted for age, gender, education, type of housing, and smoking habits, without and with stratification into categories of cumulative years of residence in the respective areas. Table D. Number of all cancers and cancer sites with any case among men and women combined in the geothermal heating areas, hazard ratio (HR), 95% confidence intervals (CI) compared with the populations in warm reference area and cold reference area, applying five years latency time, adjusted for age, gender, education, type of housing, and smoking habits, without and with stratification into categories of cumulative years of residence in the respective areas. Table E. Number of all cancers and cancer sites with any case among men in the geothermal heating areas, hazard ratio (HR), 95% confidence intervals (CI) compared with the populations in warm reference area and cold reference area applying five years latency time, adjusted for age, gender, education, type of housing, and smoking habits, without and with stratification into categories of cumulative years of residence in the respective areas. Table F. Number of all cancers and select cancer sites any case among women in the geothermal heating areas, hazard ratio (HR), 95% confidence intervals (CI) compared with the populations in warm reference area and cold reference area applying five years latency time, adjusted for age, gender, education, type of housing, and smoking habits, without and with stratification into categories of cumulative years of residence in the respective areas. Table G. Number of all cancers and cancer sites with any case among men in the geothermal heating areas, hazard ratio (HR), 95% confidence intervals (CI) compared with the populations in warm reference area and cold reference area, adjusted for age, gender, education, type of housing, and smoking habits without and with stratification into categories of cumulative years of residence in the respective areas. Table H. Number of all cancers and cancer sites with any case among women in the geothermal heating areas, hazard ratio (HR), 95% confidence intervals (CI) compared with the populations in warm reference area and cold reference area, adjusted for age, gender, education, type of housing, and smoking habits without and with stratification into categories of cumulative years of residence in the respective areas. Table I. Number of all cancers, and selected cancer sites among men and women combined in the geothermal heating areas, hazard ratio (HR), 95% confidence intervals (CI) compared with the populations in warm reference area and cold reference area, adjusted for age, gender, education, type of housing, and smoking habits, split in four categories of cumulative years of residence in the respective areas. Table J. Number of all cancers, and selected cancer sites among men and women combined in the geothermal heating areas, hazard ratio (HR), 95% confidence intervals (CI) compared with the populations in warm reference area and cold reference area, applying five years latency time, adjusted for age, gender, education, type of housing, and smoking habits, split in four categories of cumulative years of residence in the respective areas. Table K. Number of all cancers, and selected cancer sites among men and women combined, hazard ratio (HR), 95% confidence intervals (CI) compared with the populations in warm reference area and cold reference area, adjusted for age, gender, education, type of housing, and smoking habits, without and with stratification into categories of cumulative years of residence, restricted on different age categories in the respective areas. Table L. Number of all cancers, and selected cancer sites among men in the geothermal heating areas, hazard ratio (HR), 95% confidence intervals (CI) compared with the populations in warm reference area and cold reference area, adjusted for age, gender, education, type of housing, and smoking habits, without and with stratification into categories of cumulative years of residence, restricted on different age categories in the respective areas. Table M. Number of selected cancer sites among women in the geothermal heating areas, hazard ratio (HR), 95% confidence intervals (CI) compared with the populations in warm reference area and cold reference area, adjusted for age, gender, education, type of housing, and smoking habits, without and with stratification into categories of cumulative years of residence, restricted on different age categories in the respective areas. Table N. Number of selected cancer sites among men and women combined in the geothermal heating areas, hazard ratio (HR), 95% confidence intervals (CI) compared with the populations in warm reference area and cold reference area, applying five years latency time, adjusted for age, gender, education, type of housing, and smoking habits, without and with stratification into categories of cumulative years of residence, restricted on different age categories in the respective areas. Table O. Number of individuals, number of male breast cancer cases, number of individuals with and without mutation of the BRCA2 gene, the prevalence of those with, and without the mutation according to Thorlacius et al. [29], and predictive values for breast cancer among females according to the method of Axelson and Steenland [27], and using the relative risk for breast cancer among female according to Thorlacius et al. [30], in the geothermal area in comparison with warm and cold reference areas, and the combined capital area and Reykjanes.
(DOC)
