# Supplementary figures and images for: Association of Cancer Incidence and Duration of Residence in Geothermal Heating Area in Iceland: An Extended Follow-Up
Source: PLoS One. 2016 May 20;11(5):e0155922. doi: 10.1371/journal.pone.0155922 (PMC4874673; doi:10.1371/journal.pone.0155922)

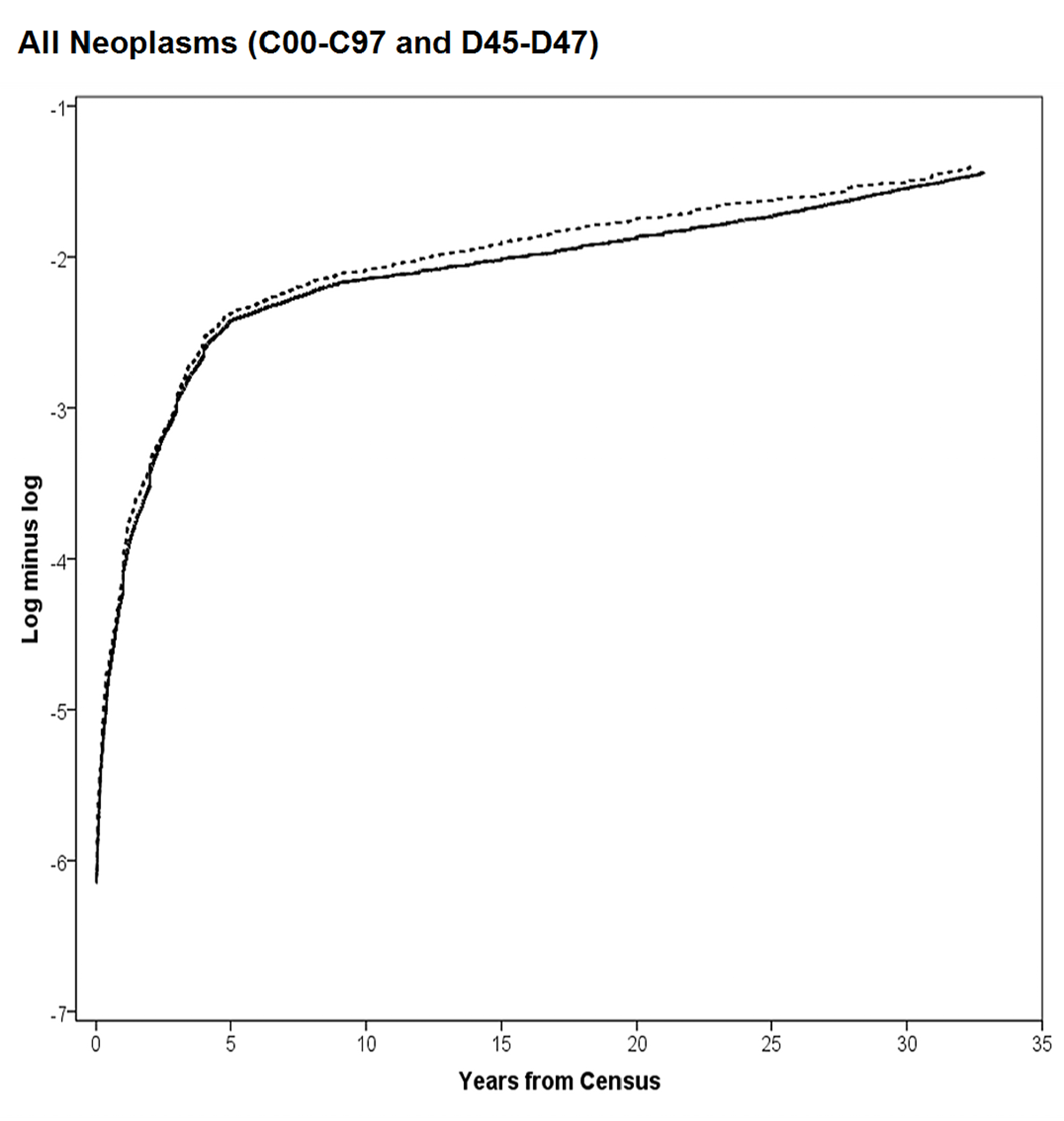

Supplement: S1 Fig — (TIF) [file pone.0155922.s001.tif]

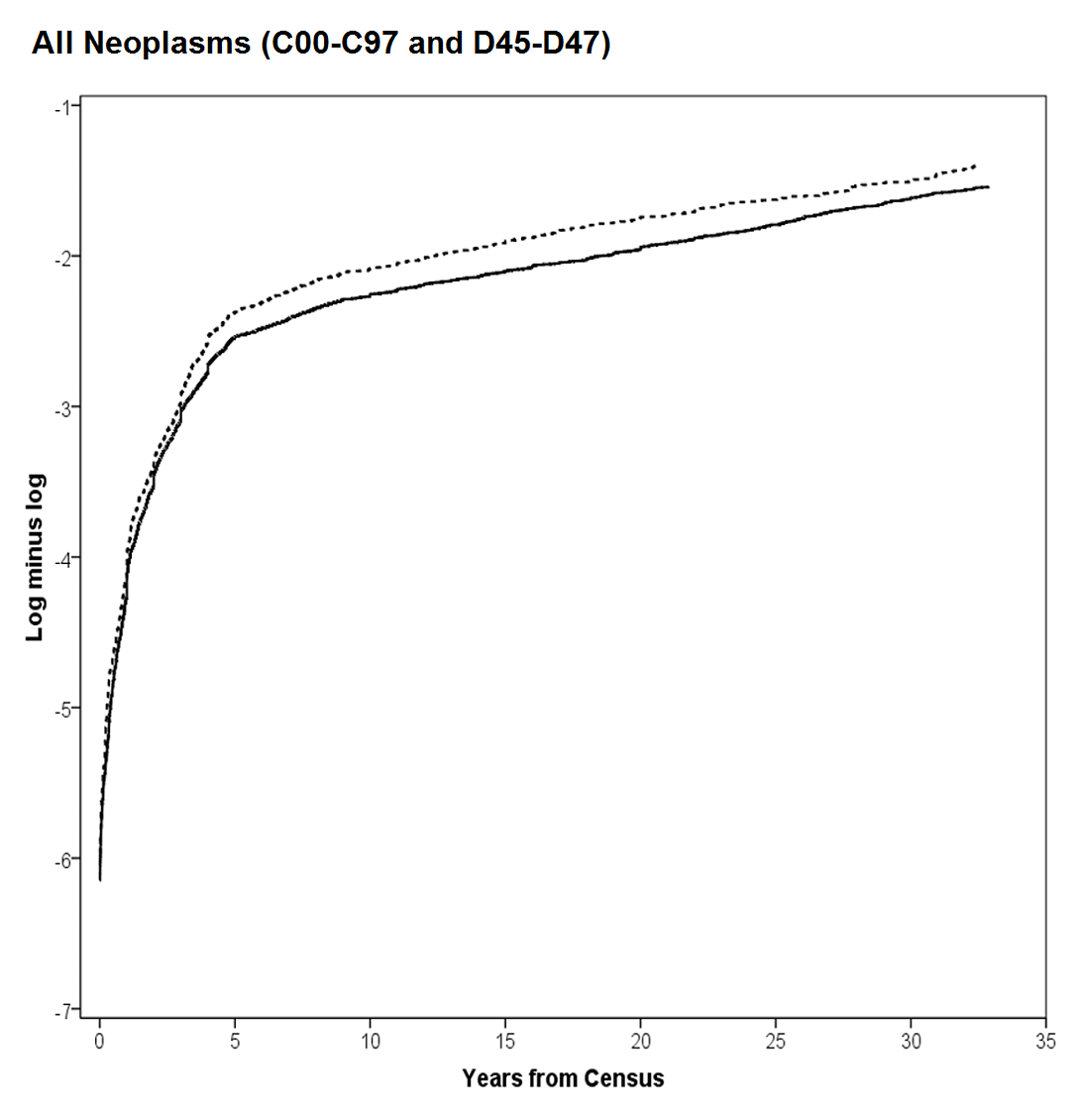

Supplement: S2 Fig — (TIF) [file pone.0155922.s002.tif]
