# Supplementary material for: Association of Cancer Incidence and Duration of Residence in Geothermal Heating Area in Iceland: An Extended Follow-Up
Source: PLoS One. 2016 May 20;11(5):e0155922. doi: 10.1371/journal.pone.0155922 (PMC4874673; doi:10.1371/journal.pone.0155922)
Supplement: S1 File — Table A. Codes and names of communities in the study populations according to National Registry in 1981. Table B. Baseline characteristics of the study populations, data source were Census 1981a, Public Health Institute of Icelandb, Statistics Icelandc and National Roastersd. Table C. Number of all cancers and cancer sites with any case among men and women combined in the geothermal heating areas, hazard ratio (HR), 95% confidence intervals (CI) compared with the populations in warm reference area and cold reference area, adjusted for age, gender, education, type of housing, and smoking habits, without and with stratification into categories of cumulative years of residence in the respective areas. Table D. Number of all cancers and cancer sites with any case among men and women combined in the geothermal heating areas, hazard ratio (HR), 95% confidence intervals (CI) compared with the populations in warm reference area and cold reference area, applying five years latency time, adjusted for age, gender, education, type of housing, and smoking habits, without and with stratification into categories of cumulative years of residence in the respective areas. Table E. Number of all cancers and cancer sites with any case among men in the geothermal heating areas, hazard ratio (HR), 95% confidence intervals (CI) compared with the populations in warm reference area and cold reference area applying five years latency time, adjusted for age, gender, education, type of housing, and smoking habits, without and with stratification into categories of cumulative years of residence in the respective areas. Table F. Number of all cancers and select cancer sites any case among women in the geothermal heating areas, hazard ratio (HR), 95% confidence intervals (CI) compared with the populations in warm reference area and cold reference area applying five years latency time, adjusted for age, gender, education, type of housing, and smoking habits, without and with stratification into ca [file pone.0155922.s003.doc]

Table A. Codes and names of communities in the study populations according to National Registry in 1981.

| **Geothermal heating area** | |  |  |  |  |
| --- | --- | --- | --- | --- | --- |
| 6100 | Husavik ^2^ | 8100 | Selfoss ^2^ | 8711 | Biskupstungnahreppur ^3^ |
| 6607 | Skutustadahreppur ^3^ | 8708 | Skeidahreppur ^3^ | 8712 | Laugardalshreppur ^3^ |
| 6610 | Reykjahreppur ^3^ | 8710 | Hrunamannahreppur^3^ | 8716 | Hveragerdishreppur ^2^ |
|  |  |  |  |  |  |
| **Cold reference area** | |  |  |  |  |
| 3712 | Skogarstrandarhreppur ^3^ | 4805 | Reykjafjardarhreppur ^3^ | 7501 | Skeggjastadahreppur ^3^ |
| 3801 | Hordudalshreppur ^3^ | 4806 | Nauteyrarhreppur ^3^ | 7502 | Vopnafjardarhreppur ^2^ |
| 3802 | Middalshreppur ^3^ | 4807 | Snaefjallahreppur ^3^ | 7503 | Hlidarhreppur ^3^ |
| 3803 | Haukadalshreppur ^3^ | 4901 | Arneshreppur ^3^ | 7504 | Jokuldalshreppur ^3^ |
| 3804 | Laxardalshreppur ^3^ | 4902 | Kaldrananeshreppur ^3^ | 7505 | Fljotsdalshreppur ^3^ |
| 3805 | Hvammshreppur ^3^ | 4903 | Hrofbergshreppur ^3^ | 7506 | Fellahreppur ^3^ |
| 3806 | Fellsstrandarhreppur ^3^ | 4904 | Holmavikurhreppur ^3^ | 7604 | Eidahreppur ^3^ |
| 3807 | Klofningshreppur ^3^ | 4905 | Kirkjubolshreppur ^3^ | 7605 | Mjoafjardarhreppur ^3^ |
| 3808 | Skardshreppur ^3^ | 4906 | Fellahreppur ^3^ | 7606 | Nordfjardarhreppur ^3^ |
| 3809 | Saurbaejarhreppur ^3^ | 4907 | Ospakseyrarhreppur ^3^ | 7607 | Helgustadahreppur ^3^ |
| 4000 | Isafjordur ^2^ | 4908 | Baejarhreppur ^3^ | 7609 | Reydarfjardarhreppur ^2^ |
| 4100 | Bolungarvik ^2^ | 5501 | Stadarhreppur ^3^ | 7610 | Faskrudsfjardarhreppur ^3^ |
| 4501 | Geiradalshreppur ^3^ | 5502 | Fremri-Torfustadahreppur^3^ | 7611 | Budahreppur ^2^ |
| 4502 | Reykholahreppur ^3^ | 5503 | Ytri-Torfustadahreppur ^3^ | 7612 | Stodvarhreppur ^3^ |
| 4503 | Gufudalshreppur ^3^ | 5504 | Hvammstangahreppur ^3^ | 7613 | Breiddalshreppur ^3^ |
| 4504 | Mulahreppur ^3^ | 5505 | Kirkjuhvammshreppur ^3^ | 7614 | Beruneshreppur ^3^ |
| 4505 | Flateyjarhreppur ^3^ | 5506 | Thverarhreppur ^3^ | 7615 | Bulandshreppur ^3^ |
| 4601 | Bardastrandarhreppur ^3^ | 5507 | Thorkelsholahreppur ^3^ | 7616 | Geithellnahreppur ^3^ |
| 4602 | Raudasandshreppur ^3^ | 5601 | Ashreppur ^3^ | 7701 | Baejarhreppur ^3^ |
| 4603 | Patrekshreppur ^2^ | 5602 | Sveinsstadahreppur ^3^ | 7702 | Nesjahreppur ^3^ |
| 4604 | Talknafjardarhreppur ^3^ | 5603 | Torfalaekjarhreppur ^3^ | 7703 | Hafnarhreppur ^2^ |
| 4605 | Ketildalshreppur ^3^ | 5604 | Blonduoshreppur ^2^ | 7704 | Myrahreppur ^3^ |
| 4606 | Sudurfjardahreppur ^3^ | 5605 | Svinavatnshreppur ^3^ | 7705 | Borgarhafnarhreppur ^3^ |
| 4701 | Audkuluhreppur ^3^ | 5606 | Bolstadahlidarhreppur ^3^ | 7706 | Hofshreppur ^3^ |
| 4702 | Thingeyjarhreppur ^3^ | 5607 | Engihlidarhreppur ^3^ | 7507 | Tunguhreppur ^3^ |
| 4703 | Myrahreppur ^3^ | 5608 | Vindhaelishreppur ^3^ | 7508 | Hjaltastadarhreppur ^3^ |
| 4704 | Mosfellshreppur ^3^ | 5609 | Hofdahreppur ^2^ | 7509 | Borgarfjardarhreppur ^3^ |
| 4705 | Flateyrarhreppur ^3^ | 5610 | Skagahreppur ^3^ | 7511 | Seydisfjardarhreppur ^3^ |
| 4706 | Sudureyrarhreppur ^3^ | 7000 | Seydisfjordur ^2^ | 7601 | Skriddalshreppur ^3^ |
| 4803 | Sudavikurhreppur ^3^ | 7100 | Neskaupstadur ^2^ | 7602 | Vallahreppur ^3^ |
| 4804 | Ogurhreppur ^3^ | 7200 | Eskifjordur ^2^ | 7603 | Egilsstadahreppur ^2^ |
|  |  |  |  |  |  |
| **Warm reference area** | |  |  |  |  |
| 2603 | Kjalarnes ^3^ | 5704 | Seiluhreppur ^3^ | 6704 | Prestholahreppur ^3^ |
| 2604 | Kjosarhreppur ^3^ | 5705 | Lytingsstadahreppur ^3^ | 6705 | Raufarhafnarhreppur ^3^ |
| 3000 | Akranes ^2^ | 5706 | Akrahreppur ^3^ | 6706 | Svalbardshreppur ^3^ |
| 3501 | Strandarhreppur ^3^ | 5707 | Ripurhreppur ^3^ | 6707 | Thorshafnarhreppur ^3^ |
| 3502 | Skilmannahreppur ^3^ | 5708 | Vidvikurhreppur ^3^ | 6708 | Saudaneshreppur ^3^ |
| 3503 | Innri-Akraneshreppur ^3^ | 5709 | Holahreppur ^3^ | 8000 | Vestmannaeyjar ^2^ |
| 3504 | Leirar- og Melahreppur ^3^ | 5710 | Hofshreppur ^3^ | 8501 | Horgslandshreppur ^3^ |
| 3505 | Andakilshreppur ^3^ | 5711 | Hofsoshreppur ^3^ | 8502 | Kirkjubaejarhreppur ^3^ |
| 3506 | Skorradalshreppur ^3^ | 5712 | Fellshreppur ^3^ | 8503 | Skaftartunguhreppur ^3^ |
| 3507 | Lundarreykjadalshreppur ^3^ | 5713 | Haganeshreppur ^3^ | 8504 | Leidvallarhreppur ^3^ |
| 3508 | Reykholtsdalshreppur ^3^ | 5714 | Holtshreppur ^3^ | 8505 | Alftavershreppur ^3^ |
| 3509 | Halsahreppur ^3^ | 6000 | Akureyri ^2^ | 8506 | Hvammshreppur ^3^ |
| 3601 | Hvitarsiduhreppur ^3^ | 6200 | Olafsfjordur ^2^ | 8507 | Dyrholahreppur ^3^ |
| 3602 | Thverarhlidarhreppur ^3^ | 6300 | Dalvik ^2^ | 8601 | A-Eyjafjallahreppur ^3^ |
| 3603 | Nordurardalshreppur ^3^ | 6501 | Grimseyjarhreppur ^3^ | 8602 | V-Eyjafjallahreppur ^3^ |
| 3604 | Stafholtstungnahreppur^3^ | 6502 | Svarfadardalshreppur ^3^ | 8603 | A-Landeyjahreppur ^3^ |
| 3605 | Borgarhreppur ^3^ | 6504 | Hriseyjarhreppur ^3^ | 8604 | V-Landeyjahreppur ^3^ |
| 3606 | Borgarneshreppur ^2^ | 6505 | Arskogshreppur ^3^ | 8605 | Fljotshlidarhreppur ^3^ |
| 3607 | Alftaneshreppur ^3^ | 6506 | Arnarneshreppur ^3^ | 8606 | Hvolhreppur ^2^ |
| 3608 | Hraunhreppur ^3^ | 6507 | Skriduhreppur ^3^ | 8607 | Rangarvallahreppur ^2^ |
| 3701 | Kolbeinsstadahreppur ^3^ | 6508 | Oxnadalshreppur ^3^ | 8608 | Landmannahreppur ^3^ |
| 3702 | Eyjahreppur ^3^ | 6509 | Glaesibaejarhreppur ^3^ | 8609 | Holtahreppur ^3^ |
| 3703 | Miklaholtshreppur ^3^ | 6510 | Hrafnagilshreppur ^3^ | 8610 | Asahreppur ^3^ |
| 3704 | Stadarsveit ^3^ | 6511 | Saurbaejarhreppur ^3^ | 8611 | Djuparhreppur ^3^ |
| 3705 | Breiduvikurhreppur ^3^ | 6512 | Ongulsstadahreppur ^3^ | 8701 | Gaulverjabaejarhreppur ^3^ |
| 3706 | Neshreppur ^2^ | 6601 | Svalbardsstrandarhreppur ^3^ | 8702 | Stokkseyrarhreppur ^3^ |
| 3707 | Olafsvikurhreppur ^2^ | 6602 | Grytubakkahreppur ^3^ | 8703 | Eyrarbakkahreppur ^3^ |
| 3708 | Frodarhreppur ^3^ | 6604 | Halshreppur ^3^ | 8704 | Sandvikurhreppur ^3^ |
| 3709 | Eyrarsveit ^2^ | 6605 | Ljosavatnshreppur ^3^ | 8706 | Hraungerdishreppur ^3^ |
| 3710 | Helgafellssveit ^3^ | 6606 | Barddaelahreppur ^3^ | 8707 | Villingaholtshreppur ^3^ |
| 3711 | Stykkisholmshreppur ^2^ | 6608 | Reykdaelahreppur ^3^ | 8709 | Gnupverjahreppur ^3^ |
| 5000 | Siglufjordur ^2^ | 6609 | Adaldaelahreppur ^3^ | 8713 | Grimsneshreppur ^3^ |
| 5100 | Saudarkrokur ^2^ | 6611 | Tjorneshreppur ^3^ | 8714 | Thingvallahreppur ^3^ |
| 5701 | Skefilsstadahreppur ^3^ | 6701 | Kelduneshreppur ^3^ | 8715 | Grafningshreppur ^3^ |
| 5702 | Skardshreppur ^3^ | 6702 | Oxarfjardarhreppur ^3^ | 8717 | Olfushreppur ^2^ |
| 5703 | Stadarhreppur ^3^ | 6703 | Fjallahreppur ^3^ | 8718 | Selvogshreppur ^3^ |
|  |  |  |  |  |  |
| **Capital area, and south-west peninsula of Reykjanes** | |  |  |  |  |
| 0000 | Reykjavik ^1^ | 1603 | Bessastadahreppur ^3^ | 2502 | Hafnarhreppur ^3^ |
| 1000 | Kopavogur ^2^ | 1604 | Mosfellshreppur ^2^ | 2503 | Midneshreppur ^2^ |
| 1100 | Seltjarnarnes ^2^ | 2200 | Keflavik ^2^ | 2504 | Gerdahreppur ^2^ |
| 1300 | Gardabaer ^2^ | 2300 | Grindavik ^2^ | 2506 | Vatnsleysustrandahreppur^3^ |
| 1400 | Hafnarfjordur ^2^ | 2400 | Njardvik ^2^ |  |  |

^1^ The capital, n = 65.961, ^2^ Other urban regions, n ≥ 500 and ≤ 12.195, ^3^ Rural regions, n < 500.

Table B. Baseline characteristics of the study populations, data source were Census 1981**^a^**, Public Health Institute of Iceland**^b^**, Statistics Iceland**^c^** and National Roasters**^d^**.

|  | Geothermal heating area | Warm reference area | Cold reference area |
| --- | --- | --- | --- |
|  | N (%) | N (%) | N (%) |
| **Number of people^a^** | 7511 (100) | 44 864 (100) | 22 431 (100) |
| **Gender^a^** |  |  |  |
| Men | 3893 (51.8) | 23 305 (51.9) | 11 929 (53.2) |
| Woman | 3618 (48.2) | 21 559 (48.1) | 10 502 (46.8) |
| **Age, year^a^** |  |  |  |
| Mean ± SD | 28.81 ± 16.35 | 28.65 ± 16.30 | 28.56 ± 16.20 |
| Median, IQR (0.25 ; 0.75) | 26 (15 ; 41) | 26 (15 ; 40) | 26 (15 ; 40) |
| **Education^a^** |  |  |  |
| Basic education | 1829 (24.3) | 12 370 (27.6) | 6565 (29.3) |
| Medium education | 2088 (27.8) | 11 754 (26.2) | 5665 (25.3) |
| Academic education | 622 (8.3) | 3171 (7.1) | 1428 (6.4) |
| Unclassified | 2878 (38.3) | 17 061 (38.0) | 8481 (37.7) |
| Missing | 94 (1.3) | 508 (1.1) | 292 (1.3) |
| **Housing^a^** |  |  |  |
| Single family home | 5730 (76.3) | 29 236 (65.2) | 17 343 (77.3) |
| Other type of house | 1781 (23.7) | 15 628 (34.8) | 5088 (22.7) |
| **Region^a^** |  |  |  |
| Urban regions ≥ 500 individuals | 5793 (77.1) | 30 554 (68.1) | 12 839 (57.2) |
| Rural regions < 500 individuals | 1718 (22.9) | 14 310 (31.9) | 9592 (42.8) |
| **Never smoker %^b^** |  |  |  |
| Mean ± SD | 43.8 ± 4.7 | 46.9 ± 5.7 | 44.7 ± 8.0 |
| Median, IQR (0.25 ; 0.75) | 43.3 (40 ; 45) | 46.7 (44 ; 50) | 46.5 (42 ; 49) |
| **Proportion giving birth before 25 years of age^c^** |  |  |  |
| Mean ± SD | 4.25 ± 2.03 | 2.18 ± 0.65 | 2.17 ± 0.78 |
| Median, IQR (0.25 ; 0.75) | 3.42 (3.08 ; 6.67) | 2.18 (1.73 ; 2.28) | 2.48 (1.27 ; 2.84) |
| **Numbers in categories of cumulative years of residence^ad^** |  |  |  |
| < 5 years | 1479 (19.7) | 6935 (15.5) | 4029 (18.0) |
| ≥ 5 to <10 years | 1061 (14.1) | 5808 (12.9) | 3213 (14.3) |
| ≥ 10 to < 15 years | 843 (11.2) | 5688 (12.7) | 2853 (12.7) |
| ≥ 15 to 20 years | 671 (8.9) | 3829 (8.5) | 1880 (8.4) |
| ≥ 20 to 24 years | 492 (6.6) | 3235 (7.2) | 1861 (8.3) |
| ≥ 24 years | 2965 (39.5) | 19 369 (43.2) | 8595 (38.3) |
| **Age (years) at census in categories of cumulative years of residence^ad^** |  |  |  |
| **< 5 years** |  |  |  |
| Mean ± SD | 26.83 ± 14.78 | 26.71 ± 14.93 | 26.16 ± 14.54 |
| Median, IQR (0.25 ; 0.75) | 23 (17 ; 34) | 24 (17 ; 34) | 24 (16 ; 33) |
| **≥ 5 to <10 years** |  |  |  |
| Mean ± SD | 24.67 ± 15.86 | 25.09 ± 15.66 | 25.39 ± 15.77 |
| Median, IQR (0.25 ; 0.75) | 19 (14 ; 33) | 20 (14 ; 34) | 20 (14 ; 33) |
| **≥ 10 to < 15 years** |  |  |  |
| Mean ± SD | 25.48 ± 17.09 | 26.92 ± 17.25 | 27.02 ± 17.56 |
| Median, IQR (0.25 ; 0.75) | 18 (12 ; 36) | 20 (13 ; 39) | 20 (13 ; 40) |
| **≥ 15 to 20 years** |  |  |  |
| Mean ± SD | 27.93 ± 20.24 | 27.88 ± 19.86 | 26.96 ± 19.20 |
| Median, IQR (0.25 ; 0.75) | 19 (9 ; 48) | 22 (9 ; 47) | 21 (10 ; 44) |
| **≥ 20 to 24 years** |  |  |  |
| Mean ± SD | 31.75 ± 18.79 | 31.07 ± 19.14 | 30.24 ± 18.28 |
| Median, IQR (0.25 ; 0.75) | 32 (13 ; 49) | 30 (12 ; 49) | 28 (13 ; 46) |
| **≥ 24 years** |  |  |  |
| Mean ± SD | 31.95 ± 14.88 | 30.66 ± 14.97 | 31.38 ± 14.86 |
| Median, IQR (0.25 ; 0.75) | 31 (20 ; 43) | 30 (19 ; 42) | 30 (20 ; 42) |

Table C. Number of all cancers and cancer sites with any case among men and women combined in the geothermal heating areas, hazard ratio (HR), 95% confidence intervals (CI) compared with the populations in warm reference area and cold reference area, adjusted for age, gender, education, type of housing, and smoking habits, without and with stratification into categories of cumulative years of residence in the respective areas.

|  | Geothermal heating area |  | Warm reference area | | | | |  | | Cold reference area | | | | | |  | |
| --- | --- | --- | --- | --- | --- | --- | --- | --- | --- | --- | --- | --- | --- | --- | --- | --- | --- |
|  | p-yr 97 911 |  | p-yr 557 815 | | | | |  | | p-yr 299 878 | | | | | |  | |
|  | No of |  | No of | Not stratified | | Stratified | | |  | | No of | Not stratified | | Stratified | | |  |
| Cancers (ICD-10) | cancers |  | cancers | HR | 95%CI | HR | 95%CI | |  | | cancers | HR | 95%CI | HR | 95%CI | |  |
| All (C00-C97, D45-D47) | 988 |  | 5331 | 1.06 | 0.99 to 1.14 | **1.10** | **1.02 to 1.18** | |  | | 2524 | **1.17** | **1.09 to 1.26** | **1.21** | **1.12 to 1.30** | |  |
| Lip, oral cavity, and pharynx (C00-C14) | 16 |  | 103 | 0.79 | 0.46 to 1.36 | 0.80 | 0.47 to 1.38 | |  | | 41 | 1.19 | 0.67 to 2.14 | 1.23 | 0.69 to 2.22 | |  |
| Oesophagus (C15) | 13 |  | 72 | 0.95 | 0.52 to 1.75 | 0.99 | 0.54 to 1.83 | |  | | 36 | 1.12 | 0.59 to 2.12 | 1.13 | 0.59 to 2.14 | |  |
| Stomach (C16) | 30 |  | 196 | 0.87 | 0.58 to 1.28 | 0.88 | 0.59 to 1.30 | |  | | 114 | 0.80 | 0.53 to 1.19 | 0.81 | 0.54 to 1.21 | |  |
| Small intestine (C17) | 4 |  | 25 | 0.87 | 0.30 to 2.59 | 0.92 | 0.31 to 2.73 | |  | | 13 | 1.36 | 0.43 to 4.37 | 1.53 | 0.47 to 4.98 | |  |
| Colon, rectum, and anus (C18-C21) | 90 |  | 473 | 1.11 | 0.88 to 1.41 | 1.16 | 0.92 to 1.47 | |  | | 219 | 1.21 | 0.95 to 1.55 | 1.24 | 0.97 to 1.59 | |  |
| Bile and liver (C22-C24) | 10 |  | 79 | 0.71 | 0.36 to 1.40 | 0.74 | 0.37 to 1.45 | |  | | 34 | 0.90 | 0.44 to 1.84 | 0.93 | 0.45 to 1.90 | |  |
| Pancreas (C25) | 30 |  | 126 | 1.50 | 0.99 to 2.27 | **1.53** | **1.00 to 2.32** | |  | | 49 | **1.87** | **1.18 to 2.95** | **1.93** | **1.22 to 3.06** | |  |
| Nasal cavity and middle ear (C30) | 1 |  | 9 | 0.83 | 0.10 to 7.11 | 0.83 | 0.10 to 7.13 | |  | | 5 | 0.57 | 0.07 to 4.93 | 0.61 | 0.07 to 5.30 | |  |
| Larynx (C32) | 4 |  | 32 | 0.76 | 0.26 to 2.21 | 0.75 | 0.25 to 2.19 | |  | | 14 | 0.90 | 0.29 to 2.74 | 0.90 | 0.29 to 2.76 | |  |
| Lung and bronchus (C33-C34) | 92 |  | 578 | 0.91 | 0.72 to 1.14 | 0.94 | 0.75 to 1.18 | |  | | 300 | 0.92 | 0.73 to 1.17 | 0.96 | 0.76 to 1.22 | |  |
| Bone (C40-C41) | 4 |  | 21 | 1.05 | 0.35 to 3.18 | 0.98 | 0.33 to 2.96 | |  | | 6 | 1.93 | 0.54 to 6.87 | 2.00 | 0.56 to 7.12 | |  |
| Melanoma (C43) | 24 |  | 138 | 0.93 | 0.60 to 1.46 | 0.99 | 0.63 to 1.55 | |  | | 57 | 1.27 | 0.79 to 2.05 | 1.32 | 0.81 to 2.13 | |  |
| Other cancer of skin (C44) | 26 |  | 184 | 0.80 | 0.52 to 1.22 | 0.83 | 0.54 to 1.27 | |  | | 88 | 0.88 | 0.57 to 1.36 | 0.89 | 0.57 to 1.38 | |  |
| Kaposi´s sarcoma (C46) | 1 |  | 10 | 0.56 | 0.07 to 4.68 | 0.60 | 0.07 to 5.09 | |  | | 7 | 0.40 | 0.05 to 3.30 | 0.45 | 0.54 to 3.74 | |  |
| Peritoneum (C48) | 2 |  | 15 | 0.61 | 0.14 to 2.72 | 0.60 | 0.14 to 2.69 | |  | | 7 | 0.89 | 0.19 to 4.31 | 0.93 | 0.19 to 4.53 | |  |
| Soft tissue sarcoma (C49) | 6 |  | 37 | 0.91 | 0.37 to 2.21 | 0.95 | 0.39 to 2.31 | |  | | 15 | 1.18 | 0.45 to 3.04 | 1.26 | 0.48 to 3.26 | |  |
| Breast (C50) | 161 |  | 740 | **1.23** | **1.03 to 1.46** | **1.27** | **1.07 to 1.52** | |  | | 326 | **1.42** | **1.18 to 1.72** | **1.48** | **1.23 to 1.80** | |  |
| Vulva (C51) | 3 |  | 9 | 1.75 | 0.45 to 6.78 | 1.69 | 0.43 to 6.65 | |  | | 5 | 1.88 | 0.44 to 7.95 | 2.17 | 0.51 to 9.31 | |  |
| Vagina (C52) | 1 |  | 2 | 3.08 | 0.23 to 41.26 | 3.33 | 0.24 to 46.49 | |  | | 3 | 0.91 | 0.09 to 8.84 | 1.36 | 0.13 to 13.87 | |  |
| Cervix uteri (C53) | 14 |  | 81 | 0.93 | 0.52 to 1.67 | 0.90 | 0.51 to 1.62 | |  | | 31 | 1.33 | 0.71 to 2.51 | 1.36 | 0.72 to 2.56 | |  |
| Uterus (C54-C55) | 22 |  | 115 | 1.14 | 0.71 to 1.84 | 1.22 | 0.76 to 1.97 | |  | | 59 | 1.07 | 0.65 to 1.74 | 1.12 | 0.68 to 1.83 | |  |
| Ovary (C56-C57) | 20 |  | 145 | 0.83 | 0.52 to 1.35 | 0.88 | 0.54 to 1.43 | |  | | 64 | 0.91 | 0.55 to 1.50 | 0.97 | 0.58 to 1.60 | |  |
| Penis (C60) | 2 |  | 11 | 1.00 | 0.21 to 4.79 | 1.11 | 0.23 to 5.36 | |  | | 3 | 2.14 | 0.32 to 14.22 | 1.90 | 0.28 to 12.95 | |  |
| Prostate (C61) | 172 |  | 803 | **1.27** | **1.07 to 1.51** | **1.32** | **1.11 to 1.57** | |  | | 377 | **1.43** | **1.19 to 1.72** | **1.47** | **1.22 to 1.77** | |  |
| Testis (C62) | 2 |  | 42 | 0.30 | 0.07 to 1.25 | 0.28 | 0.07 to 1.19 | |  | | 23 | 0.28 | 0.07 to 1.17 | 0.29 | 0.07 to 1.22 | |  |
| Kidney (C64-C66) | 49 |  | 241 | 1.21 | 0.88 to 1.67 | 1.27 | 0.92 to 1.75 | |  | | 103 | **1.40** | **1.00 to 1.97** | **1.46** | **1.03 to 2.05** | |  |
| Bladder (C67) | 44 |  | 242 | 0.97 | 0.70 to 1.35 | 1.00 | 0.72 to 1.40 | |  | | 137 | 0.96 | 0.69 to 1.36 | 1.01 | 0.72 to 1.42 | |  |
| Urinary organs, unspecified (C68) | 1 |  | 2 | 2.68 | 0.21 to 33.75 | 2.22 | 0.17 to 29.77 | |  | | 1 | 3.36 | 0.20 to 55.46 | 3.54 | 0.22 to 57.47 | |  |
| Brain and central nervous system (C70-C72, C75.1 and C75.3) | 11 |  | 99 | 0.68 | 0.36 to 1.29 | 0.68 | 0.36 to 1.30 | |  | | 41 | 0.87 | 0.44 to 1.71 | 0.87 | 0.44 to 1.72 | |  |
| Thyroid gland (C73) | 25 |  | 131 | 1.16 | 0.75 to 1.82 | 1.21 | 0.77 to 1.89 | |  | | 68 | 1.10 | 0.70 to 1.75 | 1.16 | 0.73 to 1.84 | |  |
| Cancer without specification of site (C80) | 11 |  | 114 | 0.61 | 0.32 to 1.14 | 0.63 | 0.33 to 1.18 | |  | | 57 | 0.60 | 0.31 to 1.14 | 0.62 | 0.33 to 1.19 | |  |
| Lymphoid and haematopoietic tissue (LH) (C81-C96, D45-D47) | 97 |  | 419 | **1.30** | **1.03 to 1.64** | **1.36** | **1.08 to 1.72** | |  | | 199 | **1.49** | **1.17 to 1.91** | **1.54** | **1.21 to 1.97** | |  |
| Hodgkin´s lymphoma (C81) | 6 |  | 34 | 0.89 | 0.37 to 2.17 | 0.90 | 0.37 to 2.21 | |  | | 14 | 1.50 | 0.55 to 4.03 | 1.49 | 0.55 to 4.02 | |  |
| Non-Hodgkin´s lymphoma (NHL) (C82-C85) | 39 |  | 137 | **1.78** | **1.22 to 2.59** | **1.90** | **1.30 to 2.77** | |  | | 62 | **2.00** | **1.33 to 3.03** | **2.08** | **1.38 to 3.15** | |  |
| NHL, follicular (C82) | 7 |  | 30 | 1.21 | 0.52 to 2.82 | 1.34 | 0.57 to 3.16 | |  | | 13 | 1.76 | 0.70 to 4.44 | 1.80 | 0.71 to 4.56 | |  |
| NHL, diffuse (C83) | 17 |  | 82 | 1.38 | 0.80 to 2.39 | 1.48 | 0.85 to 2.57 | |  | | 40 | 1.38 | 0.77 to 2.48 | 1.45 | 0.80 to 2.62 | |  |
| NHL, peripheral T-cells (C84) | 6 |  | 14 | **2.85** | **1.02 to 7.99 ^b^** | **2.91** | **1.04 to 8.19 ^b^** | |  | | 5 | **3.84** | **1.11 to 13.31 ^b^** | **3.93** | **1.12 to 13.74 ^a^** | |  |
| NHL, unspecified (C85) | 9 |  | 11 | **5.13** | **1.99 to 13.21 ^a^** | **5.23** | **2.02 to 13.54 ^a^** | |  | | 4 | **6.81** | **2.03 to 22.80 ^a^** | **6.75** | **2.01 to 22.64 ^a^** | |  |
| Immunoproliferative diseases (C88) | 6 |  | 25 | 1.35 | 0.53 to 3.40 | 1.51 | 0.59 to 3.85 | |  | | 12 | 1.48 | 0.56 to 3.96 | 1.68 | 0.62 to 4.53 | |  |
| Multiple Myeloma (C90) | 16 |  | 76 | 1.16 | 0.66 to 2.02 | 1.20 | 0.68 to 2.10 | |  | | 39 | 1.22 | 0.68 to 2.18 | 1.21 | 0.68 to 2.18 | |  |
| Leukaemia (C91-C95, D45-D47) | 30 |  | 145 | 1.10 | 0.73 to 1.65 | 1.13 | 0.75 to 1.70 | |  | | 71 | 1.30 | 0.85 to 2.00 | 1.37 | 0.89 to 2.11 | |  |
| Non-CLL (C91-C95, D45-D47, except C91.1) | 20 |  | 100 | 1.07 | 0.65 to 1.76 | 1.09 | 0.67 to 1.80 | |  | | 47 | 1.31 | 0.77 to 2.22 | 1.35 | 0.80 to 2.29 | |  |
| Lymphoid leukemia (C91) | 12 |  | 62 | 0.98 | 0.52 to 1.84 | 1.00 | 0.53 to 1.90 | |  | | 28 | 1.35 | 0.68 to 2.67 | 1.45 | 0.72 to 2.89 | |  |
| Acute lymphoid leukemia (C91.0) | 1 |  | 12 | 0.49 | 0.06 to 3.96 | 0.48 | 0.06 to 3.84 | |  | | 2 | 2.34 | 0.17 to 32.47 | 2.45 | 0.17 to 34.95 | |  |
| Chronic lymphocytic leukaemia (CLL)(C91.1) | 10 |  | 45 | 1.16 | 0.57 to 2.35 | 1.22 | 0.59 to 2.49 | |  | | 24 | 1.30 | 0.62 to 2.73 | 1.45 | 0.68 to 3.08 | |  |
| Other lymphoid leukemia (C91.2 to C91.9) | 1 |  | 5 | 0.89 | 0.10 to 7.82 | 0.90 | 0.10 to 7.95 | |  | | 2 | 1.42 | 0.11 to 17.71 | 1.12 | 0.09 to 14.61 | |  |
| Myeloid leukemia (C92) | 6 |  | 38 | 0.92 | 0.38 to 2.22 | 0.89 | 0.37 to 2.17 | |  | | 27 | 0.72 | 0.30 to 1.75 | 0.76 | 0.31 to 1.86 | |  |
| Acute myeloid leukemia (C92.0) | 4 |  | 26 | 0.94 | 0.32 to 2.80 | 0.94 | 0.32 to 2.79 | |  | | 20 | 0.61 | 0.21 to 1.80 | 0.63 | 0.21 to 1.86 | |  |
| Other myeloid leukemia (C92.1 to C92.9) | 2 |  | 12 | 0.88 | 0.19 to 4.04 | 0.83 | 0.18 to 3.81 | |  | | 7 | 1.14 | 0.23 to 5.57 | 1.32 | 0.27 to 6.53 | |  |
| Other and unspecified leukemia (C93 to C95) | 1 |  | 7 | 0.65 | 0.08 to 5.44 | 0.67 | 0.08 to 5.70 | |  | | 0 |  |  |  |  | |  |
| Myelodysplastic syndromes (MDS) (D46) | 8 |  | 18 | 2.31 | 0.95 to 5.58 | **2.44** | **1.01 to 5.90 ^b^** | |  | | 6 | **4.02** | **1.38 to 11.71 ^a^** | **4.07** | **1.39 to 11.93 ^a^** | |  |
| MDS, unspecified (D46.9) | 8 |  | 12 | **3.41** | **1.32 to 8.83 ^a^** | **3.70** | **1.42 to 9.64 ^a^** | |  | | 3 | **8.20** | **2.10 to 32.10 ^a^** | **8.09** | **2.06 to 31.79 ^a^** | |  |
| Other LH, uncertain (D47) | 3 |  | 14 | 1.24 | 0.34 to 4.54 | 1.41 | 0.38 to 5.21 | |  | | 7 | 1.21 | 0.31 to 4.70 | 1.14 | 0.29 to 4.44 | |  |
| Other LH thrombocythemia (D47.3) | 3 |  | 2 | **11.43** | **1.64 to 79.80 _b_** | **12.72** | **1.80 to 89.74 ^b^** | |  | | 1 | 8.61 | 0.85 to 86.98 | 7.53 | 0.74 to 76.48 | |  |
| **Not included in all cancers** | | | | | | | | | | | | | | | |  | |
|  | p-yr 101 014 |  | p-yr 574 065 | | | | |  | | p-yr 307 763 | | | | | | | |
| Basal cell carcinoma of the skin (BCC) (C44) | 177 |  | 781 | **1.22** | **1.03 to 1.45** | **1.28** | **1.08 to 1.52** | |  | | 335 | **1.54** | **1.28 to 1.85** | **1.62** | **1.35 to 1.94** | |  |

Abbreviation: p-yr, person years.

**^a^** 95% CI computed with bootstrap method did not include unity. **^b^** 95% CI computed with bootstrap method included unity.

Table D. Number of all cancers and cancer sites with any case among men and women combined in the geothermal heating areas, hazard ratio (HR), 95% confidence intervals (CI) compared with the populations in warm reference area and cold reference area, applying five years latency time, adjusted for age, gender, education, type of housing, and smoking habits, without and with stratification into categories of cumulative years of residence in the respective areas.

|  | Geothermal heating area |  | Warm reference area | | | | |  | | Cold reference area | | | | | | | |
| --- | --- | --- | --- | --- | --- | --- | --- | --- | --- | --- | --- | --- | --- | --- | --- | --- | --- |
|  | p-yr 65 169 |  | p-yr 362 298 | | | | |  | | p-yr 201 705 | | | | | | | |
|  | No of |  | No of | Not stratified | | Stratified | | |  | | No of | | Not stratified | | Stratified | | |
| Cancers (ICD-10) | cancers |  | cancers | HR | 95%CI | HR | 95%CI | |  | | cancers | | HR | 95%CI | HR | 95%CI | |
| All (C00-C97, D45-D47) | 372 |  | 1845 | 1.11 | 0.99 to 1.25 | **1.16** | **1.03 to 1.30** | |  | | 959 | | **1.19** | **1.06 to 1.35** | **1.22** | **1.08 to 1.37** | |
| Lip, oral cavity, and pharynx (C00-C14) | 3 |  | 37 | 0.40 | 0.12 to 1.32 | 0.43 | 0.13 to 1.40 | |  | | 12 | | 0.90 | 0.24 to 3.37 | 0.93 | 0.25 to 3.52 | |
| Oesophagus (C15) | 5 |  | 35 | 0.82 | 0.31 to 2.15 | 0.84 | 0.32 to 2.23 | |  | | 16 | | 1.05 | 0.38 to 2.89 | 1.09 | 0.39 to 3.01 | |
| Stomach (C16) | 9 |  | 49 | 0.96 | 0.46 to 2.00 | 1.03 | 0.49 to 2.14 | |  | | 29 | | 0.98 | 0.46 to 2.08 | 1.02 | 0.48 to 2.16 | |
| Small intestine (C17) | 1 |  | 10 | 0.58 | 0.07 to 4.82 | 0.56 | 0.07 to 4.62 | |  | | 8 | | 0.63 | 0.07 to 5.48 | 0.70 | 0.08 to 6.16 | |
| Colon, rectum, and anus (C18-C21) | 32 |  | 163 | 1.09 | 0.74 to 1.62 | 1.16 | 0.78 to 1.72 | |  | | 78 | | 1.27 | 0.84 to 1.92 | 1.29 | 0.85 to 1.96 | |
| Bile and liver (C22-C24) | 4 |  | 22 | 1.05 | 0.35 to 3.18 | 1.14 | 0.37 to 3.47 | |  | | 7 | | 1.77 | 0.52 to 6.10 | 1.82 | 0.53 to 6.30 | |
| Pancreas (C25) | 11 |  | 36 | 1.92 | 0.94 to 3.91 | **2.11** | **1.03 to 4.34** | |  | | 19 | | 1.90 | 0.90 to 4.00 | 2.01 | 0.95 to 4.25 | |
| Larynx (C32) | 2 |  | 9 | 0.91 | 0.19 to 4.35 | 1.00 | 0.21 to 4.85 | |  | | 7 | | 0.87 | 0.18 to 4.25 | 0.84 | 0.17 to 4.09 | |
| Lung and bronchus (C33-C34) | 40 |  | 209 | 1.01 | 0.71 to 1.42 | 1.03 | 0.72 to 1.46 | |  | | 126 | | 0.99 | 0.69 to 1.41 | 1.01 | 0.71 to 1.45 | |
| Bone (C40-C41) | 1 |  | 6 | 0.91 | 0.10 to 7.97 | 1.01 | 0.11 to 8.98 | |  | | 2 | | 1.74 | 0.15 to 19.58 | 1.64 | 0.15 to 18.67 | |
| Melanoma (C43) | 11 |  | 58 | 0.96 | 0.50 to 1.87 | 1.05 | 0.54 to 2.05 | |  | | 28 | | 1.22 | 0.60 to 2.45 | 1.21 | 0.60 to 2.44 | |
| Other cancer of skin (C44) | 12 |  | 73 | 0.88 | 0.47 to 1.65 | 0.93 | 0.49 to 1.74 | |  | | 41 | | 0.88 | 0.46 to 1.68 | 0.88 | 0.46 to 1.69 | |
| Kaposi´s sarcoma (46) | 1 |  | 3 | 1.42 | 0.14 to 14.63 | 1.94 | 0.16 to 23.32 | |  | | 3 | | 0.76 | 0.07 to 8.17 | 1.27 | 0.12 to 13.48 | |
| Soft tissue sarcoma (C49) | 2 |  | 10 | 0.81 | 0.18 to 3.75 | 0.95 | 0.20 to 4.45 | |  | | 6 | | 1.00 | 0.20 to 4.98 | 1.10 | 0.22 to 5.53 | |
| Breast (C50) | 56 |  | 277 | 1.11 | 0.82 to 1.49 | 1.14 | 0.85 to 1.54 | |  | | 133 | | 1.28 | 0.94 to 1.75 | 1.29 | 0.94 to 1.76 | |
| Vulva (C51) | 2 |  | 4 | 2.56 | 0.45 to 14.57 | 2.72 | 0.46 to 16.06 | |  | | 1 | | 7.15 | 0.63 to 80.94 | 8.26 | 0.73 to 94.00 | |
| Cervix uteri (C53) | 8 |  | 21 | 1.91 | 0.82 to 4.45 | 1.92 | 0.82 to 4.49 | |  | | 14 | | 1.72 | 0.72 to 4.12 | 1.68 | 0.70 to 4.01 | |
| Uterus (C54-C55) | 10 |  | 28 | 1.99 | 0.93 to 4.23 | 2.10 | 0.98 to 4.50 | |  | | 24 | | 1.28 | 0.61 to 2.68 | 1.35 | 0.64 to 2.85 | |
| Ovary (C56-C57) | 7 |  | 44 | 1.06 | 0.46 to 2.41 | 1.10 | 0.48 to 2.51 | |  | | 19 | | 1.12 | 0.47 to 2.66 | 1.17 | 0.49 to 2.80 | |
| Penis (C60) | 2 |  | 2 | 5.90 | 0.66 to 52.35 | 6.94 | 0.78 to 62.10 | |  | | 1 | | 6.19 | 0.55 to 69.15 | 5.49 | 0.47 to 64.31 | |
| Prostate (C61) | 57 |  | 288 | 1.15 | 0.85 to 1.54 | 1.21 | 0.90 to 1.63 | |  | | 138 | | 1.31 | 0.96 to 1.79 | 1.35 | 0.98 to 1.85 | |
| Testis (C62) | 2 |  | 13 | 0.94 | 0.20 to 4.46 | 0.87 | 0.18 to 4.15 | |  | | 4 | | 1.62 | 0.28 to 9.22 | 1.66 | 0.29 to 9.49 | |
| Kidney (C64-C66) | 16 |  | 90 | 1.03 | 0.59 to 1.78 | 1.06 | 0.61 to 1.84 | |  | | 41 | | 1.17 | 0.66 to 2.09 | 1.18 | 0.66 to 2.10 | |
| Bladder (C67) | 19 |  | 91 | 1.07 | 0.64 to 1.78 | 1.11 | 0.67 to 1.86 | |  | | 55 | | 1.07 | 0.63 to 1.81 | 1.13 | 0.67 to 1.91 | |
| Brain and central nervous system (C70-C72, C75.1 and C75.3) | 6 |  | 27 | 1.45 | 0.57 to 3.70 | 1.45 | 0.57 to 3.71 | |  | | 11 | | 1.61 | 0.59 to 4.36 | 1.56 | 0.58 to 4.24 | |
| Thyroid gland (C73) | 11 |  | 51 | 1.19 | 0.60 to 2.34 | 1.25 | 0.63 to 2.47 | |  | | 33 | | 1.00 | 0.50 to 1.97 | 1.01 | 0.51 to 2.01 | |
| Cancer without specification of site (C80) | 5 |  | 26 | 1.05 | 0.39 to 2.79 | 1.05 | 0.39 to 2.82 | |  | | 14 | | 1.21 | 0.43 to 3.39 | 1.21 | 0.43 to 3.41 | |
| Lymphoid and haematopoietic tissue (LH) (C81-C96, D45-D47) | 37 |  | 143 | **1.49** | **1.02 to 2.17** | **1.61** | **1.10 to 2.36** | |  | | 72 | | **1.64** | **1.10 to 2.44** | **1.70** | **1.14 to 2.55** | |
| Hodgkin´s lymphoma (C81) | 1 |  | 10 | 0.62 | 0.08 to 5.07 | 0.60 | 0.07 to 4.91 | |  | | 5 | | 0.97 | 0.10 to 9.21 | 0.89 | 0.09 to 8.41 | |
| Non-Hodgkin´s lymphoma (NHL) (C82-C85) | 17 |  | 49 | **2.12** | **1.18 to 3.80** | **2.30** | **1.27 to 4.14** | |  | | 20 | | **2.98** | **1.50 to 5.89** | **3.02** | **1.52 to 6.00** | |
| NHL, follicular (C82) | 1 |  | 14 | 0.35 | 0.05 to 2.75 | 0.38 | 0.05 to 3.01 | |  | | 5 | | 0.86 | 0.09 to 7.80 | 0.76 | 0.08 to 6.82 | |
| NHL, diffuse (C83) | 9 |  | 30 | 2.04 | 0.93 to 4.51 | **2.27** | **1.02 to 5.05 ^b^** | |  | | 12 | | 2.35 | 0.97 to 5.70 | **2.51** | **1.02 to 6.14 ^a^** | |
| NHL, peripheral T-cells (C84) | 4 |  | 5 | **4.22** | **1.02 to 17.44 ^b^** | **4.24** | **1.02 to 17.64 ^b^** | |  | | 2 | | **8.77** | **1.27 to 60.56 ^a^** | **8.93** | **1.25 to 63.69 ^a^** | |
| NHL, unspecified (C85) | 3 |  | 0 |  |  |  |  | |  | | 1 | | **12.38** | **1.12 to 137.23 ^b^** | 10.23 | 0.96 to 108.93 | |
| Immunoproliferative diseases (C88) | 4 |  | 9 | 2.71 | 0.78 to 9.45 | 2.95 | 0.83 to 10.50 | |  | | 7 | | 2.15 | 0.60 to 7.68 | 2.47 | 0.68 to 9.06 | |
| Multiple Myeloma (C90) | 5 |  | 25 | 1.08 | 0.40 to 2.90 | 1.16 | 0.43 to 3.15 | |  | | 11 | | 1.40 | 0.49 to 4.05 | 1.37 | 0.47 to 3.97 | |
| Leukaemia (C91-C95, D45-D47) | 10 |  | 50 | 1.08 | 0.53 to 2.18 | 1.22 | 0.60 to 2.49 | |  | | 28 | | 1.11 | 0.54 to 2.28 | 1.25 | 0.60 to 2.59 | |
| Non-CLL (C91-C95, D45-D47, except C91.1) | 9 |  | 35 | 1.40 | 0.65 to 3.01 | 1.58 | 0.73 to 3.42 | |  | | 20 | | 1.40 | 0.64 to 3.09 | 1.48 | 0.67 to 3.26 | |
| Lymphoid leukemia (C91) | 1 |  | 17 | 0.30 | 0.04 to 2.32 | 0.35 | 0.05 to 2.68 | |  | | 9 | | 0.37 | 0.05 to 2.93 | 0.51 | 0.06 to 4.20 | |
| Chronic lymphocytic leukaemia (CLL)(C91.1) | 1 |  | 15 | 0.35 | 0.05 to 2.71 | 0.41 | 0.05 to 3.21 | |  | | 8 | | 0.40 | 0.06 to 2.87 | 0.59 | 0.07 to 4.93 | |
| Other and unspecified leukemia (C93 to C95) | 1 |  | 1 | 3.22 | 0.20 to 52.54 | 6.15 | 0.35 to 108.18 | |  | | 0 | |  |  |  |  | |
| Myelodysplastic syndromes (MDS) (D46) | 7 |  | 10 | **3.97** | **1.40 to 11.29 ^a^** | **4.17** | **1.46 to 11.94 ^a^** | |  | | 2 | | **11.30** | **2.25 to 56.67 ^a^** | **11.46** | **2.27 to 57.80 ^a^** | |
| MDS, unspecified (D46.9) | 7 |  | 6 | **6.48** | **2.01 to 20.93 ^a^** | **7.18** | **2.19 to 23.52 ^a^** | |  | | 1 | | **20.59** | **2.51 to 169.15 ^a^** | **20.74** | **2.50 to 171.80 ^a^** | |
| Other LH, uncertain (D47) | 1 |  | 1 | 6.34 | 0.34 to 118.41 | 8.68 | 0.44 to 170.13 | |  | | 0 | |  |  |  | |  |
| Other LH thrombocythemia (D47.3) | 1 |  | 1 | 6.34 | 0.34 to 118.41 | 8.68 | 0.44 to 170.13 | |  | | 0 | |  |  |  | |  |
| **Not included in all cancers** | | | | | | | | | | | | | | | | | |
|  | p-yr 67 151 |  | p-yr 371 689 | | | | |  | | | | p-yr 206 015 | | | | | |
| Basal cell carcinoma of the skin (BCC) (C44) | 74 |  | 357 | 1.06 | 0.82 to 1.37 | 1.11 | 0.86 to 1.44 | |  | | 154 | | **1.44** | **1.09 to 1.91** | **1.48** | **1.12 to 1.96** | |

Abbreviation: p-yr, person years.

**^a^** 95% CI computed with bootstrap method did not include unity. **^b^** 95% CI computed with bootstrap method included unity.

Table E. Number of all cancers and cancer sites with any case among men in the geothermal heating areas, hazard ratio (HR), 95% confidence intervals (CI) compared with the populations in warm reference area and cold reference area applying five years latency time, adjusted for age, gender, education, type of housing, and smoking habits, without and with stratification into categories of cumulative years of residence in the respective areas.

|  | Geothermal heating area |  | Warm reference area | | | | |  | | | Cold reference area | | | | | | | |  |
| --- | --- | --- | --- | --- | --- | --- | --- | --- | --- | --- | --- | --- | --- | --- | --- | --- | --- | --- | --- |
|  | p-yr 32 035 |  | p-yr 181 102 | | | | | |  | | | p-yr 100 523 | | | | | | |  |
|  | No of |  | No of | Not stratified | | Stratified | | | |  | | | No of | Not stratified | | Stratified | | |  |
| Cancers (ICD-10) | cancers |  | cancers | HR | 95%CI | HR | 95%CI | | |  | | | cancers | HR | 95%CI | HR | 95%CI | |  |
| All (C00-C97, D45-D47) | 189 |  | 984 | 1.07 | 0.91 to 1.26 | 1.13 | 0.96 to 1.33 | | |  | | | 478 | **1.22** | **1.03 to 1.45** | **1.26** | **1.07 to 1.50** | |  |
| Lip, oral cavity, and pharynx (C00-C14) | 1 |  | 21 | 0.23 | 0.03 to 1.74 | 0.25 | 0.03 to 1.85 | | |  | | | 10 | 0.65 | 0.07 to 6.22 | 0.68 | 0.07 to 6.60 | |  |
| Oesophagus (C15) | 4 |  | 29 | 0.71 | 0.24 to 2.10 | 0.73 | 0.25 to 2.16 | | |  | | | 13 | 1.05 | 0.34 to 3.24 | 1.15 | 0.37 to 3.59 | |  |
| Stomach (C16) | 4 |  | 31 | 0.67 | 0.23 to 1.96 | 0.68 | 0.23 to 1.98 | | |  | | | 22 | 0.57 | 0.20 to 1.67 | 0.60 | 0.20 to 1.76 | |  |
| Small intestine (C17) | 1 |  | 8 | 0.63 | 0.07 to 5.29 | 0.61 | 0.07 to 5.12 | | |  | | | 5 | 1.01 | 0.10 to 10.36 | 1.15 | 0.11 to 12.21 | |  |
| Colon, rectum, and anus (C18-C21) | 18 |  | 96 | 1.07 | 0.63 to 1.80 | 1.14 | 0.68 to 1.94 | | |  | | | 46 | 1.22 | 0.71 to 2.12 | 1.26 | 0.72 to 2.18 | |  |
| Bile and liver (C22-C24) | 3 |  | 16 | 1.41 | 0.38 to 5.20 | 1.47 | 0.40 to 5.43 | | |  | | | 5 | 2.06 | 0.46 to 9.30 | 2.16 | 0.47 to 9.83 | |  |
| Pancreas (C25) | 6 |  | 17 | 2.05 | 0.76 to 5.54 | 2.30 | 0.84 to 6.25 | | |  | | | 7 | 2.88 | 0.95 to 8.73 | **3.19** | **1.04 to 9.82** | |  |
| Larynx (C32) | 2 |  | 7 | 1.17 | 0.23 to 5.93 | 1.26 | 0.24 to 6.51 | | |  | | | 7 | 0.87 | 0.18 to 4.25 | 0.84 | 0.17 to 4.09 | |  |
| Lung and bronchus (C33-C34) | 23 |  | 111 | 1.16 | 0.72 to 1.85 | 1.24 | 0.77 to 1.99 | | |  | | | 53 | 1.32 | 0.81 to 2.16 | 1.34 | 0.82 to 2.20 | |  |
| Melanoma (C43) | 4 |  | 21 | 1.14 | 0.37 to 3.50 | 1.24 | 0.40 to 3.82 | | |  | | | 8 | 1.58 | 0.47 to 5.34 | 1.52 | 0.45 to 5.17 | |  |
| Other cancer of skin (C44) | 8 |  | 43 | 1.06 | 0.48 to 2.33 | 1.13 | 0.51 to 2.50 | | |  | | | 18 | 1.37 | 0.59 to 3.16 | 1.38 | 0.59 to 3.21 | |  |
| Kaposi´s sarcoma (46) | 1 |  | 3 | 1.42 | 0.14 to 14.63 | 1.94 | 0.16 to 23.32 | | |  | | | 3 | 0.76 | 0.07 to 8.17 | 1.27 | 0.12 to 13.48 | |  |
| Soft tissue sarcoma (C49) | 1 |  | 7 | 0.54 | 0.07 to 4.41 | 0.62 | 0.08 to 5.13 | | |  | | | 2 | 1.58 | 0.14 to 17.62 | 2.18 | 0.19 to 25.60 | |  |
| Breast (C50) | 1 |  | 2 | 5.17 | 0.30 to 87.66 | 4.88 | 0.28 to 84.61 | | |  | | | 0 |  |  |  |  | |  |
| Penis (C60) | 2 |  | 2 | 5.90 | 0.66 to 52.35 | 6.94 | 0.78 to 62.10 | | |  | | | 1 | 6.19 | 0.55 to 69.15 | 5.49 | 0.47 to 64.31 | |  |
| Prostate (C61) | 57 |  | 288 | 1.15 | 0.85 to 1.54 | 1.21 | 0.90 to 1.63 | | |  | | | 138 | 1.31 | 0.96 to 1.79 | 1.35 | 0.98 to 1.85 | |  |
| Testis (C62) | 2 |  | 13 | 0.94 | 0.20 to 4.46 | 0.87 | 0.18 to 4.15 | | |  | | | 4 | 1.62 | 0.28 to 9.22 | 1.66 | 0.29 to 9.49 | |  |
| Kidney (C64-C66) | 9 |  | 56 | 0.91 | 0.44 to 1.89 | 0.93 | 0.45 to 1.94 | | |  | | | 24 | 1.10 | 0.51 to 2.38 | 1.10 | 0.51 to 2.38 | |  |
| Bladder (C67) | 15 |  | 71 | 1.06 | 0.59 to 1.89 | 1.11 | 0.62 to 1.98 | | |  | | | 44 | 1.08 | 0.60 to 1.96 | 1.14 | 0.63 to 2.07 | |  |
| Brain and central nervous system (C70-C72, C75.1 and C75.3) | 2 |  | 17 | 0.80 | 0.17 to 3.69 | 0.81 | 0.18 to 3.72 | | |  | | | 7 | 0.86 | 0.18 to 4.16 | 0.92 | 0.19 to 4.45 | |  |
| Thyroid gland (C73) | 3 |  | 13 | 1.07 | 0.29 to 3.92 | 1.10 | 0.30 to 4.04 | | |  | | | 7 | 1.71 | 0.38 to 7.72 | 1.67 | 0.37 to 7.53 | |  |
| Cancer without specification of site (C80) | 3 |  | 10 | 1.60 | 0.41 to 6.17 | 1.60 | 0.41 to 6.27 | | |  | | | 9 | 1.23 | 0.31 to 4.90 | 1.21 | 0.30 to 4.87 | |  |
| Lymphoid and haematopoietic tissue (LH) (C81-C96, D45-D47) | 19 |  | 86 | 1.28 | 0.76 to 2.16 | 1.35 | 0.80 to 2.29 | | |  | | | 39 | 1.55 | 0.89 to 2.70 | 1.64 | 0.93 to 2.87 | |  |
| Non-Hodgkin´s lymphoma (NHL) (C82-C85) | 10 |  | 29 | **2.35** | **1.08 to 5.13** | **2.51** | **1.15 to 5.50** | | |  | | | 16 | 1.93 | 0.86 to 4.33 | 1.97 | 0.87 to 4.43 | |  |
| NHL, diffuse (C83) | 6 |  | 18 | 2.50 | 0.91 to 6.85 | 2.63 | 0.96 to 7.22 | | |  | | | 10 | 1.80 | 0.65 to 5.00 | 1.91 | 0.68 to 5.35 | |  |
| NHL, peripheral T-cells (C84) | 3 |  | 5 | 3.55 | 0.74 to 16.99 | 3.72 | 0.77 to 17.89 | | |  | | | 2 | **14.18** | **1.21 to 166.01** | **15.69** | **1.21 to 204.03** | |  |
| NHL, unspecified (C85) | 1 |  | 0 |  |  |  |  | | |  | | | 1 | 3.44 | 0.20 to 58.57 | 2.90 | 0.17 to 48.62 | |  |
| Immunoproliferative diseases (C88) | 2 |  | 3 | 3.44 | 0.45 to 26.26 | 3.19 | 0.39 to 26.08 | | |  | | | 3 | 2.26 | 0.34 to 15.03 | 2.52 | 0.37 to 17.35 | |  |
| Multiple Myeloma (C90) | 1 |  | 15 | 0.42 | 0.05 to 3.38 | 0.45 | 0.06 to 3.61 | | |  | | | 3 | 1.11 | 0.12 to 10.75 | 1.08 | 0.11 to 10.52 | |  |
| Leukaemia (C91-C95, D45-D47) | 6 |  | 33 | 0.86 | 0.35 to 2.11 | 0.94 | 0.38 to 2.30 | | |  | | | 13 | 1.59 | 0.58 to 4.35 | 1.94 | 0.69 to 5.42 | |  |
| Non-CLL (C91-C95, D45-D47, except C91.1) | 6 |  | 23 | 1.24 | 0.49 to 3.15 | 1.36 | 0.53 to 3.48 | | |  | | | 7 | 2.76 | 0.91 to 8.38 | 2.95 | 0.95 to 9.11 | |  |
| Other and unspecified leukemia (C93 to C95) | 1 |  | 1 | 3.22 | 0.20 to 52.54 | 6.15 | 0.35 to 108.18 | | |  | | | 0 |  |  |  |  | |  |
| Myelodysplastic syndromes(MDS) (D46) | 4 |  | 7 | 2.37 | 0.66 to 8.49 | 2.74 | 0.75 to 9.97 | | |  | | | 1 | **22.44** | **1.71 to 293.99** | **24.27** | **2.08 to 283.07** | |  |
| MDS, unspecified (D46.9) | 4 |  | 3 | **5.11** | **1.12 to 23.22** | **7.10** | **1.47 to 34.17** | | |  | | | 0 |  |  |  |  | |  |
| Other LH, uncertain (D47) | 1 |  | 0 |  |  |  |  | | |  | | | 0 |  |  |  |  | |  |
| Other LH thrombocythemia (D47.3) | 1 |  | 0 |  |  |  |  | | |  | | | 0 |  |  |  |  | |  |
| **Not included in all cancers** | | | | | | | | | | | | | | | | | |  | |
|  | p-yr 32 766 |  | p-yr 185 283 | | | | | |  | | | p-yr 102 340 | | | | | | | |
| Basal cell carcinoma of the skin (BCC) (C44) | 33 |  | 149 | 1.12 | 0.76 to 1.66 | 1.20 | 0.81 to 1.77 | | |  | | | 58 | **1.70** | **1.11 to 2.61** | **1.75** | **1.13 to 2.69** | | |

Abbreviation: p-yr, person years.

Table F. Number of all cancers and select cancer sites any case among women in the geothermal heating areas, hazard ratio (HR), 95% confidence intervals (CI) compared with the populations in warm reference area and cold reference area applying five years latency time, adjusted for age, gender, education, type of housing, and smoking habits, without and with stratification into categories of cumulative years of residence in the respective areas.

|  | Geothermal heating area |  | Warm reference area | | | | |  | | Cold reference area | | | | | |  | | |
| --- | --- | --- | --- | --- | --- | --- | --- | --- | --- | --- | --- | --- | --- | --- | --- | --- | --- | --- |
|  | p-yr 33 134 |  | p-yr 181 196 | | | | |  | | p-yr 101 183 | | | | | | | | |
|  | No of |  | No of | Not stratified | | Stratified | | |  | | No of | Not stratified | | Stratified | | | |  |
| Cancers (ICD-10) | cancers |  | cancers | HR | 95%CI | HR | 95%CI | |  | | cancers | HR | 95%CI | HR | 95%CI | | |  |
| All (C00-C97, D45-D47) | 183 |  | 861 | 1.15 | 0.98 to 1.36 | **1.20** | **1.02 to 1.42** | |  | | 481 | 1.16 | 0.98 to 1.38 | 1.18 | 0.99 to 1.40 | | |  |
| Lip, oral cavity, and pharynx (C00-C14) | 2 |  | 16 | 0.61 | 0.14 to 2.72 | 0.64 | 0.14 to 2.87 | |  | | 2 | 2.93 | 0.40 to 21.50 | 2.64 | 0.36 to 19.45 | | |  |
| Oesophagus (C15) | 1 |  | 6 | 1.18 | 0.13 to 10.42 | 1.34 | 0.15 to 12.18 | |  | | 3 | 0.98 | 0.10 to 9.58 | 0.98 | 0.10 to 9.68 | | |  |
| Stomach (C16) | 5 |  | 18 | 1.47 | 0.53 to 4.04 | 1.72 | 0.61 to 4.84 | |  | | 7 | 2.29 | 0.72 to 7.26 | 2.30 | 0.72 to 7.35 | | |  |
| Colon, rectum, and anus (C18-C21) | 14 |  | 67 | 1.12 | 0.62 to 2.02 | 1.17 | 0.65 to 2.13 | |  | | 32 | 1.34 | 0.72 to 2.52 | 1.34 | 0.71 to 2.53 | | |  |
| Bile and liver (C22-C24) | 1 |  | 6 | 0.70 | 0.08 to 5.84 | 0.81 | 0.09 to 6.92 | |  | | 2 | 1.93 | 0.16 to 22.74 | 1.84 | 0.16 to 21.74 | | |  |
| Pancreas (C25) | 5 |  | 19 | 1.74 | 0.62 to 4.85 | 1.94 | 0.68 to 5.49 | |  | | 12 | 1.39 | 0.49 to 3.95 | 1.42 | 0.50 to 4.06 | | |  |
| Lung and bronchus (C33-C34) | 17 |  | 98 | 0.86 | 0.51 to 1.45 | 0.85 | 0.50 to 1.44 | |  | | 73 | 0.73 | 0.43 to 1.24 | 0.75 | 0.44 to 1.28 | | |  |
| Bone (C40-C41) | 1 |  | 1 | 5.51 | 0.29 to 105.91 | 4.89 | 0.26 to 91.92 | |  | | 2 | 1.74 | 0.15 to 19.58 | 1.64 | 0.15 to 18.67 | | |  |
| Melanoma (C43) | 7 |  | 37 | 0.92 | 0.40 to 2.09 | 0.99 | 0.43 to 2.27 | |  | | 20 | 1.16 | 0.49 to 2.76 | 1.12 | 0.47 to 2.68 | | |  |
| Other cancer of skin (C44) | 4 |  | 30 | 0.66 | 0.23 to 1.91 | 0.68 | 0.23 to 1.98 | |  | | 23 | 0.51 | 0.18 to 1.47 | 0.51 | 0.18 to 1.49 | | |  |
| Soft tissue sarcoma (C49) | 1 |  | 3 | 1.51 | 0.15 to 15.02 | 1.83 | 0.17 to 19.25 | |  | | 4 | 0.79 | 0.09 to 7.17 | 0.79 | 0.09 to 7.25 | | |  |
| Breast (C50) | 55 |  | 275 | 1.09 | 0.81 to 1.47 | 1.13 | 0.83 to 1.52 | |  | | 133 | 1.26 | 0.92 to 1.72 | 1.26 | 0.92 to 1.74 | | |  |
| Vulva (C51) | 2 |  | 4 | 2.56 | 0.45 to 14.57 | 2.72 | 0.46 to 16.06 | |  | | 1 | 7.15 | 0.63 to 80.94 | 8.26 | 0.73 to 94.00 | | |  |
| Cervix uteri (C53) | 8 |  | 21 | 1.91 | 0.82 to 4.45 | 1.92 | 0.82 to 4.49 | |  | | 14 | 1.72 | 0.72 to 4.12 | 1.68 | 0.70 to 4.01 | | |  |
| Uterus (C54-C55) | 10 |  | 28 | 1.99 | 0.93 to 4.23 | 2.10 | 0.98 to 4.50 | |  | | 24 | 1.28 | 0.61 to 2.68 | 1.35 | 0.64 to 2.85 | | |  |
| Ovary (C56-C57) | 7 |  | 44 | 1.06 | 0.46 to 2.41 | 1.10 | 0.48 to 2.51 | |  | | 19 | 1.12 | 0.47 to 2.66 | 1.17 | 0.49 to 2.80 | | |  |
| Kidney (C64-C66) | 7 |  | 34 | 1.23 | 0.53 to 2.84 | 1.29 | 0.55 to 3.02 | |  | | 17 | 1.26 | 0.52 to 3.06 | 1.32 | 0.54 to 3.21 | | |  |
| Bladder (C67) | 4 |  | 20 | 1.05 | 0.35 to 3.15 | 1.13 | 0.37 to 3.41 | |  | | 11 | 1.13 | 0.36 to 3.58 | 1.18 | 0.37 to 3.73 | | |  |
| Brain and central nervous system (C70-C72, C75.1 and C75.3) | 4 |  | 10 | 2.47 | 0.73 to 8.41 | 2.45 | 0.71 to 8.44 | |  | | 4 | 2.73 | 0.68 to 10.99 | 2.54 | 0.63 to 10.20 | | |  |
| Thyroid gland (C73) | 8 |  | 38 | 1.22 | 0.55 to 2.68 | 1.29 | 0.58 to 2.87 | |  | | 26 | 0.92 | 0.42 to 2.03 | 0.94 | 0.42 to 2.08 | | |  |
| Cancer without specification of site (C80) | 2 |  | 16 | 0.69 | 0.15 to 3.07 | 0.71 | 0.16 to 3.19 | |  | | 5 | 1.35 | 0.26 to 6.99 | 1.39 | 0.26 to 7.29 | | |  |
| Lymphoid and haematopoietic tissue (LH) (C81-C96, D45-D47) | 18 |  | 57 | **1.78** | **1.03 to 3.09** | **1.98** | **1.14 to 3.46** | |  | | 33 | 1.67 | 0.94 to 2.97 | 1.69 | 0.95 to 3.03 | | |  |
| Hodgkin´s lymphoma (C81) | 1 |  | 4 | 1.20 | 0.13 to 11.10 | 1.17 | 0.13 to 10.94 | |  | | 2 | 2.15 | 0.19 to 24.64 | 2.13 | 0.19 to 24.60 | | |  |
| Non-Hodgkin´s lymphoma (NHL) (C82-C85) | 7 |  | 20 | 1.99 | 0.81 to 4.88 | 2.17 | 0.88 to 5.37 | |  | | 4 | **6.21** | **1.78 to 21.66** | **5.83** | **1.66 to 20.40** | | |  |
| NHL, follicular (C82) | 1 |  | 8 | 0.60 | 0.07 to 4.89 | 0.64 | 0.08 to 5.28 | |  | | 2 | 3.13 | 0.23 to 41.95 | 2.39 | 0.18 to 31.52 | | |  |
| NHL, diffuse (C83) | 3 |  | 12 | 1.56 | 0.42 to 5.79 | 1.86 | 0.49 to 7.00 | |  | | 2 | 4.71 | 0.77 to 28.84 | 5.21 | 0.83 to 32.54 | | |  |
| NHL, peripheral T-cells (C84) | 1 |  | 0 |  |  |  |  | |  | | 0 |  |  |  |  | | |  |
| NHL, unspecified (C85) | 2 |  | 0 |  |  |  |  | |  | | 0 |  |  |  |  | | |  |
| Immunoproliferative diseases (C88) | 2 |  | 6 | 2.05 | 0.39 to 10.77 | 2.09 | 0.39 to 11.33 | |  | | 4 | 1.86 | 0.33 to 10.34 | 2.20 | 0.38 to 12.60 | | |  |
| Multiple Myeloma (C90) | 4 |  | 10 | 1.90 | 0.58 to 6.25 | 2.00 | 0.60 to 6.62 | |  | | 8 | 1.48 | 0.44 to 4.92 | 1.44 | 0.43 to 4.83 | | |  |
| Leukaemia (C91-C95, D45-D47) | 4 |  | 17 | 1.52 | 0.49 to 4.72 | 1.93 | 0.61 to 6.09 | |  | | 15 | 0.81 | 0.27 to 2.44 | 0.89 | 0.29 to 2.71 | | |  |
| Non-CLL (C91-C95, D45-D47, except C91.1) | 3 |  | 12 | 1.66 | 0.44 to 6.20 | 1.99 | 0.53 to 7.58 | |  | | 13 | 0.72 | 0.20 to 2.53 | 0.77 | 0.22 to 2.73 | | |  |
| Lymphoid leukemia (C91) | 1 |  | 5 | 1.23 | 0.13 to 11.37 | 1.88 | 0.20 to 18.09 | |  | | 2 | 1.31 | 0.10 to 17.47 | 2.27 | 0.16 to 33.35 | | |  |
| Chronic lymphocytic leukaemia (CLL)(C91.1) | 1 |  | 5 | 1.23 | 0.13 to 11.37 | 1.88 | 0.20 to 18.09 | |  | | 2 | 1.31 | 0.10 to 17.47 | 2.27 | 0.16 to 33.35 | | |  |
| Myelodysplastic syndromes (MDS) (D46) | 3 |  | 3 | **8.57** | **1.58 to 46.63** | **9.45** | **1.72 to 51.79** | |  | | 1 | 8.31 | 0.85 to 81.40 | 8.13 | 0.83 to 79.51 | | |  |
| MDS, unspecified (D46.9) | 3 |  | 3 | **8.57** | **1.58 to 46.63** | **9.45** | **1.72 to 51.79** | |  | | 1 | 8.31 | 0.85 to 81.40 | 8.13 | 0.83 to 79.51 | | |  |
| **Not included in all cancers** | | | | | | | | | | | | | | | | |  | |
|  | p-yr 34 385 |  | p-yr 186 406 | | | | |  | | p-yr 103 615 | | | | | | | | |
| Basal cell carcinoma of the skin (BCC) (C44) | 41 |  | 208 | 1.00 | 0.71 to 1.40 | 1.05 | 0.74 to 1.48 | |  | | 96 | 1.26 | 0.87 to 1.82 | 1.30 | 0.90 to 1.88 | | |  |

Abbreviation: p-yr, person years.

Table G. Number of all cancers and cancer sites with any case among men in the geothermal heating areas, hazard ratio (HR), 95% confidence intervals (CI) compared with the populations in warm reference area and cold reference area, adjusted for age, gender, education, type of housing, and smoking habits without and with stratification into categories of cumulative years of residence in the respective areas.

|  | Geothermal heating area |  | Warm reference area | | | | | | |  | | | | Cold reference area | | | | | | | | | | | | |  | |
| --- | --- | --- | --- | --- | --- | --- | --- | --- | --- | --- | --- | --- | --- | --- | --- | --- | --- | --- | --- | --- | --- | --- | --- | --- | --- | --- | --- | --- |
|  | p-yr 48 817 |  | p-yr 281 694 | | | | | | |  | | | | p-yr 152 060 | | | | | | | | | | | | |  | |
|  | No of |  | No of | Not stratified | | Stratified | | | | |  | | | | No of | | Not stratified | | | | Stratified | | | | |  | | |
| Cancers (ICD-10) | cancers |  | cancers | HR | 95%CI | HR | 95%CI | | | |  | | | | cancers | | HR | | 95%CI | | HR | | 95%CI | | |  | | |
| All (C00-C97, D45-D47) | 517 |  | 2839 | 1.05 | 0.95 to 1.15 | 1.07 | 0.97 to 1.18 | | | |  | | | | 1346 | | **1.18** | | **1.06 to 1.31** | | **1.21** | | **1.09 to 1.34** | | |  | | |
| Lip, oral cavity, and pharynx (C00-C14) | 11 |  | 64 | 0.83 | 0.43 to 1.60 | 0.82 | 0.43 to 1.59 | | | |  | | | | 29 | | 1.31 | | 0.63 to 2.72 | | 1.38 | | 0.66 to 2.88 | | |  | | |
| Oesophagus (C15) | 11 |  | 57 | 0.98 | 0.50 to 1.90 | 1.00 | 0.51 to 1.94 | | | |  | | | | 25 | | 1.49 | | 0.72 to 3.11 | | 1.54 | | 0.74 to 3.23 | | |  | | |
| Stomach (C16) | 17 |  | 125 | 0.78 | 0.47 to 1.32 | 0.77 | 0.45 to 1.30 | | | |  | | | | 86 | | 0.61 | | 0.36 to 1.03 | | 0.60 | | 0.35 to 1.01 | | |  | | |
| Small intestine (C17) | 1 |  | 16 | 0.27 | 0.04 to 2.05 | 0.26 | 0.03 to 2.02 | | | |  | | | | 6 | | 0.93 | | 0.09 to 9.48 | | 1.05 | | 0.10 to 11.08 | | |  | | |
| Colon, rectum, and anus (C18-C21) | 55 |  | 269 | 1.21 | 0.90 to 1.64 | 1.24 | 0.92 to 1.68 | | | |  | | | | 129 | | 1.27 | | 0.92 to 1.74 | | 1.29 | | 0.93 to 1.77 | | |  | | |
| Bile and liver (C22-C24) | 7 |  | 53 | 0.80 | 0.36 to 1.81 | 0.82 | 0.36 to 1.85 | | | |  | | | | 14 | | 1.53 | | 0.61 to 3.84 | | 1.58 | | 0.62 to 4.01 | | |  | | |
| Pancreas (C25) | 18 |  | 67 | 1.63 | 0.94 to 2.81 | 1.65 | 0.95 to 2.86 | | | |  | | | | 22 | | **2.60** | | **1.38 to 4.90** | | **2.69** | | **1.42 to 5.09** | | |  | | |
| Nasal cavity and middle ear (C30) | 1 |  | 4 | 1.92 | 0.18 to 20.26 | 1.70 | 0.16 to 18.00 | | | |  | | | | 1 | | 14.24 | | 0.27 to 755.38 | | 13.29 | | 0.29 to 614.04 | | |  | | |
| Larynx (C32) | 4 |  | 26 | 0.93 | 0.31 to 2.77 | 0.92 | 0.31 to 2.75 | | | |  | | | | 11 | | 1.14 | | 0.36 to 3.65 | | 1.14 | | 0.36 to 3.63 | | |  | | |
| Lung and bronchus (C33-C34) | 44 |  | 320 | 0.79 | 0.57 to 1.09 | 0.81 | 0.59 to 1.13 | | | |  | | | | 155 | | 0.86 | | 0.61 to 1.20 | | 0.88 | | 0.63 to 1.23 | | |  | | |
| Bone (C40-C41) | 1 |  | 13 | 0.42 | 0.05 to 3.30 | 0.40 | 0.05 to 3.16 | | | |  | | | | 1 | | 6.32 | | 0.22 to 179.65 | | 5.63 | | 0.19 to 164.71 | | |  | | |
| Melanoma (C43) | 9 |  | 57 | 0.91 | 0.44 to 1.89 | 0.93 | 0.45 to 1.94 | | | |  | | | | 17 | | 1.57 | | 0.70 to 3.53 | | 1.62 | | 0.72 to 3.66 | | |  | | |
| Other cancer of skin (C44) | 19 |  | 98 | 1.06 | 0.64 to 1.77 | 1.09 | 0.65 to 1.81 | | | |  | | | | 50 | | 1.19 | | 0.70 to 2.03 | | 1.21 | | 0.71 to 2.07 | | |  | | |
| Kaposi´s sarcoma (C46) | 1 |  | 8 | 0.66 | 0.08 to 5.69 | 0.70 | 0.08 to 6.31 | | | |  | | | | 5 | | 0.51 | | 0.06 to 4.59 | | 0.57 | | 0.06 to 5.12 | | |  | | |
| Peritoneum (C48) | 1 |  | 4 | 1.28 | 0.14 to 12.24 | 1.22 | 0.13 to 11.74 | | | |  | | | | 0 | |  | |  | |  | |  | | |  | | |
| Soft tissue sarcoma (C49) | 2 |  | 22 | 0.45 | 0.10 to 1.98 | 0.45 | 0.10 to 1.97 | | | |  | | | | 6 | | 1.19 | | 0.23 to 6.29 | | 1.28 | | 0.24 to 6.75 | | |  | | |
| Breast (C50) | 3 |  | 9 | 1.58 | 0.40 to 6.16 | 1.57 | 0.40 to 6.17 | | | |  | | | | 5 | | 2.03 | | 0.47 to 8.74 | | 2.16 | | 0.50 to 9.43 | | |  | | |
| Penis (C60) | 2 |  | 11 | 1.00 | 0.21 to 4.79 | 1.11 | 0.23 to 5.36 | | | |  | | | | 3 | | 2.14 | | 0.32 to 14.22 | | 1.90 | | 0.28 to 12.95 | | |  | | |
| Prostate (C61) | 172 |  | 803 | **1.27** | **1.07 to 1.51** | **1.32** | **1.11 to 1.57** | | | |  | | | | 377 | | **1.43** | | **1.19 to 1.72** | | **1.47** | | **1.22 to 1.77** | | |  | | |
| Testis (C62) | 2 |  | 42 | 0.30 | 0.07 to 1.25 | 0.28 | 0.07 to 1.19 | | | |  | | | | 23 | | 0.28 | | 0.07 to 1.17 | | 0.29 | | 0.07 to 1.22 | | |  | | |
| Kidney (C64-C66) | 28 |  | 147 | 1.06 | 0.70 to 1.61 | 1.08 | 0.71 to 1.64 | | | |  | | | | 62 | | 1.37 | | 0.88 to 2.15 | | 1.38 | | 0.88 to 2.17 | | |  | | |
| Bladder (C67) | 35 |  | 191 | 0.97 | 0.67 to 1.41 | 0.99 | 0.68 to 1.44 | | | |  | | | | 110 | | 0.96 | | 0.66 to 1.41 | | 1.01 | | 0.69 to 1.49 | | |  | | |
| Urinary organs, unspecified (C68) | 1 |  | 2 | 2.68 | 0.21 to 33.75 | 2.22 | 0.17 to 29.77 | | | |  | | | | 1 | | 3.36 | | 0.20 to 55.46 | | 3.54 | | 0.22 to 57.47 | | |  | | |
| Brain and central nervous system (C70-C72, C75.1 and C75.3) | 4 |  | 60 | 0.42 | 0.15 to 1.17 | 0.41 | 0.15 to 1.16 | | | |  | | | | 29 | | 0.48 | | 0.16 to 1.38 | | 0.47 | | 0.16 to 1.38 | | |  | | |
| Thyroid gland (C73) | 7 |  | 43 | 0.96 | 0.42 to 2.20 | 0.94 | 0.41 to 2.17 | | | |  | | | | 18 | | 1.33 | | 0.54 to 3.29 | | 1.36 | | 0.55 to 3.37 | | |  | | |
| Cancer without specification of site (C80) | 6 |  | 51 | 0.79 | 0.33 to 1.90 | 0.78 | 0.32 to 1.87 | | | |  | | | | 29 | | 0.65 | | 0.27 to 1.58 | | 0.71 | | 0.29 to 1.74 | | |  | | |
| Lymphoid and haematopoietic tissue (LH) (C81-C96, D45-D47) | 55 |  | 253 | 1.24 | 0.92 to 1.68 | 1.27 | 0.94 to 1.73 | | | |  | | | | 118 | | **1.48** | | **1.07 to 2.06** | | **1.53** | | **1.10 to 2.13** | | |  | | |
| Hodgkin´s lymphoma (C81) | 2 |  | 19 | 0.59 | 0.13 to 2.60 | 0.56 | 0.13 to 2.49 | | | |  | | | | 9 | | 0.82 | | 0.17 to 4.05 | | 0.78 | | 0.16 to 3.86 | | |  | | |
| Non-Hodgkin´s lymphoma (NHL) (C82-C85) | 24 |  | 82 | **1.85** | **1.14 to 2.99** | **1.92** | **1.18 to 3.12** | | | |  | | | | 46 | | 1.60 | | 0.97 to 2.66 | | 1.65 | | 0.99 to 2.74 | | |  | | |
| NHL, follicular (C82) | 3 |  | 16 | 0.95 | 0.27 to 3.41 | 1.03 | 0.28 to 3.73 | | | |  | | | | 9 | | 1.14 | | 0.31 to 4.25 | | 1.19 | | 0.32 to 4.44 | | |  | | |
| NHL, diffuse (C83) | 12 |  | 51 | 1.55 | 0.80 to 3.00 | 1.59 | 0.82 to 3.10 | | | |  | | | | 29 | | 1.31 | | 0.65 to 2.63 | | 1.36 | | 0.67 to 2.75 | | |  | | |
| NHL, peripheral T-cells (C84) | 5 |  | 10 | **3.48** | **1.07 to 11.31** | **3.57** | **1.10 to 11.65** | | | |  | | | | 5 | | 3.31 | | 0.86 to 12.68 | | 3.41 | | 0.87 to 13.28 | | |  | | |
| NHL, unspecified (C85) | 4 |  | 5 | **5.00** | **1.20 to 20.87** | **5.00** | **1.19 to 20.97** | | | |  | | | | 3 | | 4.11 | | 0.86 to 19.66 | | 3.86 | | 0.81 to 18.34 | | |  | | |
| Immunoproliferative diseases (C88) | 3 |  | 13 | 1.41 | 0.37 to 5.39 | 1.53 | 0.40 to 5.95 | | | |  | | | | 6 | | 1.47 | | 0.36 to 5.93 | | 1.66 | | 0.41 to 6.74 | | |  | | |
| Multiple Myeloma (C90) | 7 |  | 44 | 0.95 | 0.42 to 2.19 | 0.98 | 0.42 to 2.25 | | | |  | | | | 13 | | 1.79 | | 0.70 to 4.59 | | 1.83 | | 0.71 to 4.72 | | |  | | |
| Leukaemia (C91-C95, D45-D47) | 19 |  | 94 | 1.04 | 0.63 to 1.74 | 1.06 | 0.64 to 1.76 | | | |  | | | | 43 | | 1.51 | | 0.86 to 2.65 | | 1.61 | | 0.91 to 2.83 | | |  | | |
| Non-CLL (C91-C95, D45-D47, except C91.1) | 13 |  | 65 | 1.05 | 0.57 to 1.95 | 1.07 | 0.58 to 1.97 | | | |  | | | | 28 | | 1.52 | | 0.77 to 3.00 | | 1.57 | | 0.80 to 3.10 | | |  | | |
| Lymphoid leukemia (C91) | 7 |  | 41 | 0.85 | 0.38 to 1.94 | 0.86 | 0.38 to 1.96 | | | |  | | | | 19 | | 1.45 | | 0.56 to 3.70 | | 1.61 | | 0.62 to 4.20 | | |  | | |
| Chronic lymphocytic leukaemia (CLL)(C91.1) | 6 |  | 29 | 1.03 | 0.42 to 2.57 | 1.07 | 0.43 to 2.67 | | | |  | | | | 15 | | 1.53 | | 0.56 to 4.22 | | 1.82 | | 0.65 to 5.13 | | |  | | |
| Other lymphoid leukemia (C91.2 to C91.9) | 1 |  | 4 | 1.15 | 0.12 to 10.71 | 1.10 | 0.12 to 10.33 | | | |  | | | | 2 | | 1.42 | | 0.11 to 17.71 | | 1.12 | | 0.09 to 14.61 | | |  | | |
| Myeloid leukemia (C92) | 3 |  | 22 | 0.81 | 0.24 to 2.78 | 0.79 | 0.23 to 2.71 | | | |  | | | | 15 | | 0.76 | | 0.21 to 2.72 | | 0.81 | | 0.23 to 2.91 | | |  | | |
| Acute myeloid leukemia (C92.0) | 1 |  | 16 | 0.44 | 0.06 to 3.45 | 0.45 | 0.06 to 3.48 | | | |  | | | | 11 | | 0.38 | | 0.05 to 3.14 | | 0.39 | | 0.05 to 3.26 | | |  | | |
| Other myeloid leukemia (C92.1 to C92.9) | 2 |  | 6 | 1.51 | 0.30 to 7.58 | 1.42 | 0.28 to 7.15 | | | |  | | | | 4 | | 1.96 | | 0.35 to 10.84 | | 2.19 | | 0.40 to 12.07 | | |  | | |
| Other and unspecified leukemia (C93 to C95) | 1 |  | 5 | 0.86 | 0.10 to 7.73 | 0.92 | 0.10 to 8.36 | | | |  | | | | 0 | |  | |  | |  | |  | | |  | | |
| Myelodysplastic syndromes (MDS) (D46) | 5 |  | 14 | 1.55 | 0.54 to 4.46 | 1.71 | 0.59 to 4.94 | | | |  | | | | 4 | | **4.13** | | **1.10 to 15.55** | | **4.19** | | **1.11 to 15.90** | |  | | | |
| MDS, unspecified (D46.9) | 5 |  | 9 | 2.27 | 0.74 to 6.92 | 2.59 | 0.84 to 7.97 | | | |  | | | | 1 | | **19.88** | | **1.77 to 222.62** | | **18.99** | | **1.79 to 201.53** | | | | | |
| Other LH, uncertain (D47) | 3 |  | 8 | 2.78 | 0.65 to 11.87 | 2.81 | 0.66 to 11.94 | | | |  | | | | 4 | | 2.04 | | 0.45 to 9.18 | | 1.88 | | 0.42 to 8.44 | | | | | |
| Other LH thrombocythemia (D47.3) | 3 |  | 0 |  |  |  |  | | | |  | | | | 0 | |  | |  | |  | |  | | |  | | |
| **Not included in all cancers** | | | | | | | | | | | | | | | | | | | | | | | | | | | |  |
|  | p-yr 50 117 |  | p-yr 289 385 | | | | |  | | | | p-yr 155 853 | | | | | | | | | | | |  | | | | |
| Basal cell carcinoma of the skin (BCC) (C44) | 81 |  | 344 | 1.27 | 0.99 to 1.64 | **1.32** | **1.02 to 1.70** | |  | | | | 146 | | | **1.64** | | **1.25 to 2.16** | | **1.70** | | **1.30 to 2.24** | | |  | | | |

Abbreviation: p-yr, person years.

Table H. Number of all cancers and cancer sites with any case among women in the geothermal heating areas, hazard ratio (HR), 95% confidence intervals (CI) compared with the populations in warm reference area and cold reference area, adjusted for age, gender, education, type of housing, and smoking habits without and with stratification into categories of cumulative years of residence in the respective areas.

|  | Geothermal heating area |  | Warm reference area | | | | |  | | Cold reference area | | | | | | |
| --- | --- | --- | --- | --- | --- | --- | --- | --- | --- | --- | --- | --- | --- | --- | --- | --- |
|  | p-yr 49 094 |  | p-yr 276 121 | | | | |  | | p-yr 147 818 | | | | | |  |
|  | No of |  | No of | Not stratified | | Stratified | | |  | | No of | Not stratified | | Stratified | |  |
| Cancers (ICD-10) | cancers |  | cancers | HR | 95%CI | HR | 95%CI | |  | | cancers | HR | 95%CI | HR | 95%CI |  |
| All (C00-C97, D45-D47) | 471 |  | 2492 | 1.08 | 0.98 to 1.20 | **1.14** | **1.03 to 1.26** | |  | | 1178 | **1.16** | **1.04 to 1.29** | **1.21** | **1.09 to 1.35** |  |
| Lip, oral cavity, and pharynx (C00-C14) | 5 |  | 39 | 0.68 | 0.26 to 1.76 | 0.73 | 0.28 to 1.90 | |  | | 12 | 1.20 | 0.42 to 3.43 | 1.18 | 0.41 to 3.39 |  |
| Oesophagus (C15) | 2 |  | 15 | 0.77 | 0.17 to 3.51 | 0.86 | 0.19 to 3.96 | |  | | 11 | 0.52 | 0.11 to 2.33 | 0.51 | 0.11 to 2.32 |  |
| Stomach (C16) | 13 |  | 71 | 1.01 | 0.55 to 1.85 | 1.08 | 0.58 to 1.98 | |  | | 28 | 1.33 | 0.69 to 2.56 | 1.45 | 0.74 to 2.81 |  |
| Small intestine (C17) | 3 |  | 9 | 2.77 | 0.70 to 11.02 | 3.15 | 0.79 to 12.56 | |  | | 7 | 1.48 | 0.37 to 5.84 | 1.56 | 0.38 to 6.35 |  |
| Colon, rectum, and anus (C18-C21) | 35 |  | 204 | 1.00 | 0.69 to 1.44 | 1.06 | 0.73 to 1.53 | |  | | 90 | 1.13 | 0.76 to 1.66 | 1.17 | 0.79 to 1.73 |  |
| Bile and liver (C22-C24) | 3 |  | 26 | 0.58 | 0.17 to 1.94 | 0.59 | 0.17 to 2.00 | |  | | 20 | 0.45 | 0.14 to 1.54 | 0.47 | 0.14 to 1.59 |  |
| Pancreas (C25) | 12 |  | 59 | 1.34 | 0.70 to 2.55 | 1.40 | 0.73 to 2.69 | |  | | 27 | 1.32 | 0.67 to 2.61 | 1.35 | 0.68 to 2.68 |  |
| Lung and bronchus (C33-C34) | 48 |  | 258 | 1.05 | 0.76 to 1.44 | 1.10 | 0.80 to 1.51 | |  | | 145 | 0.98 | 0.71 to 1.36 | 1.04 | 0.75 to 1.45 |  |
| Bone (C40-C41) | 3 |  | 8 | 2.11 | 0.52 to 8.56 | 1.88 | 0.47 to 7.58 | |  | | 5 | 1.74 | 0.41 to 7.34 | 1.84 | 0.43 to 7.82 |  |
| Melanoma (C43) | 15 |  | 81 | 0.97 | 0.55 to 1.70 | 1.05 | 0.59 to 1.85 | |  | | 40 | 1.14 | 0.63 to 2.06 | 1.16 | 0.64 to 2.11 |  |
| Other cancer of skin (C44) | 7 |  | 86 | 0.47 | 0.21 to 1.02 | 0.50 | 0.23 to 1.10 | |  | | 38 | 0.52 | 0.23 to 1.16 | 0.52 | 0.23 to 1.17 |  |
| Peritoneum (C48) | 1 |  | 11 | 0.40 | 0.05 to 3.16 | 0.41 | 0.05 to 3.23 | |  | | 7 | 0.45 | 0.06 to 3.63 | 0.45 | 0.06 to 3.67 |  |
| Soft tissue sarcoma (C49) | 4 |  | 15 | 1.73 | 0.55 to 5.51 | 1.89 | 0.60 to 6.03 | |  | | 9 | 1.32 | 0.40 to 4.33 | 1.39 | 0.42 to 4.57 |  |
| Breast (C50) | 158 |  | 731 | **1.22** | **1.02 to 1.46** | **1.27** | **1.06 to 1.51** | |  | | 321 | **1.42** | **1.17 to 1.72** | **1.48** | **1.22 to 1.79** |  |
| Vulva (C51) | 3 |  | 9 | 1.75 | 0.45 to 6.78 | 1.69 | 0.43 to 6.65 | |  | | 5 | 1.88 | 0.44 to 7.95 | 2.17 | 0.51 to 9.31 |  |
| Vagina (C52) | 1 |  | 2 | 3.08 | 0.23 to 41.26 | 3.33 | 0.24 to 46.49 | |  | | 3 | 0.91 | 0.09 to 8.84 | 1.36 | 0.13 to 13.87 |  |
| Cervix uteri (C53) | 14 |  | 81 | 0.93 | 0.52 to 1.67 | 0.90 | 0.51 to 1.62 | |  | | 31 | 1.33 | 0.71 to 2.51 | 1.36 | 0.72 to 2.56 |  |
| Uterus (C54-C55) | 22 |  | 115 | 1.14 | 0.71 to 1.84 | 1.22 | 0.76 to 1.97 | |  | | 59 | 1.07 | 0.65 to 1.74 | 1.12 | 0.68 to 1.83 |  |
| Ovary (C56-C57) | 20 |  | 145 | 0.83 | 0.52 to 1.35 | 0.88 | 0.54 to 1.43 | |  | | 64 | 0.91 | 0.55 to 1.50 | 0.97 | 0.58 to 1.60 |  |
| Kidney (C64-C66) | 21 |  | 94 | 1.46 | 0.89 to 2.39 | 1.62 | 0.99 to 2.67 | |  | | 41 | 1.46 | 0.86 to 2.48 | 1.60 | 0.94 to 2.73 |  |
| Bladder (C67) | 9 |  | 51 | 0.95 | 0.46 to 1.97 | 1.01 | 0.49 to 2.11 | |  | | 27 | 0.97 | 0.46 to 2.06 | 0.99 | 0.47 to 2.13 |  |
| Brain and central nervous system (C70-C72, C75.1 and C75.3) | 7 |  | 39 | 1.08 | 0.47 to 2.50 | 1.13 | 0.49 to 2.61 | |  | | 12 | 1.67 | 0.66 to 4.27 | 1.73 | 0.68 to 4.42 |  |
| Thyroid gland (C73) | 18 |  | 88 | 1.28 | 0.75 to 2.17 | 1.36 | 0.80 to 2.32 | |  | | 50 | 1.07 | 0.63 to 1.84 | 1.15 | 0.67 to 1.97 |  |
| Cancer without specification of site (C80) | 5 |  | 63 | 0.47 | 0.19 to 1.19 | 0.51 | 0.20 to 1.29 | |  | | 28 | 0.53 | 0.21 to 1.38 | 0.57 | 0.22 to 1.48 |  |
| Lymphoid and haematopoietic tissue (LH) (C81-C96, D45-D47) | 42 |  | 166 | 1.40 | 0.98 to 1.99 | **1.52** | **1.07 to 2.17** | |  | | 81 | **1.52** | **1.04 to 2.21** | **1.55** | **1.06 to 2.25** |  |
| Hodgkin´s lymphoma (C81) | 4 |  | 15 | 1.22 | 0.39 to 3.79 | 1.29 | 0.41 to 4.01 | |  | | 5 | 2.43 | 0.64 to 9.24 | 2.44 | 0.64 to 9.29 |  |
| Non-Hodgkin´s lymphoma (NHL) (C82-C85) | 15 |  | 55 | 1.70 | 0.93 to 3.09 | **1.92** | **1.05 to 3.50** | |  | | 16 | **2.98** | **1.46 to 6.07** | **3.08** | **1.51 to 6.32** |  |
| NHL, follicular (C82) | 4 |  | 14 | 1.50 | 0.48 to 4.74 | 1.70 | 0.53 to 5.42 | |  | | 4 | 3.55 | 0.87 to 14.47 | 3.33 | 0.82 to 13.59 |  |
| NHL, diffuse (C83) | 5 |  | 31 | 1.11 | 0.42 to 2.95 | 1.28 | 0.48 to 3.44 | |  | | 11 | 1.46 | 0.50 to 4.24 | 1.58 | 0.54 to 4.63 |  |
| NHL, peripheral T-cells (C84) | 1 |  | 4 | 1.62 | 0.16 to 16.20 | 1.69 | 0.17 to 16.87 | |  | | 0 |  |  |  |  |  |
| NHL, unspecified (C85) | 5 |  | 6 | **5.24** | **1.47 to 18.61** | **5.50** | **1.55 to 19.58** | |  | | 1 | **13.72** | **1.59 to 118.27** | **14.46** | **1.66 to 125.87** |  |
| Immunoproliferative diseases (C88) | 3 |  | 12 | 1.35 | 0.37 to 4.97 | 1.49 | 0.40 to 5.55 | |  | | 6 | 1.65 | 0.41 to 6.67 | 1.89 | 0.46 to 7.76 |  |
| Multiple Myeloma (C90) | 9 |  | 32 | 1.44 | 0.67 to 3.08 | 1.53 | 0.71 to 3.29 | |  | | 26 | 0.99 | 0.46 to 2.11 | 0.97 | 0.45 to 2.08 |  |
| Leukaemia (C91-C95, D45-D47) | 11 |  | 51 | 1.18 | 0.60 to 2.31 | 1.25 | 0.63 to 2.46 | |  | | 28 | 1.16 | 0.58 to 2.33 | 1.17 | 0.58 to 2.37 |  |
| Non-CLL (C91-C95, D45-D47, except C91.1) | 7 |  | 35 | 1.09 | 0.47 to 2.53 | 1.15 | 0.49 to 2.67 | |  | | 19 | 1.11 | 0.47 to 2.65 | 1.13 | 0.47 to 2.72 |  |
| Lymphoid leukemia (C91) | 5 |  | 21 | 1.22 | 0.44 to 3.34 | 1.30 | 0.47 to 3.59 | |  | | 9 | 1.60 | 0.53 to 4.78 | 1.60 | 0.53 to 4.85 |  |
| Acute lymphoid leukemia (C91.0) | 1 |  | 4 | 1.16 | 0.12 to 11.07 | 1.21 | 0.13 to 11.59 | |  | | 0 |  |  |  |  |  |
| Chronic lymphocytic leukaemia (CLL)(C91.1) | 4 |  | 16 | 1.37 | 0.44 to 4.28 | 1.50 | 0.48 to 4.73 | |  | | 9 | 1.26 | 0.39 to 4.10 | 1.27 | 0.38 to 4.22 |  |
| Myeloid leukemia (C92) | 3 |  | 16 | 1.02 | 0.29 to 3.68 | 1.02 | 0.28 to 3.67 | |  | | 12 | 0.78 | 0.22 to 2.77 | 0.80 | 0.23 to 2.87 |  |
| Acute myeloid leukemia (C92.0) | 3 |  | 10 | 1.49 | 0.39 to 5.70 | 1.51 | 0.39 to 5.78 | |  | | 9 | 1.05 | 0.28 to 3.91 | 1.09 | 0.29 to 4.08 |  |
| Myelodysplastic syndromes (MDS) (D46) | 3 |  | 4 | **5.87** | **1.20 to 28.80** | **6.28** | **1.26 to 31.22** | |  | | 2 | 4.19 | 0.69 to 25.43 | 4.15 | 0.68 to 25.23 |  |
| MDS, unspecified (D46.9) | 3 |  | 3 | **8.57** | **1.57 to 46.62** | **9.45** | **1.72 to 51.79** | |  | | 2 | 4.19 | 0.69 to 25.43 | 4.15 | 0.68 to 25.23 |  |
| **Not included in all cancers** | | | | | | | | | | | | | | | | |
|  | p-yr 50 898 |  | p-yr 284 679 | | | | |  | | p-yr 151 910 | | | | | |  |
| Basal cell carcinoma of the skin (BCC) (C44) | 96 |  | 437 | 1.18 | 0.94 to 1.48 | 1.25 | 0.99 to 1.57 | |  | | 189 | **1.46** | **1.14 to 1.86** | **1. 55** | **1.21 to 1.99** |  |

Abbreviation: p-yr, person years.

Table I. Number of all cancers, and selected cancer sites among men and women combined in the geothermal heating areas, hazard ratio (HR), 95% confidence intervals (CI) compared with the populations in warm reference area and cold reference area, adjusted for age, gender, education, type of housing, and smoking habits, split in four categories of cumulative years of residence in the respective areas.

|  | Geothermal heating area | Warm reference area | | | Cold reference area | | |
| --- | --- | --- | --- | --- | --- | --- | --- |
|  | p-yr 97 911 | p-yr 557 815 | | | p-yr 299 878 | | |
| Cancers (ICD-10) | No of cancers | No of cancers | HR | 95%CI | No of cancers | HR | 95%CI |
| All (C00-C97 and D45-D47) |  |  |  |  |  |  |  |
| < 5 year | 181 | 800 | 0.99 | 0.84 to 1.17 | 409 | **1.20** | **1.01 to 1.43** |
| ≥ 5 years, < 15 years | 267 | 1557 | **1.15** | **1.01 to 1.32** | 806 | **1.24** | **1.07 to 1.42** |
| ≥ 15 years, < 24 years | 271 | 1503 | 1.14 | 0.99 to 1.30 | 675 | **1.22** | **1.06 to 1.41** |
| ≥ 24 years | 269 | 1471 | 1.09 | 0.95 to 1.25 | 634 | **1.20** | **1.04 to 1.38** |
| Colon, rectum, and anus (C18-C21) |  |  |  |  |  |  |  |
| < 5 year | 14 | 62 | 1.03 | 0.57 to 1.87 | 33 | 1.16 | 0.62 to 2.16 |
| ≥ 5 years, < 15 years | 21 | 136 | 1.09 | 0.68 to 1.77 | 62 | 1.31 | 0.79 to 2.17 |
| ≥ 15 years, < 24 years | 32 | 141 | 1.40 | 0.94 to 2.10 | 61 | **1.56** | **1.01 to 2.41** |
| ≥ 24 years | 23 | 134 | 1.03 | 0.65 to 1.62 | 63 | 1.05 | 0.65 to 1.69 |
| Pancreas (C25) |  |  |  |  |  |  |  |
| < 5 year | 3 | 20 | 0.66 | 0.19 to 2.28 | 11 | 0.74 | 0.21 to 3.66 |
| ≥ 5 years, < 15 years | 11 | 33 | **2.65** | **1.28 to 5.50** | 14 | **3.58** | **1.56 to 8.21** |
| ≥ 15 years, < 24 years | 11 | 35 | 1.85 | 0.90 to 3.77 | 13 | **2.58** | **1.14 to 5.85** |
| ≥ 24 years | 5 | 38 | 0.96 | 0.36 to 2.53 | 11 | 1.34 | 0.46 to 3.92 |
| Lung and bronchus (C33-C34) |  |  |  |  |  |  |  |
| < 5 year | 24 | 103 | 1.00 | 0.63 to 1.57 | 55 | 1.20 | 0.74 to 1.94 |
| ≥ 5 years, < 15 years | 23 | 167 | 1.02 | 0.65 to 1.61 | 112 | 0.76 | 0.49 to 1.20 |
| ≥ 15 years, < 24 years | 19 | 158 | 0.76 | 0.47 to 1.25 | 76 | 0.79 | 0.47 to 1.31 |
| ≥ 24 years | 26 | 150 | 0.94 | 0.61 to 1.45 | 57 | 1.32 | 0.83 to 2.10 |
| Breast (C50) |  |  |  |  |  |  |  |
| < 5 year | 37 | 123 | 1.25 | 0.86 to 1.82 | 59 | **1.61** | **1.07 to 2.44** |
| ≥ 5 years, < 15 years | 56 | 237 | **1.63** | **1.21 to 2.21** | 96 | **2.23** | **1.60 to 3.12** |
| ≥ 15 years, < 24 years | 29 | 199 | 0.92 | 0.61 to 1.37 | 95 | 1.01 | 0.67 to 1.54 |
| ≥ 24 years | 39 | 181 | 1.35 | 0.93 to 1.94 | 76 | 1.32 | 0.90 to 1.95 |
| Prostate (C61) |  |  |  |  |  |  |  |
| < 5 year | 15 | 79 | 0.84 | 0.47 to 1.49 | 33 | 1.44 | 0.76 to 2.75 |
| ≥ 5 years, < 15 years | 42 | 158 | **1.77** | **1.22 to 2.57** | 98 | **1.60** | **1.09 to 2.34** |
| ≥ 15 years, < 24 years | 61 | 267 | **1.42** | **1.06 to 1.91** | 123 | **1.50** | **1.09 to 2.05** |
| ≥ 24 years | 54 | 299 | 1.13 | 0.84 to 1.53 | 123 | 1.35 | 0.98 to 1.87 |
| Lymphoid and haematopoietic tissue (LH) (C81-C96, D45-D47) |  |  |  |  |  |  |  |
| < 5 year | 24 | 49 | **2.37** | **1.42 to 3.96** | 35 | **1.86** | **1.10 to 3.13** |
| ≥ 5 years, < 15 years | 17 | 124 | 0.76 | 0.45 to 1.27 | 67 | 1.00 | 0.58 to 1.72 |
| ≥ 15 years, < 24 years | 25 | 123 | 1.30 | 0.83 to 2.05 | 49 | **1.65** | **1.01 to 2.70** |
| ≥ 24 years | 31 | 123 | **1.57** | **1.04 to 2.37** | 48 | **1.79** | **1.13 to 2.84** |
| Non-Hodgkin´s lymphoma (NHL) (C82-C85) |  |  |  |  |  |  |  |
| < 5 year | 10 | 11 | **3.92** | **1.63 to 9.41** | 11 | **2.45** | **1.04 to 5.78** |
| ≥ 5 years, < 15 years | 8 | 39 | 1.27 | 0.57 to 2.81 | 20 | 1.64 | 0.68 to 3.97 |
| ≥ 15 years, < 24 years | 9 | 47 | 1.40 | 0.66 to 2.96 | 15 | 2.18 | 0.90 to 5.28 |
| ≥ 24 years | 12 | 40 | **2.20** | **1.11 to 4.37** | 16 | **2.22** | **1.03 to 4.78** |
| **Not included in all cancers** |  |  |  |  |  |  |  |
| Basal cell carcinoma  of the skin (C44) | p-yr 101 014 |  | p-yr 574 065 | | p-yr 307 763 | | |
|  |  |  |  |  |  |  |  |
| < 5 year | 25 | 98 | 1.07 | 0.68 to 1.67 | 61 | 1.13 | 0.71 to 1.80 |
| ≥ 5 years, < 15 years | 50 | 193 | **1.72** | **1.24 to 2.38** | 75 | **2.40** | **1.67 to 3.44** |
| ≥ 15 years, < 24 years | 46 | 197 | 1.32 | 0.95 to 1.85 | 114 | 1.27 | 0.90 to 1.79 |
| ≥ 24 years | 56 | 293 | 1.07 | 0.79 to 1.44 | 85 | **1.71** | **1.22 to 2.40** |

Abbreviation: p-yr, person years.

Table J. Number of all cancers, and selected cancer sites among men and women combined in the geothermal heating areas, hazard ratio (HR), 95% confidence intervals (CI) compared with the populations in warm reference area and cold reference area, applying five years latency time, adjusted for age, gender, education, type of housing, and smoking habits, split in four categories of cumulative years of residence in the respective areas.

|  | Geothermal heating area | Warm reference area | | | Cold reference area | | |
| --- | --- | --- | --- | --- | --- | --- | --- |
|  | p-yr 65 169 | p-yr 362 298 | | | p-yr 201 705 | | |
| Cancers (ICD-10) | No of cancers | No of cancers | HR | 95%CI | No of cancers | HR | 95%CI |
| All (C00-C97 and D45-D47) |  |  |  |  |  |  |  |
| < 5 year | 117 | 456 | 1.16 | 0.94 to 1.43 | 255 | **1.29** | **1.03 to 1.60** |
| ≥ 5 years, < 15 years | 91 | 539 | 1.15 | 0.91 to 1.45 | 321 | 1.09 | 0.86 to 1.38 |
| ≥ 15 years, < 24 years | 38 | 193 | 1.32 | 0.91 to 1.90 | 113 | 1.12 | 0.77 to 1.62 |
| ≥ 24 years | 126 | 657 | 1.11 | 0.91 to 1.36 | 270 | **1.32** | **1.06 to 1.63** |
| Colon, rectum, and anus (C18-C21) |  |  |  |  |  |  |  |
| < 5 year | 11 | 38 | 1.33 | 0.67 to 2.65 | 24 | 1.29 | 0.63 to 2.64 |
| ≥ 5 years, < 15 years | 7 | 49 | 1.17 | 0.51 to 2.68 | 23 | 1.27 | 0.53 to 3.01 |
| ≥ 15 years, < 24 years | 1 | 15 | 0.38 | 0.05 to 2.97 | 7 | 0.60 | 0.07 to 4.92 |
| ≥ 24 years | 13 | 61 | 1.20 | 0.64 to 2.23 | 24 | 1.53 | 0.78 to 3.02 |
| Pancreas (C25) |  |  |  |  |  |  |  |
| < 5 year | 1 | 8 | 0.55 | 0.07 to 4.47 | 6 | 0.47 | 0.06 to 3.91 |
| ≥ 5 years, < 15 years | 7 | 11 | **5.18** | **1.85 to 14.53** | 7 | **4.90** | **1.65 to 14.53** |
| ≥ 15 years, < 24 years | 1 | 3 | 2.47 | 0.22 to 27.76 | 2 | 2.68 | 0.23 to 31.74 |
| ≥ 24 years | 2 | 14 | 1.25 | 0.27 to 5.89 | 4 | 1.71 | 0.31 to 9.52 |
| Lung and bronchus (C33-C34) |  |  |  |  |  |  |  |
| < 5 year | 19 | 73 | 1.10 | 0.66 to 1.85 | 38 | 1.41 | 0.81 to 2.45 |
| ≥ 5 years, < 15 years | 4 | 60 | 0.49 | 0.18 to 1.38 | 48 | **0.35** | **0.12 to 0.96** |
| ≥ 15 years, < 24 years | 7 | 20 | 2.45 | 0.96 to 6.23 | 14 | 1.56 | 0.62 to 3.97 |
| ≥ 24 years | 10 | 56 | 0.91 | 0.46 to 1.82 | 26 | 1.06 | 0.51 to 2.20 |
| Breast (C50) |  |  |  |  |  |  |  |
| < 5 year | 19 | 63 | 1.29 | 0.76 to 2.17 | 34 | 1.47 | 0.84 to 2.59 |
| ≥ 5 years, < 15 years | 17 | 81 | 1.38 | 0.80 to 2.37 | 44 | 1.57 | 0.90 to 2.77 |
| ≥ 15 years, < 24 years | 3 | 33 | 0.62 | 0.19 to 2.09 | 19 | 0.62 | 0.18 to 2.12 |
| ≥ 24 years | 17 | 100 | 1.07 | 0.63 to 1.84 | 36 | 1.26 | 0.70 to 2.25 |
| Prostate (C61) |  |  |  |  |  |  |  |
| < 5 year | 13 | 68 | 0.88 | 0.47 to 1.64 | 30 | 1.41 | 0.71 to 2.80 |
| ≥ 5 years, < 15 years | 11 | 60 | 1.27 | 0.64 to 2.52 | 42 | 1.00 | 0.50 to 2.00 |
| ≥ 15 years, < 24 years | 9 | 34 | 2.10 | 0.93 to 4.73 | 20 | 1.49 | 0.66 to 3.33 |
| ≥ 24 years | 24 | 126 | 1.18 | 0.75 to 1.86 | 46 | 1.60 | 0.97 to 2.64 |
| Lymphoid and haematopoietic tissue (LH) (C81-C96, D45-D47) |  |  |  |  |  |  |  |
| < 5 year | 14 | 27 | **2.62** | **1.33 to 5.14** | 18 | **2.21** | **1.09 to 4.46** |
| ≥ 5 years, < 15 years | 5 | 44 | 0.74 | 0.29 to 1.92 | 31 | 0.62 | 0.24 to 1.60 |
| ≥ 15 years, < 24 years | 2 | 22 | 0.65 | 0.15 to 2.87 | 7 | 0.96 | 0.19 to 4.72 |
| ≥ 24 years | 16 | 50 | **1.91** | **1.05 to 3.47** | 16 | **2.93** | **1.44 to 5.97** |
| Non-Hodgkin´s lymphoma (NHL) (C82-C85) |  |  |  |  |  |  |  |
| < 5 year | 7 | 8 | **4.02** | **1.40 to 11.51** | 7 | 2.86 | 0.99 to 8.26 |
| ≥ 5 years, < 15 years | 2 | 15 | 1.05 | 0.23 to 4.85 | 8 | 1.05 | 0.20 to 5.43 |
| ≥ 15 years, < 24 years | 2 | 8 | 2.23 | 0.43 to 11.71 | 2 | 8.72 | 0.78 to 98.12 |
| ≥ 24 years | 6 | 18 | 2.03 | 0.76 to 5.43 | 3 | **6.15** | **1.42 to 26.61** |
| **Not included in all cancers** |  |  |  |  |  |  |  |
| Basal cell carcinoma of the skin (C44) | p-yr 67 151 |  | p-yr 371 689 | | p-yr 206 015 | | |
|  |  |  |  |  |  |  |  |
| < 5 year | 18 | 70 | 1.09 | 0.64 to 1.86 | 47 | 1.09 | 0.63 to 1.87 |
| ≥ 5 years, < 15 years | 20 | 95 | 1.30 | 0.79 to 2.14 | 43 | 1.68 | 0.98 to 2.88 |
| ≥ 15 years, < 24 years | 8 | 50 | 0.80 | 0.37 to 1.72 | 29 | 0.86 | 0.39 to 1.90 |
| ≥ 24 years | 28 | 142 | 1.06 | 0.70 to 1.61 | 35 | **2.11** | **1.28 to 2.48** |

Abbreviation: p-yr, person years.

Table K. Number of all cancers, and selected cancer sites among men and women combined, hazard ratio (HR), 95% confidence intervals (CI) compared with the populations in warm reference area and cold reference area, adjusted for age, gender, education, type of housing, and smoking habits, without and with stratification into categories of cumulative years of residence, restricted on different age categories in the respective areas.

|  | Geothermal heating area |  | Warm reference area | | | | |  | | Cold reference area | | | | | |
| --- | --- | --- | --- | --- | --- | --- | --- | --- | --- | --- | --- | --- | --- | --- | --- |
|  | No of |  | No of | Not stratified | | Stratified | | |  | | No of | Not stratified | | Stratified | |
| Cancers (ICD-10) | cancers |  | cancers | HR | 95%CI | HR | 95%CI | |  | | cancers | HR | 95%CI | HR | 95%CI |
| All (C00-C97, D45-D47) |  |  |  |  |  |  |  | |  | |  |  |  |  |  |
| < 20 | 63 |  | 348 | 1.00 | 0.76 to 1.32 | 1.06 | 0.80 to 1.40 | |  | | 165 | 1.13 | 0.85 to 1.52 | 1.18 | 0.88 to 1.57 |
| < 25 | 109 |  | 582 | 1.13 | 0.92 to 1.40 | 1.21 | 0.97 to 1.49 | |  | | 275 | **1.29** | **1.03 to 1.62** | **1.33** | **1.07 to 1.67** |
| < 30 | 164 |  | 951 | 1.06 | 0.89 to 1.26 | 1.13 | 0.95 to 1.35 | |  | | 452 | 1.19 | 0.99 to 1.42 | **1.24** | **1.04 to 1.49** |
| < 35 | 259 |  | 1433 | 1.06 | 0.93 to 1.22 | 1.12 | 0.97 to 1.28 | |  | | 658 | **1.26** | **1.09 to 1.45** | **1.31** | **1.13 to 1.52** |
| < 40 | 357 |  | 2022 | 1.00 | 0.89 to 1.12 | 1.06 | 0.94 to 1.19 | |  | | 937 | **1.16** | **1.02 to 1.31** | **1.21** | **1.07 to 1.37** |
| ≥ 40 | 631 |  | 3309 | **1.11** | **1.01 to 1.21** | **1.13** | **1.04 to 1.24** | |  | | 1587 | **1.16** | **1.06 to 1.27** | **1.19** | **1.09 to 1.31** |
| Colon, rectum, and anus (C18-C21) |  |  |  |  |  |  |  | |  | |  |  |  |  |  |
| < 20 | 4 |  | 17 | 1.49 | 0.47 to 4.74 | 1.55 | 0.48 to 4.94 | |  | | 6 | 1.90 | 0.54 to 6.76 | 2.00 | 0.56 to 7.14 |
| < 25 | 8 |  | 33 | 1.64 | 0.73 to 3.72 | 1.74 | 0.77 to 3.94 | |  | | 9 | **3.15** | **1.20 to 8.24** | **3.31** | **1.26 to 8.68** |
| < 30 | 12 |  | 53 | 1.60 | 0.83 to 3.10 | 1.74 | 0.89 to 3.38 | |  | | 20 | **2.05** | **1.00 to 4.21** | **2.22** | **1.08 to 4.59** |
| < 35 | 19 |  | 86 | 1.39 | 0.83 to 2.34 | 1.50 | 0.89 to 2.54 | |  | | 37 | 1.69 | 0.97 to 2.94 | **1.79** | **1.02 to 3.12** |
| < 40 | 26 |  | 125 | 1.30 | 0.83 to 2.02 | 1.44 | 0.92 to 2.25 | |  | | 66 | 1.23 | 0.78 to 1.94 | 1.32 | 0.83 to 2.09 |
| ≥ 40 | 64 |  | 348 | 1.07 | 0.81 to 1.41 | 1.09 | 0.83 to 1.44 | |  | | 153 | 1.19 | 0.89 to 1.59 | 1.19 | 0.89 to 1.60 |
| Pancreas (C25) |  |  |  |  |  |  |  | |  | |  |  |  |  |  |
| < 20 | 0 |  | 1 |  |  |  |  | |  | | 2 |  |  |  |  |
| < 25 | 0 |  | 2 |  |  |  |  | |  | | 5 |  |  |  |  |
| < 30 | 1 |  | 16 | 0.42 | 0.05 to 3.26 | 0.45 | 0.06 to 3.52 | |  | | 5 | 0.64 | 0.07 to 5.55 | 0.62 | 0.07 to 5.40 |
| < 35 | 3 |  | 25 | 0.78 | 0.23 to 2.66 | 0.83 | 0.24 to 2.86 | |  | | 9 | 1.13 | 0.31 to 4.20 | 1.19 | 0.32 to 4.43 |
| < 40 | 5 |  | 36 | 0.93 | 0.35 to 2.45 | 1.00 | 0.38 to 2.66 | |  | | 16 | 1.02 | 0.37 to 2.80 | 1.06 | 0.39 to 2.92 |
| ≥ 40 | 25 |  | 90 | **1.73** | **1.08 to 2.74** | **1.72** | **1.08 to 2.74** | |  | | 33 | **2.21** | **1.31 to 3.73** | **2.28** | **1.34 to 3.86** |
| Breast (C50) |  |  |  |  |  |  |  | |  | |  |  |  |  |  |
| < 20 | 16 |  | 62 | 1.29 | 0.73 to 2.29 | 1.49 | 0.83 to 2.65 | |  | | 33 | 1.41 | 0.78 to 2.57 | 1.55 | 0.85 to 2.84 |
| < 25 | 29 |  | 117 | 1.49 | 0.98 to 2.29 | **1.70** | **1.11 to 2.62** | |  | | 64 | 1.51 | 0.97 to 2.35 | **1.58** | **1.02 to 2.46** |
| < 30 | 41 |  | 180 | 1.36 | 0.95 to 1.94 | **1.50** | **1.05 to 2.14** | |  | | 110 | 1.19 | 0.83 to 1.71 | 1.28 | 0.89 to 1.84 |
| < 35 | 61 |  | 272 | 1.25 | 0.93 to 1.67 | **1.35** | **1.01 to 1.80** | |  | | 144 | 1.30 | 0.96 to 1.76 | **1.37** | **1.01 to 1.85** |
| < 40 | 84 |  | 397 | 1.15 | 0.90 to 1.47 | 1.24 | 0.97 to 1.59 | |  | | 179 | **1.37** | **1.06 to 1.78** | **1.46** | **1.13 to 1.90** |
| ≥ 40 | 77 |  | 343 | **1.32** | **1.02 to 1.70** | **1.32** | **1.02 to 1.71** | |  | | 147 | **1.44** | **1.09 to 1.90** | **1.48** | **1.12 to 1.95** |
| Prostate (C61) |  |  |  |  |  |  |  | |  | |  |  |  |  |  |
| < 20 | 4 |  | 7 | 2.85 | 0.76 to 10.65 | 3.28 | 0.87 to 12.42 | |  | | 3 | 3.97 | 0.85 to 18.56 | 4.22 | 0.90 to 19.67 |
| < 25 | 5 |  | 18 | 1.49 | 0.53 to 4.20 | 1.57 | 0.55 to 4.44 | |  | | 9 | 1.86 | 0.61 to 5.67 | 1.92 | 0.63 to 5.87 |
| < 30 | 10 |  | 39 | 1.47 | 0.71 to 3.04 | 1.57 | 0.76 to 3.25 | |  | | 25 | 1.37 | 0.65 to 2.88 | 1.44 | 0.68 to 3.04 |
| < 35 | 17 |  | 111 | 0.89 | 0.52 to 1.52 | 0.96 | 0.56 to 1.64 | |  | | 56 | 1.02 | 0.59 to 1.78 | 1.09 | 0.63 to 1.91 |
| < 40 | 34 |  | 209 | 0.87 | 0.60 to 1.27 | 0.97 | 0.66 to 1.41 | |  | | 100 | 1.12 | 0.75 to 1.67 | 1.17 | 0.78 to 1.75 |
| ≥ 40 | 138 |  | 594 | **1.45** | **1.19 to 1.76** | **1.47** | **1.21 to 1.79** | |  | | 277 | **1.53** | **1.24 to 1.88** | **1.56** | **1.27 to 1.92** |
| Kidney (C64-C66) |  |  |  |  |  |  |  | |  | |  |  |  |  |  |
| < 20 | 2 |  | 15 | 0.81 | 0.17 to 3.73 | 0.90 | 0.19 to 4.16 | |  | | 5 | 1.68 | 0.29 to 9.84 | 1.87 | 0.31 to 11.18 |
| < 25 | 2 |  | 22 | 0.59 | 0.13 to 2.62 | 0.65 | 0.15 to 2.87 | |  | | 11 | 0.62 | 0.14 to 2.79 | 0.62 | 0.14 to 2.83 |
| < 30 | 4 |  | 42 | 0.59 | 0.20 to 1.68 | 0.60 | 0.21 to 1.73 | |  | | 23 | 0.59 | 0.20 to 1.69 | 0.60 | 0.21 to 1.73 |
| < 35 | 13 |  | 61 | 1.21 | 0.65 to 2.25 | 1.23 | 0.66 to 2.31 | |  | | 29 | 1.45 | 0.75 to 2.79 | 1.53 | 0.79 to 2.95 |
| < 40 | 18 |  | 87 | 1.12 | 0.66 to 1.90 | 1.18 | 0.69 to 2.00 | |  | | 41 | 1.32 | 0.76 to 2.30 | 1.39 | 0.80 to 2.43 |
| ≥ 40 | 31 |  | 154 | 1.28 | 0.86 to 1.91 | 1.35 | 0.90 to 2.03 | |  | | 62 | 1.45 | 0.94 to 2.24 | 1.54 | 0.99 to 2.39 |
| Lymphoid and haematopoietic tissue (LH) (C81-C96, D45-D47) |  |  |  |  |  |  |  | |  | |  |  |  |  |  |
| < 20 | 6 |  | 32 | 1.03 | 0.42 to 2.55 | 1.06 | 0.43 to 2.62 | |  | | 21 | 0.98 | 0.38 to 2.49 | 0.99 | 0.39 to 2.53 |
| < 25 | 13 |  | 49 | 1.49 | 0.78 to 2.81 | 1.57 | 0.83 to 2.97 | |  | | 37 | 1.20 | 0.63 to 2.28 | 1.21 | 0.63 to 2.31 |
| < 30 | 19 |  | 98 | 1.21 | 0.73 to 2.02 | 1.29 | 0.77 to 2.16 | |  | | 48 | 1.43 | 0.83 to 2.47 | 1.45 | 0.84 to 2.51 |
| < 35 | 28 |  | 141 | 1.21 | 0.79 to 1.85 | 1.27 | 0.83 to 1.94 | |  | | 62 | **1.62** | **1.02 to 2.57** | **1.66** | **1.05 to 2.64** |
| < 40 | 33 |  | 183 | 1.05 | 0.71 to 1.54 | 1.12 | 0.76 to 1.64 | |  | | 86 | 1.21 | 0.81 to 1.81 | 1.25 | 0.83 to 1.87 |
| ≥ 40 | 64 |  | 236 | **1.50** | **1.13 to 2.00** | **1.56** | **1.17 to 2.09** | |  | | 113 | **1.70** | **1.25 to 2.32** | **1.78** | **1.30 to 2.43** |
| Non-Hodgkin´s lymphoma (NHL) (C82-C85) |  |  |  |  |  |  |  | |  | |  |  |  |  |  |
| < 20 | 3 |  | 7 | 2.64 | 0.61 to 11.35 | 2.90 | 0.67 to 12.57 | |  | | 6 | 1.87 | 0.43 to 8.17 | 1.96 | 0.44 to 8.63 |
| < 25 | 6 |  | 14 | **2.97** | **1.06 to 8.30** | **3.32** | **1.18 to 9.30** | |  | | 11 | 2.33 | 0.79 to 6.84 | 2.33 | 0.80 to 6.83 |
| < 30 | 11 |  | 33 | **2.31** | **1.12 to 4.78** | **2.58** | **1.24 to 5.37** | |  | | 19 | **2.46** | **1.11 to 5.42** | **2.57** | **1.16 to 5.69** |
| < 35 | 16 |  | 48 | **2.26** | **1.23 to 4.14** | **2.48** | **1.34 to 4.56** | |  | | 22 | **2.67** | **1.36 to 5.22** | **2.80** | **1.42 to 5.51** |
| < 40 | 18 |  | 60 | **1.92** | **1.09 to 3.36** | **2.14** | **1.22 to 3.75** | |  | | 31 | **1.93** | **1.06 to 3.50** | **2.00** | **1.10 to 3.64** |
| ≥ 40 | 21 |  | 77 | **1.66** | **1.00 to 2.75** | **1.74** | **1.05 to 2.89** | |  | | 31 | **2.08** | **1.17 to 3.68** | **2.22** | **1.24 to 3.94** |
| **Not included in all cancers** | | | | | | | | | | | | | | | |
| Basal cell carcinoma of the skin (C44) |  |  |  |  |  |  |  | |  | |  |  |  |  |  |
| < 20 | 14 |  | 70 | 1.02 | 0.56 to 1.84 | 1.12 | 0.61 to 2.03 | |  | | 35 | 1.14 | 0.61 to 2.11 | 1.25 | 0.67 to 2.33 |
| < 25 | 25 |  | 113 | 1.18 | 0.75 to 1.85 | 1.29 | 0.82 to 2.02 | |  | | 50 | 1.54 | 0.95 to 2.49 | **1.69** | **1.04 to 2.75** |
| < 30 | 42 |  | 163 | 1.38 | 0.97 to 1.96 | **1.50** | **1.05 to 2.14** | |  | | 75 | **1.72** | **1.18 to 2.52** | **1.84** | **1.26 to 2.70** |
| < 35 | 52 |  | 234 | 1.17 | 0.86 to 1.61 | 1.28 | 0.93 to 1.74 | |  | | 102 | **1.56** | **1.11 to 2.18** | **1.66** | **1.19 to 2.33** |
| < 40 | 76 |  | 314 | 1.25 | 0.97 to 1.63 | **1.36** | **1.04 to 1.76** | |  | | 138 | **1.60** | **1.21 to 2.12** | **1.71** | **1.29 to 2.27** |
| ≥ 40 | 101 |  | 467 | 1.20 | 0.96 to 1.50 | 1.24 | 0.99 to 1.55 | |  | | 197 | **1.47** | **1.16 to 1.87** | **1.54** | **1.21 to 1.96** |

Table L. Number of all cancers, and selected cancer sites among men in the geothermal heating areas, hazard ratio (HR), 95% confidence intervals (CI) compared with the populations in warm reference area and cold reference area, adjusted for age, gender, education, type of housing, and smoking habits, without and with stratification into categories of cumulative years of residence, restricted on different age categories in the respective areas.

|  | Geothermal heating area |  | Warm reference area | | | | |  | | Cold reference area | | | | | |
| --- | --- | --- | --- | --- | --- | --- | --- | --- | --- | --- | --- | --- | --- | --- | --- |
|  | No of |  | No of | Not stratified | | Stratified | | |  | | No of | Not stratified | | Stratified | |
| Cancers (ICD-10) | cancers |  | cancers | HR | 95%CI | HR | 95%CI | |  | | cancers | HR | 95%CI | HR | 95%CI |
| All (C00-C97, D45-D47) |  |  |  |  |  |  |  | |  | |  |  |  |  |  |
| < 20 | 19 |  | 133 | 0.86 | 0.52 to 1.42 | 0.87 | 0.53 to 1.44 | |  | | 57 | 1.00 | 0.59 to 1.68 | 1.01 | 0.60 to 1.71 |
| < 25 | 32 |  | 228 | 0.86 | 0.59 to 1.26 | 0.88 | 0.60 to 1.29 | |  | | 93 | 1.10 | 0.74 to 1.65 | 1.15 | 0.76 to 1.72 |
| < 30 | 48 |  | 386 | 0.77 | 0.57 to 1.05 | 0.80 | 0.59 to 1.09 | |  | | 166 | 0.98 | 0.71 to 1.35 | 1.02 | 0.74 to 1.41 |
| < 35 | 91 |  | 628 | 0.85 | 0.68 to 1.07 | 0.88 | 0.70 to 1.11 | |  | | 276 | 1.09 | 0.86 to 1.39 | 1.15 | 0.91 to 1.46 |
| < 40 | 147 |  | 929 | 0.91 | 0.76 to 1.09 | 0.95 | 0.79 to 1.14 | |  | | 422 | 1.12 | 0.93 to 1.35 | 1.17 | 0.97 to 1.42 |
| ≥ 40 | 370 |  | 1910 | **1.12** | **1.00 to 1.26** | **1.13** | **1.01 to 1.27** | |  | | 924 | **1.20** | **1.06 to 1.35** | **1.22** | **1.08 to 1.38** |
| Colon, rectum, and anus (C18-C21) |  |  |  |  |  |  |  | |  | |  |  |  |  |  |
| < 20 | 2 |  | 10 | 1.26 | 0.25 to 6.40 | 1.32 | 0.26 to 6.72 | |  | | 2 | 3.11 | 0.41 to 23.75 | 3.66 | 0.48 to 28.07 |
| < 25 | 5 |  | 19 | 1.85 | 0.64 to 5.32 | 1.90 | 0.66 to 5.46 | |  | | 3 | **7.44** | **1.52 to 36.38** | **7.95** | **1.64 to 38.61** |
| < 30 | 5 |  | 32 | 1.08 | 0.40 to 2.89 | 1.13 | 0.42 to 3.04 | |  | | 9 | 2.00 | 0.66 to 6.02 | 2.20 | 0.73 to 6.66 |
| < 35 | 11 |  | 54 | 1.26 | 0.64 to 2.48 | 1.36 | 0.69 to 2.69 | |  | | 21 | 1.78 | 0.85 to 3.69 | 1.84 | 0.88 to 3.82 |
| < 40 | 17 |  | 79 | 1.36 | 0.78 to 2.36 | 1.50 | 0.86 to 2.61 | |  | | 38 | 1.46 | 0.82 to 2.60 | 1.56 | 0.87 to 2.79 |
| ≥ 40 | 38 |  | 190 | 1.17 | 0.81 to 1.68 | 1.16 | 0.81 to 1.68 | |  | | 91 | 1.19 | 0.81 to 1.74 | 1.16 | 0.79 to 1.70 |
| Pancreas (C25) |  |  |  |  |  |  |  | |  | |  |  |  |  |  |
| < 20 | 0 |  | 0 |  |  |  |  | |  | | 1 |  |  |  |  |
| < 25 | 0 |  | 1 |  |  |  |  | |  | | 2 |  |  |  |  |
| < 30 | 1 |  | 8 | 0.74 | 0.09 to 6.22 | 0.76 | 0.09 to 6.35 | |  | | 2 | 1.65 | 0.13 to 20.47 | 1.90 | 0.15 to 24.00 |
| < 35 | 2 |  | 11 | 0.95 | 0.20 to 4.47 | 0.96 | 0.20 to 4.54 | |  | | 5 | 1.66 | 0.29 to 9.35 | 1.83 | 0.31 to 10.67 |
| < 40 | 4 |  | 19 | 1.28 | 0.41 to 3.98 | 1.36 | 0.44 to 4.23 | |  | | 8 | 1.86 | 0.55 to 6.31 | 1.98 | 0.58 to 6.81 |
| ≥ 40 | 14 |  | 48 | 1.78 | 0.95 to 3.34 | 1.76 | 0.94 to 3.32 | |  | | 14 | **2.95** | **1.39 to 6.26** | **2.98** | **1.40 to 6.36** |
| Breast (C50) |  |  |  |  |  |  |  | |  | |  |  |  |  |  |
| < 20 | 0 |  | 0 |  |  |  |  | |  | | 0 |  |  |  |  |
| < 25 | 0 |  | 0 |  |  |  |  | |  | | 1 |  |  |  |  |
| < 30 | 1 |  | 0 |  |  |  |  | |  | | 2 | 1.81 | 0.15 to 22.16 | 2.04 | 0.16 to 25.93 |
| < 35 | 2 |  | 2 | 4.33 | 0.53 to 35.55 | 4.30 | 0.52 to 35.25 | |  | | 2 | 3.98 | 0.50 to 31.72 | 4.67 | 0.57 to 38.60 |
| < 40 | 3 |  | 2 | 6.32 | 0.93 to 42.95 | **7.05** | **1.03 to 48.12** | |  | | 2 | 5.38 | 0.82 to 35.18 | 6.21 | 0.91 to 42.24 |
| ≥ 40 | 0 |  | 7 |  |  |  |  | |  | | 3 |  |  |  |  |
| Prostate (C61) |  |  |  |  |  |  |  | |  | |  |  |  |  |  |
| < 20 | 4 |  | 7 | 2.85 | 0.76 to 10.65 | 3.28 | 0.87 to 12.42 | |  | | 3 | 3.97 | 0.85 to 18.56 | 4.22 | 0.90 to 19.67 |
| < 25 | 5 |  | 18 | 1.49 | 0.53 to 4.20 | 1.57 | 0.55 to 4.44 | |  | | 9 | 1.86 | 0.61 to 5.67 | 1.92 | 0.63 to 5.87 |
| < 30 | 10 |  | 39 | 1.47 | 0.71 to 3.04 | 1.57 | 0.76 to 3.25 | |  | | 25 | 1.37 | 0.65 to 2.88 | 1.44 | 0.68 to 3.04 |
| < 35 | 17 |  | 111 | 0.89 | 0.52 to 1.52 | 0.96 | 0.56 to 1.64 | |  | | 56 | 1.02 | 0.59 to 1.78 | 1.09 | 0.63 to 1.91 |
| < 40 | 34 |  | 209 | 0.87 | 0.60 to 1.27 | 0.97 | 0.66 to 1.41 | |  | | 100 | 1.12 | 0.75 to 1.67 | 1.17 | 0.78 to 1.75 |
| ≥ 40 | 138 |  | 594 | **1.45** | **1.19 to 1.76** | **1.47** | **1.21 to 1.79** | |  | | 277 | **1.53** | **1.24 to 1.88** | **1.56** | **1.27 to 1.92** |
| Kidney (C64-C66) |  |  |  |  |  |  |  | |  | |  |  |  |  |  |
| < 20 | 1 |  | 12 | 0.55 | 0.07 to 4.48 | 0.62 | 0.08 to 5.06 | |  | | 4 | 1.05 | 0.10 to 11.72 | 1.13 | 0.10 to 12.63 |
| < 25 | 1 |  | 15 | 0.41 | 0.05 to 3.20 | 0.45 | 0.06 to 3.54 | |  | | 6 | 0.53 | 0.06 to 4.42 | 0.58 | 0.07 to 4.81 |
| < 30 | 1 |  | 28 | 0.21 | 0.03 to 1.56 | 0.22 | 0.03 to 1.62 | |  | | 13 | 0.25 | 0.03 to 1.92 | 0.26 | 0.03 to 2.00 |
| < 35 | 6 |  | 37 | 0.89 | 0.36 to 2.18 | 0.88 | 0.36 to 2.16 | |  | | 17 | 1.14 | 0.45 to 2.89 | 1.21 | 0.48 to 3.07 |
| < 40 | 11 |  | 55 | 1.05 | 0.53 to 2.06 | 1.09 | 0.55 to 2.14 | |  | | 24 | 1.39 | 0.68 to 2.84 | 1.48 | 0.72 to 3.04 |
| ≥ 40 | 17 |  | 92 | 1.07 | 0.63 to 1.84 | 1.09 | 0.64 to 1.88 | |  | | 38 | 1.35 | 0.76 to 2.41 | 1.37 | 0.77 to 2.45 |
| Lymphoid and haematopoietic tissue (LH) (C81-C96, D45-D47) |  |  |  |  |  |  |  | |  | |  |  |  |  |  |
| < 20 | 2 |  | 19 | 0.67 | 0.15 to 3.03 | 0.67 | 0.15 to 3.03 | |  | | 14 | 0.43 | 0.10 to 1.92 | 0.43 | 0.10 to 1.92 |
| < 25 | 4 |  | 29 | 0.76 | 0.26 to 2.23 | 0.80 | 0.27 to 2.33 | |  | | 22 | 0.60 | 0.20 to 1.76 | 0.62 | 0.21 to 1.84 |
| < 30 | 6 |  | 56 | 0.68 | 0.29 to 1.63 | 0.72 | 0.30 to 1.72 | |  | | 31 | 0.68 | 0.28 to 1.65 | 0.70 | 0.29 to 1.71 |
| < 35 | 11 |  | 87 | 0.78 | 0.41 to 1.49 | 0.81 | 0.42 to 1.54 | |  | | 41 | 0.96 | 0.48 to 1.91 | 1.00 | 0.50 to 1.99 |
| < 40 | 14 |  | 115 | 0.71 | 0.40 to 1.25 | 0.74 | 0.42 to 1.31 | |  | | 57 | 0.78 | 0.44 to 1.41 | 0.82 | 0.46 to 1.48 |
| ≥ 40 | 41 |  | 138 | **1.68** | **1.17 to 2.43** | **1.71** | **1.18 to 2.48** | |  | | 61 | **2.19** | **1.44 to 3.31** | **2.27** | **1.49 to 3.46** |
| Non-Hodgkin´s lymphoma  (NHL) (C82-C85) |  |  |  |  |  |  |  | |  | |  |  |  |  |  |
| < 20 | 2 |  | 6 | 2.23 | 0.40 to 12.59 | 2.47 | 0.44 to 13.94 | |  | | 4 | 2.07 | 0.29 to 14.68 | 2.21 | 0.30 to 16.39 |
| < 25 | 3 |  | 9 | 2.11 | 0.53 to 8.43 | 2.46 | 0.61 to 9.89 | |  | | 9 | 1.20 | 0.30 to 4.77 | 1.26 | 0.32 to 5.02 |
| < 30 | 4 |  | 20 | 1.48 | 0.48 to 4.60 | 1.68 | 0.54 to 5.24 | |  | | 15 | 0.99 | 0.31 to 3.08 | 1.03 | 0.33 to 3.25 |
| < 35 | 7 |  | 32 | 1.53 | 0.64 to 3.65 | 1.71 | 0.71 to 4.11 | |  | | 18 | 1.32 | 0.54 to 3.20 | 1.40 | 0.57 to 3.42 |
| < 40 | 9 |  | 40 | 1.46 | 0.68 to 3.14 | 1.62 | 0.75 to 3.51 | |  | | 25 | 1.14 | 0.53 to 2.46 | 1.17 | 0.54 to 2.53 |
| ≥ 40 | 15 |  | 42 | **2.16** | **1.15 to 4.04** | **2.14** | **1.14 to 4.02** | |  | | 21 | **2.22** | **1.10 to 4.51** | **2.31** | **1.13 to 4.70** |
| **Not included in all cancers** | | | | | | | | | | | | | | | |
| Basal cell carcinoma of the skin (C44) |  |  |  |  |  |  |  | |  | |  |  |  |  |  |
| < 20 | 6 |  | 23 | 1.38 | 0.53 to 3.57 | 1.45 | 0.56 to 3.78 | |  | | 11 | 1.62 | 0.60 to 4.41 | 1.61 | 0.59 to 4.41 |
| < 25 | 10 |  | 45 | 1.36 | 0.66 to 2.81 | 1.45 | 0.70 to 2.99 | |  | | 15 | 2.20 | 0.97 to 4.98 | **2.28** | **1.00 to 5.20** |
| < 30 | 17 |  | 66 | 1.63 | 0.93 to 2.88 | **1.80** | **1.02 to 3.16** | |  | | 26 | **2.11** | **1.14 to 3.90** | **2.18** | **1.18 to 4.03** |
| < 35 | 23 |  | 102 | 1.27 | 0.79 to 2.03 | 1.39 | 0.87 to 2.24 | |  | | 36 | **2.02** | **1.20 to 3.42** | **2.14** | **1.26 to 3.62** |
| < 40 | 34 |  | 133 | 1.38 | 0.93 to 2.05 | **1.51** | **1.02 to 2.24** | |  | | 47 | **2.22** | **1.42 to 3.45** | **2.32** | **1.49 to 3.62** |
| ≥ 40 | 47 |  | 211 | 1.21 | 0.87 to 1.68 | 1.24 | 0.89 to 1.72 | |  | | 99 | 1.38 | 0.97 to 1.95 | 1.41 | 0.99 to 2.01 |

Table M. Number of selected cancer sites among women in the geothermal heating areas, hazard ratio (HR), 95% confidence intervals (CI) compared with the populations in warm reference area and cold reference area, adjusted for age, gender, education, type of housing, and smoking habits, without and with stratification into categories of cumulative years of residence, restricted on different age categories in the respective areas.

|  | Geothermal heating area |  | Warm reference area | | | | |  | | Cold reference area | | | | | |
| --- | --- | --- | --- | --- | --- | --- | --- | --- | --- | --- | --- | --- | --- | --- | --- |
|  | No of |  | No of | Not stratified | | Stratified | | |  | | No of | Not stratified | | Stratified | |
| Cancers (ICD-10) | cancers |  | cancers | HR | 95%CI | HR | 95%CI | |  | | cancers | HR | 95%CI | HR | 95%CI |
| All (C00-C97, D45-D47) |  |  |  |  |  |  |  | |  | |  |  |  |  |  |
| < 20 | 44 |  | 215 | 1.09 | 0.77 to 1.52 | 1.20 | 0.85 to 1.68 | |  | | 108 | 1.20 | 0.84 to 1.70 | 1.26 | 0.88 to 1.79 |
| < 25 | 77 |  | 354 | **1.32** | **1.02 to 1.70** | **1.45** | **1.12 to 1.88** | |  | | 182 | **1.39** | **1.06 to 1.82** | **1.43** | **1.09 to 1.87** |
| < 30 | 116 |  | 565 | **1.27** | **1.03 to 1.56** | **1.38** | **1.12 to 1.70** | |  | | 286 | **1.31** | **1.05 to 1.62** | **1.37** | **1.10 to 1.70** |
| < 35 | 168 |  | 805 | **1.22** | **1.03 to 1.46** | **1.31** | **1.10 to 1.56** | |  | | 382 | **1.37** | **1.15 to 1.65** | **1.43** | **1.19 to 1.72** |
| < 40 | 210 |  | 1093 | 1.08 | 0.93 to 1.26 | **1.17** | **1.00 to 1.36** | |  | | 515 | **1.20** | **1.02 to 1.41** | **1.26** | **1.08 to 1.49** |
| ≥ 40 | 261 |  | 1399 | 1.09 | 0.95 to 1.25 | 1.13 | 0.99 to 1.30 | |  | | 663 | 1.11 | 0.96 to 1.28 | **1.16** | **1.01 to 1.35** |
| Colon, rectum, and anus (C18-C21) |  |  |  |  |  |  |  | |  | |  |  |  |  |  |
| < 20 | 2 |  | 7 | 1.82 | 0.35 to 9.55 | 2.03 | 0.38 to 10.81 | |  | | 4 | 1.45 | 0.27 to 7.99 | 1.56 | 0.28 to 8.73 |
| < 25 | 3 |  | 14 | 1.43 | 0.39 to 5.26 | 1.70 | 0.46 to 6.29 | |  | | 6 | 1.74 | 0.43 to 7.06 | 1.77 | 0.44 to 7.19 |
| < 30 | 7 |  | 21 | 2.44 | 0.98 to 6.05 | **2.78** | **1.11 to 6.92** | |  | | 11 | 2.24 | 0.86 to 5.84 | 2.42 | 0.92 to 6.39 |
| < 35 | 8 |  | 32 | 1.61 | 0.71 to 3.65 | 1.75 | 0.77 to 3.98 | |  | | 16 | 1.67 | 0.71 to 3.93 | 1.81 | 0.77 to 4.29 |
| < 40 | 9 |  | 46 | 1.19 | 0.56 to 2.51 | 1.32 | 0.62 to 2.79 | |  | | 28 | 0.99 | 0.46 to 2.10 | 1.05 | 0.49 to 2.25 |
| ≥ 40 | 26 |  | 158 | 0.97 | 0.63 to 1.48 | 1.02 | 0.67 to 1.57 | |  | | 62 | 1.19 | 0.75 to 1.88 | 1.25 | 0.79 to 1.99 |
| Pancreas (C25) |  |  |  |  |  |  |  | |  | |  |  |  |  |  |
| < 20 | 0 |  | 1 |  |  |  |  | |  | | 1 |  |  |  |  |
| < 25 | 0 |  | 1 |  |  |  |  | |  | | 3 |  |  |  |  |
| < 30 | 0 |  | 8 |  |  |  |  | |  | | 3 |  |  |  |  |
| < 35 | 1 |  | 14 | 0.56 | 0.07 to 4.39 | 0.61 | 0.08 to 4.82 | |  | | 4 | 0.84 | 0.09 to 7.64 | 0.80 | 0.09 to 7.26 |
| < 40 | 1 |  | 17 | 0.45 | 0.06 to 3.51 | 0.50 | 0.06 to 3.88 | |  | | 8 | 0.36 | 0.05 to 2.92 | 0.37 | 0.05 to 2.98 |
| ≥ 40 | 11 |  | 42 | 1.68 | 0.84 to 3.36 | 1.72 | 0.85 to 3.46 | |  | | 19 | 1.69 | 0.80 to 3.56 | 1.75 | 0.83 to 3.72 |
| Breast (C50) |  |  |  |  |  |  |  | |  | |  |  |  |  |  |
| < 20 | 16 |  | 62 | 1.29 | 0.73 to 2.29 | 1.49 | 0.83 to 2.65 | |  | | 33 | 1.41 | 0.78 to 2.57 | 1.55 | 0.85 to 2.84 |
| < 25 | 29 |  | 117 | 1.49 | 0.98 to 2.29 | **1.70** | **1.11 to 2.62** | |  | | 63 | 1.53 | 0.98 to 2.39 | **1.60** | **1.03 to 2.49** |
| < 30 | 40 |  | 180 | 1.32 | 0.92 to 1.89 | **1.46** | **1.02 to 2.09** | |  | | 108 | 1.18 | 0.82 to 1.70 | 1.27 | 0.88 to 1.84 |
| < 35 | 59 |  | 270 | 1.22 | 0.91 to 1.63 | 1.31 | 0.98 to 1.76 | |  | | 142 | 1.27 | 0.94 to 1.73 | 1.34 | 0.99 to 1.82 |
| < 40 | 81 |  | 395 | 1.11 | 0.87 to 1.43 | 1.21 | 0.94 to 1.55 | |  | | 177 | **1.33** | **1.02 to 1.74** | **1.42** | **1.09 to 1.85** |
| ≥ 40 | 77 |  | 336 | **1.34** | **1.04 to 1.74** | **1.35** | **1.04 to 1.75** | |  | | 144 | **1.47** | **1.11 to 1.94** | **1.50** | **1.14 to 1.99** |
| Kidney (C64-C66) |  |  |  |  |  |  |  | |  | |  |  |  |  |  |
| < 20 | 1 |  | 3 | 1.53 | 0.14 to 16.29 | 1.46 | 1.14 to 15.65 | |  | | 1 | 3.08 | 0.19 to 51.25 | 3.12 | 0.18 to 54.04 |
| < 25 | 1 |  | 7 | 1.02 | 0.12 to 9.01 | 1.20 | 0.13 to 10.89 | |  | | 5 | 0.77 | 0.09 to 6.70 | 0.64 | 0.07 to 5.58 |
| < 30 | 3 |  | 14 | 1.44 | 0.39 to 5.34 | 1.57 | 0.41 to 5.95 | |  | | 10 | 1.01 | 0.28 to 3.69 | 1.01 | 0.27 to 3.71 |
| < 35 | 7 |  | 24 | 1.72 | 0.71 to 4.18 | 1.87 | 0.76 to 4.57 | |  | | 12 | 1.87 | 0.73 to 4.76 | 1.88 | 0.74 to 4.82 |
| < 40 | 7 |  | 32 | 1.26 | 0.54 to 2.96 | 1.36 | 0.58 to 3.20 | |  | | 17 | 1.24 | 0.51 to 3.00 | 1.30 | 0.54 to 3.17 |
| ≥ 40 | 14 |  | 62 | 1.58 | 0.86 to 2.90 | 1.79 | 0.97 to 3.29 | |  | | 24 | 1.59 | 0.82 to 3.08 | 1.90 | 0.97 to 3.72 |
| Lymphoid and haematopoietic tissue (LH) (C81-C96, D45-D47) |  |  |  |  |  |  |  | |  | |  |  |  |  |  |
| < 20 | 4 |  | 13 | 1.47 | 0.46 to 4.70 | 1.57 | 0.49 to 5.06 | |  | | 7 | 2.01 | 0.58 to 6.98 | 1.93 | 0.55 to 6.72 |
| < 25 | 9 |  | 20 | **2.51** | **1.09 to 5.79** | **2.70** | **1.17 to 6.25** | |  | | 15 | 2.04 | 0.88 to 4.74 | 1.97 | 0.85 to 4.58 |
| < 30 | 13 |  | 42 | 1.89 | 0.98 to 3.64 | **2.10** | **1.09 to 4.05** | |  | | 17 | **2.68** | **1.29 to 5.57** | **2.66** | **1.27 to 5.56** |
| < 35 | 17 |  | 54 | **1.91** | **1.07 to 3.39** | **2.06** | **1.16 to 3.68** | |  | | 21 | **2.77** | **1.45 to 5.29** | **2.73** | **1.42 to 5.22** |
| < 40 | 19 |  | 68 | 1.62 | 0.95 to 2.76 | **1.79** | **1.05 to 3.05** | |  | | 29 | **2.00** | **1.12 to 3.59** | **2.01** | **1.12 to 3.61** |
| ≥ 40 | 23 |  | 98 | 1.28 | 0.80 to 2.04 | 1.38 | 0.86 to 2.22 | |  | | 52 | 1.28 | 0.78 to 2.09 | 1.31 | 0.80 to 2.15 |
| Non-Hodgkin´s lymphoma (NHL) (C82-C85) |  |  |  |  |  |  |  | |  | |  |  |  |  |  |
| < 20 | 1 |  | 1 | 4.67 | 0.26 to 83.93 | 4.98 | 0.26 to 93.94 | |  | | 2 | 1.55 | 0.14 to 17.43 | 1.39 | 0.12 to 15.77 |
| < 25 | 3 |  | 5 | **4.93** | **1.06 to 22.85** | **5.22** | **1.12 to 24.28** | |  | | 2 | **6.44** | **1.04 to 39.99** | 5.61 | 0.91 to 34.51 |
| < 30 | 7 |  | 13 | **3.61** | **1.35 to 9.66** | **3.96** | **1.48 to 10.56** | |  | | 4 | **6.96** | **2.01 to 24.16** | **7.42** | **2.09 to 26.34** |
| < 35 | 9 |  | 16 | **3.83** | **1.59 to 9.28** | **4.15** | **1.71 to 10.09** | |  | | 4 | **8.53** | **2.59 to 28.10** | **8.52** | **2.57 to 28.27** |
| < 40 | 9 |  | 20 | **2.92** | **1.26 to 6.77** | **3.25** | **1.40 to 7.55** | |  | | 6 | **5.56** | **1.95 to 15.88** | **5.64** | **1.96 to 16.21** |
| ≥ 40 | 6 |  | 35 | 1.08 | 0.44 to 2.64 | 1.22 | 0.49 to 3.00 | |  | | 10 | 1.75 | 0.63 to 4.84 | 1.88 | 0.67 to 5.30 |
| **Not included in all cancers** | | | | | | | | | | | | | | | |
| Basal cell carcinoma of the skin (C44) |  |  |  |  |  |  |  | |  | |  |  |  |  |  |
| < 20 | 8 |  | 47 | 0.85 | 0.40 to 1.84 | 0.96 | 0.44 to 2.09 | |  | | 24 | 0.94 | 0.42 to 2.09 | 1.06 | 0.48 to 2.37 |
| < 25 | 15 |  | 68 | 1.10 | 0.62 to 1.95 | 1.22 | 0.68 to 2.18 | |  | | 35 | 1.28 | 0.70 to 2.36 | 1.43 | 0.78 to 2.63 |
| < 30 | 25 |  | 97 | 1.26 | 0.80 to 1.99 | 1.37 | 0.87 to 2.16 | |  | | 49 | 1.52 | 0.94 to 2.47 | **1.67** | **1.03 to 2.72** |
| < 35 | 29 |  | 132 | 1.11 | 0.73 to 1.68 | 1.20 | 0.79 to 1.82 | |  | | 66 | 1.31 | 0.85 to 2.03 | 1.43 | 0.92 to 2.23 |
| < 40 | 42 |  | 181 | 1.17 | 0.82 to 1.65 | 1.26 | 0.89 to 1.79 | |  | | 91 | 1.30 | 0.90 to 1.87 | 1.40 | 0.97 to 2.03 |
| ≥ 40 | 54 |  | 256 | 1.18 | 0.87 to 1.60 | 1.23 | 0.91 to 1.67 | |  | | 98 | **1.54** | **1.10 to 2.15** | **1.62** | **1.16 to 2.27** |

Table N. Number of selected cancer sites among men and women combined in the geothermal heating areas, hazard ratio (HR), 95% confidence intervals (CI) compared with the populations in warm reference area and cold reference area, applying five years latency time, adjusted for age, gender, education, type of housing, and smoking habits, without and with stratification into categories of cumulative years of residence, restricted on different age categories in the respective areas.

|  | Geothermal heating area |  | Warm reference area | | | | |  | | Cold reference area | | | | | |
| --- | --- | --- | --- | --- | --- | --- | --- | --- | --- | --- | --- | --- | --- | --- | --- |
|  | No of |  | No of | Not stratified | | Stratified | | |  | | No of | Not stratified | | Stratified | |
| Cancers (ICD-10) | cancers |  | cancers | HR | 95%CI | HR | 95%CI | |  | | cancers | HR | 95%CI | HR | 95%CI |
| All (C00-C97, D45-D47) |  |  |  |  |  |  |  | |  | |  |  |  |  |  |
| < 20 | 40 |  | 214 | 1.03 | 0.73 to 1.47 | 1.11 | 0.78 to 1.58 | |  | | 113 | 1.05 | 0.73 to 1.51 | 1.08 | 0.75 to 1.56 |
| < 25 | 64 |  | 350 | 1.08 | 0.82 to 1.42 | 1.15 | 0.87 to 1.52 | |  | | 183 | 1.13 | 0.85 to 1.51 | 1.15 | 0.86 to 1.53 |
| < 30 | 99 |  | 537 | 1.09 | 0.87 to 1.36 | 1.16 | 0.93 to 1.45 | |  | | 292 | 1.10 | 0.88 to 1.39 | 1.13 | 0.90 to 1.43 |
| < 35 | 148 |  | 780 | 1.08 | 0.90 to 1.30 | 1.14 | 0.95 to 1.37 | |  | | 403 | 1.18 | 0.97 to 1.42 | 1.20 | 0.99 to 1.45 |
| < 40 | 195 |  | 1053 | 1.02 | 0.87 to 1.19 | 1.08 | 0.92 to 1.27 | |  | | 536 | 1.11 | 0.95 to 1.31 | 1.14 | 0.96 to 1.34 |
| ≥ 40 | 177 |  | 792 | **1.26** | **1.06 to 1.49** | **1.29** | **1.09 to 1.52** | |  | | 423 | **1.27** | **1.06 to 1.51** | **1.30** | **1.09 to 1.55** |
| Colon, rectum, and anus (C18-C21) |  |  |  |  |  |  |  | |  | |  |  |  |  |  |
| < 20 | 2 |  | 9 | 1.47 | 0.28 to 7.57 | 1.49 | 0.29 to 7.69 | |  | | 3 | 1.75 | 0.29 to10.60 | 1.81 | 0.30 to 11.06 |
| < 25 | 3 |  | 19 | 0.98 | 0.27 to 3.48 | 1.04 | 0.29 to 3.71 | |  | | 6 | 1.89 | 0.46 to 7.80 | 1.92 | 0.47 to 7.86 |
| < 30 | 6 |  | 33 | 1.19 | 0.48 to 2.96 | 1.32 | 0.53 to 3.29 | |  | | 12 | 1.70 | 0.63 to 4.55 | 1.77 | 0.66 to 4.76 |
| < 35 | 10 |  | 49 | 1.22 | 0.60 to 2.48 | 1.31 | 0.64 to 2.68 | |  | | 24 | 1.38 | 0.66 to 2.89 | 1.43 | 0.68 to 3.00 |
| < 40 | 12 |  | 69 | 1.09 | 0.57 to 2.07 | 1.21 | 0.63 to 2.30 | |  | | 41 | 0.93 | 0.48 to 1.78 | 0.96 | 0.50 to 1.85 |
| ≥ 40 | 20 |  | 94 | 1.15 | 0.70 to 1.89 | 1.17 | 0.71 to 1.93 | |  | | 37 | 1.62 | 0.94 to 2.79 | 1.63 | 0.94 to 2.82 |
| Pancreas (C25) |  |  |  |  |  |  |  | |  | |  |  |  |  |  |
| < 20 | 0 |  | 1 |  |  |  |  | |  | | 2 |  |  |  |  |
| < 25 | 0 |  | 1 |  |  |  |  | |  | | 5 |  |  |  |  |
| < 30 | 1 |  | 12 | 0.51 | 0.06 to 4.14 | 0.55 | 0.07 to 4.46 | |  | | 5 | 0.64 | 0.07 to 5.55 | 0.62 | 0.07 to 5.40 |
| < 35 | 3 |  | 19 | 0.94 | 0.27 to 3.30 | 1.00 | 0.28 to 3.54 | |  | | 7 | 1.45 | 0.37 to 5.61 | 1.47 | 0.38 to 5.74 |
| < 40 | 5 |  | 25 | 1.34 | 0.49 to 3.66 | 1.45 | 0.53 to 3.99 | |  | | 12 | 1.36 | 0.48 to 3.89 | 1.40 | 0.49 to 4.02 |
| ≥ 40 | 6 |  | 11 | **3.30** | **1.15 to 9.44** | **3.83** | **1.30 to 11.28** | |  | | 7 | **3.08** | **1.01 to 9.36** | **3.40** | **1.09 to 10.63** |
| Breast (C50) |  |  |  |  |  |  |  | |  | |  |  |  |  |  |
| < 20 | 12 |  | 50 | 1.19 | 0.62 to 2.30 | 1.34 | 0.69 to 2.60 | |  | | 23 | 1.51 | 0.75 to 3.05 | 1.60 | 0.79 to 3.24 |
| < 25 | 20 |  | 80 | 1.39 | 0.83 to 2.32 | 1.52 | 0.91 to 2.55 | |  | | 43 | 1.56 | 0.92 to 2.68 | 1.57 | 0.92 to 2.68 |
| < 30 | 29 |  | 106 | 1.49 | 0.97 to 2.29 | **1.59** | **1.03 to 2.46** | |  | | 66 | 1.42 | 0.92 to 2.21 | 1.47 | 0.94 to 2.28 |
| < 35 | 35 |  | 143 | 1.33 | 0.91 to 1.96 | 1.41 | 0.96 to 2.08 | |  | | 80 | 1.37 | 0.92 to 2.04 | 1.38 | 0.93 to 2.06 |
| < 40 | 42 |  | 203 | 1.09 | 0.77 to 1.53 | 1.14 | 0.80 to 1.60 | |  | | 95 | 1.32 | 0.92 to 1.90 | 1.34 | 0.93 to 1.93 |
| ≥ 40 | 14 |  | 74 | 1.16 | 0.65 to 2.10 | 1.17 | 0.65 to 2.11 | |  | | 38 | 1.10 | 0.59 to 2.03 | 1.09 | 0.59 to 2.02 |
| Prostate (C61) |  |  |  |  |  |  |  | |  | |  |  |  |  |  |
| < 20 | 4 |  | 5 | **4.61** | **1.08 to19.68** | **5.28** | **1.21 to23.00** | |  | | 1 | **11.07** | **1.24 to99.28** | **11.81** | **1.31 to106.50** |
| < 25 | 5 |  | 14 | 2.13 | 0.71 to 6.34 | 2.28 | 0.76 to 6.84 | |  | | 7 | 2.39 | 0.74 to 7.67 | 2.50 | 0.77 to 8.04 |
| < 30 | 10 |  | 27 | 2.12 | 0.98 to 4.58 | **2.31** | **1.07 to 5.02** | |  | | 21 | 1.62 | 0.75 to 3.48 | 1.73 | 0.80 to 3.73 |
| < 35 | 15 |  | 73 | 1.10 | 0.61 to 1.96 | 1.21 | 0.67 to 2.17 | |  | | 44 | 1.13 | 0.62 to 2.04 | 1.23 | 0.67 to 2.23 |
| < 40 | 24 |  | 134 | 0.96 | 0.61 to 1.51 | 1.07 | 0.68 to 1.68 | |  | | 68 | 1.14 | 0.71 to 1.83 | 1.20 | 0.75 to 1.93 |
| ≥ 40 | 33 |  | 154 | 1.38 | 0.93 to 2.06 | 1.40 | 0.94 to 2.09 | |  | | 70 | 1.47 | 0.97 to 2.24 | 1.50 | 0.98 to 2.28 |
| Kidney (C64-C66) |  |  |  |  |  |  |  | |  | |  |  |  |  |  |
| < 20 | 2 |  | 12 | 1.10 | 0.23 to 5.30 | 1.23 | 0.26 to 5.97 | |  | | 4 | 2.45 | 0.35 to 14.24 | 2.46 | 0.38 to 15.74 |
| < 25 | 2 |  | 16 | 0.77 | 0.17 to 3.55 | 0.83 | 0.18 to 3.84 | |  | | 8 | 0.82 | 0.17 to 3.90 | 0.81 | 0.17 to 3.81 |
| < 30 | 2 |  | 29 | 0.43 | 0.10 to 1.85 | 0.44 | 0.10 to 1.89 | |  | | 16 | 0.41 | 0.09 to 1.79 | 0.41 | 0.09 to 1.79 |
| < 35 | 6 |  | 37 | 0.97 | 0.40 to 2.40 | 1.00 | 0.41 to 2.47 | |  | | 18 | 1.07 | 0.43 to 2.71 | 1.08 | 0.43 to 2.72 |
| < 40 | 8 |  | 51 | 0.83 | 0.38 to 1.80 | 0.86 | 0.40 to 1.87 | |  | | 25 | 0.96 | 0.43 to 2.13 | 0.97 | 0.44 to 2.15 |
| ≥ 40 | 8 |  | 39 | 1.32 | 0.60 to 2.91 | 1.34 | 0.60 to 2.98 | |  | | 16 | 1.48 | 0.63 to 3.46 | 1.48 | 0.63 to 3.48 |
| Lymphoid and haematopoietic tissue (LH) (C81-C96, D45-D47) |  |  |  |  |  |  |  | |  | |  |  |  |  |  |
| < 20 | 1 |  | 15 | 0.34 | 0.04 to 2.68 | 0.37 | 0.05 to 2.90 | |  | | 14 | 0.27 | 0.04 to 2.13 | 0.29 | 0.04 to 2.25 |
| < 25 | 4 |  | 21 | 1.03 | 0.34 to 3.12 | 1.11 | 0.37 to 3.36 | |  | | 23 | 0.62 | 0.21 to 1.82 | 0.63 | 0.21 to 1.84 |
| < 30 | 6 |  | 43 | 0.90 | 0.37 to 2.17 | 0.96 | 0.40 to 2.33 | |  | | 28 | 0.79 | 0.32 to 1.95 | 0.79 | 0.32 to 1.95 |
| < 35 | 11 |  | 62 | 1.07 | 0.55 to 2.09 | 1.14 | 0.58 to 2.22 | |  | | 34 | 1.20 | 0.60 to 2.41 | 1.20 | 0.60 to 2.42 |
| < 40 | 13 |  | 81 | 0.92 | 0.50 to 1.69 | 1.00 | 0.55 to 1.84 | |  | | 41 | 1.07 | 0.57 to 2.02 | 1.08 | 0.57 to 2.03 |
| ≥ 40 | 24 |  | 62 | **2.24** | **1.36 to 3.68** | **2.46** | **1.48 to 4.07** | |  | | 31 | **2.34** | **1.37 to 4.00** | **2.67** | **1.55 to 4.60** |
| Non-Hodgkin´s lymphoma (NHL) (C82-C85) |  |  |  |  |  |  |  | |  | |  |  |  |  |  |
| < 20 | 1 |  | 5 | 1.12 | 0.12 to 10.61 | 1.24 | 0.13 to 11.82 | |  | | 5 | 0.80 | 0.09 to 7.38 | 0.83 | 0.09 to 7.65 |
| < 25 | 2 |  | 9 | 1.26 | 0.26 to 6.22 | 1.39 | 0.28 to 6.86 | |  | | 9 | 0.96 | 0.19 to 4.78 | 0.96 | 0.19 to 4.80 |
| < 30 | 4 |  | 17 | 1.29 | 0.41 to 4.00 | 1.41 | 0.45 to 4.42 | |  | | 12 | 1.42 | 0.43 to 4.68 | 1.41 | 0.43 to 4.68 |
| < 35 | 8 |  | 24 | 1.80 | 0.77 to 4.22 | 1.93 | 0.82 to 4.53 | |  | | 13 | 2.40 | 0.95 to 6.09 | 2.40 | 0.94 to 6.10 |
| < 40 | 9 |  | 29 | 1.67 | 0.76 to 3.69 | 1.86 | 0.84 to 4.11 | |  | | 13 | **2.55** | **1.04 to 6.25** | **2.53** | **1.03 to 6.22** |
| ≥ 40 | 8 |  | 20 | **2.76** | **1.15 to 6.63** | **2.98** | **1.23 to 7.20** | |  | | 7 | **3.56** | **1.23 to10.25** | **3.76** | **1.28 to 11.02** |
| **Not included in all cancers** | | | | | | | | | | | | | | | |
| Basal cell carcinoma of the skin (C44) |  |  |  |  |  |  |  | |  | |  |  |  |  |  |
| < 20 | 13 |  | 54 | 1.27 | 0.68 to 2.37 | 1.40 | 0.75 to 2.62 | |  | | 25 | 1.54 | 0.78 to 3.02 | 1.64 | 0.83 to 3.24 |
| < 25 | 22 |  | 84 | 1.44 | 0.88 to 2.33 | 1.57 | 0.97 to 2.56 | |  | | 37 | **1.89** | **1.11 to 3.22** | **2.07** | **1.21 to 3.54** |
| < 30 | 33 |  | 116 | **1.54** | **1.04 to 2.30** | **1.68** | **1.13 to 2.51** | |  | | 54 | **1.95** | **1.26 to 3.03** | **2.10** | **1.35 to 3.26** |
| < 35 | 40 |  | 155 | 1.37 | 0.97 to 1.95 | **1.49** | **1.04 to 2.13** | |  | | 72 | **1.76** | **1.19 to 2.60** | **1.86** | **1.26 to 2.76** |
| < 40 | 48 |  | 187 | 1.36 | 0.98 to 1.89 | **1.48** | **1.07 to 2.06** | |  | | 91 | **1.61** | **1.13 to 2.29** | **1.69** | **1.19 to 2.41** |
| ≥ 40 | 26 |  | 170 | 0.87 | 0.57 to 1.32 | 0.87 | 0.57 to 1.33 | |  | | 63 | 1.26 | 0.79 to 1.99 | 1.28 | 0.80 to 2.03 |

Table O. Number of individuals, number of male breast cancer cases, number of individuals with and without mutation of the BRCA2 gene, the prevalence of those with, and without the mutation according to Thorlacius et al. [31], and predictive values for breast cancer among females according to the method of Axelson and Steenland [29], and using the relative risk for breast cancer among female according to Thorlacius et al. [32], in the geothermal area in comparison with warm and cold reference areas, and the combined capital area and Reykjanes.

|  |  |  | The mutation of BRCA2 gene | | | |  |  |
| --- | --- | --- | --- | --- | --- | --- | --- | --- |
| Areas | No of individuals | No male breast cancer | Estimated number with BRCA2 | Estimated number without BRCA2 | Estimated prevalence with BRCA2 | Estimated prevalence without BRCA2 | Risk of female breast cancer | Predictive values (risk ratio) |
| Geothermal heating area | 7511 | 3 | 87 | 7424 | 1.16 | 98.84 | 106.97 |  |
| Warm reference area | 44 864 | 9 | 262 | 44 602 | 0.58 | 99.42 | 103.50 | 1.03 |
| Cold reference area | 22 431 | 5 | 145 | 22 286 | 0.65 | 99.35 | 103.89 | 1.03 |
| Reykjavik capital area and Reykjanes | 109 308 | 21 | 610 | 108 698 | 0.56 | 99.44 | 103.35 | 1.03 |
| Census | 184 114 | 38 | 1105 | 183 009 | 0.60 | 99.40 | 103.60 | NC^1^ |

^1^ NC, not compared.
